# Supplementary material for: Risk factors for prostate cancer: An umbrella review of prospective observational studies and mendelian randomization analyses
Source: PLoS Med. 2024 Mar 15;21(3):e1004362. doi: 10.1371/journal.pmed.1004362 (PMC10980219; doi:10.1371/journal.pmed.1004362)
Supplement: S1 Text — (DOCX) [file pmed.1004362.s002.docx]

**S1 Text - Search strategies**

(1) For meta-analyses

((({Meta-analysis}[Title/Abstract] OR {meta-analyses}[Title/Abstract] OR {meta}[Title/Abstract] OR {systematic review}[Title/Abstract] OR {literature review}[Title/Abstract] OR {review}[Title/Abstract] OR {pooled analysis}[Title/Abstract] OR {pooled analyses}[Title/Abstract] OR {pooled}[Title/Abstract] OR {consortium}[Title/Abstract] OR {consortia}[Title/Abstract] OR {collaboration}[Title/Abstract]) AND ({prostate cancer}[Title/Abstract] OR {cancer of the prostate}[Title/Abstract] OR {prostate adenocarcinoma} [Title/Abstract] OR {adenocarcinoma of the prostate}[Title/Abstract] OR {prostate carcinoma}[Title/Abstract] OR {carcinoma of the prostate}[Title/Abstract] OR {prostate tumor}[Title/Abstract] OR {prostate malignancy}[Title/Abstract] OR {prostate malignancies}[Title/Abstract] OR {prostate neoplasm}[Title/Abstract] OR {prostate neoplasia}[Title/Abstract])) AND ({Cohort} OR {cohort study} OR {nested case-control} OR {trial} OR {prospective} OR {follow-up} OR {registry} OR {record linkage} OR {longitudinal} OR {incidence})) AND ({Odds ratio} OR {OR} OR {hazard risk} OR {hazard ratio} OR {HR} OR {relative risk} OR {RR} OR {rate ratio} OR {P} OR {P value} OR {P=} OR {association} OR {associated} OR {confidence interval} OR {CI} OR {censor} OR {Kaplan-Meier} OR {Cox model} OR {Proportional hazard model} OR {log-rank} OR {survival analysis})

(2) For mendelian randomization studies

({prostate cancer}[Title/Abstract] OR {cancer of the prostate}[Title/Abstract] OR {prostate adenocarcinoma}[Title/Abstract] OR {adenocarcinoma of the prostate}[Title/Abstract] OR {prostate carcinoma}[Title/Abstract] OR {carcinoma of the prostate}[Title/Abstract] OR {prostate tumor}[Title/Abstract] OR {prostate malignancy}[Title/Abstract] OR {prostate malignancies}[Title/Abstract] OR {prostate neoplasm}[Title/Abstract] OR {prostate neoplasia}[Title/Abstract]) AND ({Mendelian randomization}[Title/Abstract] OR {Mendelian randomisation}[Title/Abstract] OR {instrumental variable}[Title/Abstract] OR {causal inference}[Title/Abstract] OR {causal association}[Title/Abstract] OR {causal relationship}[Title/Abstract])
