# Supplementary material for: Risk factors for prostate cancer: An umbrella review of prospective observational studies and mendelian randomization analyses
Source: PLoS Med. 2024 Mar 15;21(3):e1004362. doi: 10.1371/journal.pmed.1004362 (PMC10980219; doi:10.1371/journal.pmed.1004362)
Supplement: S2 Text — (DOCX) [file pmed.1004362.s003.docx]

**Statistical analysis protocol**

**1. Extracting study-specific data**

First, in each included meta-analysis, cross-sectional studies and case-control studies with unclear temporality were excluded.

Second, in each included meta-analysis, qualified individual studies (cohort, case-cohort, or nested case-control study where exposure precedes the outcome) were selected, and their following information were collected based on a predefined template: first author, publication year, study design, number of studies included, number of cases/population, ethnicity, exposure factors, outcomes of prostate cancer, comparisons, and effect estimates of any type, i.e., maximally adjusted hazard ratio (HR)/incidence rate ratio (IRR)/odds ratio (OR)/RR with 95% confidence intervals, i.e., lower confidence interval (LCI) and upper confidence interval (UCI).

**2. Repeating meta-analysis using random-effects inverse-variance model with DerSimonian-Laird**

Fixed-effect model and the random-effects model are the two commonly applied statistical models for meta-analysis [1]. The fixed-effect model assumes all studies in the meta-analysis share a common true effect size, while the random-effects model involves an assumption that the effects being estimated in the different studies follow some distribution, which is more plausible for studies in the meta-analysis usually came from published literatures. A random-effects model provides a result that may be viewed as an “average intervention effect”, where this average is explicitly defined according to an assumed distribution of effects across studies. The assumption implies that the observed differences among study results are due to a combination of the play of chance and some genuine variation in the intervention effects. Therefore, we used the random-effects model in our umbrella review.

Following the umbrella review guideline, we repeated each meta-analysis based on extracted effect estimates, LCI, and UCI using the random-effects inverse-variance model with DerSimonian-Laird method.

When one meta-analysis investigated multiple exposures, we repeated the meta-analysis for each exposure separately.

**3. Assessing heterogeneity**

It is essential to consider the extent to which the results of studies are consistent with each other. If confidence intervals for the results of individual studies (generally depicted graphically using horizontal lines) have poor overlap, this generally indicates the presence of statistical heterogeneity. More formally, a statistical test for heterogeneity is available. The Chi^2^ (χ^2^, or chi-squared) test is included in the forest plots. It assesses whether observed differences in results are compatible with chance alone.

$$I^{2}=\left( Q-K-1 \right)/Q$$

In this equation, Q is the Chi^2^ statistic and K is its degrees of freedom [2]. I^2^ describes the percentage of the variability in effect estimates that is due to heterogeneity rather than sampling error (chance).

**4. Assessing small-study effects**

We used a random-effects Egger’s regression to examine whether there was an association between treatment effect size and its standard error, and whether smaller studies tended to show more pronounced effects than larger studies [3, 4]. If the Egger’s P value was less than 0.1, we assumed the existence of publication bias.

**5. Calculating prediction interval**

From random-effects meta-analysis, the summary estimate and its confidence interval only represent an estimate of the mean effect size and its precision. which are usually insufficient to summarize the association evidence with the presence of heterogeneity. The predictive distribution, which describes how the true effects are distributed around the mean effect, is a suitable statistical inference to be drawn from random-effects meta-analysis [5]. We characterized this random-effects distribution by using 95% prediction interval, in addition to the heterogeneity estimate. The 95% prediction interval––estimation of the middle 95% area of the effect distribution [6]––it predicts with 95% confidence the true effect in a new study similar to the studies in the meta-analysis. Note that the 95% PI was calculated for part of the factors for the sake of subsequent evidence grading which is restricted to significant associations.

All statistical analyses were conducted with the use of Stata, version 14.0 (StataCorp), and R, version 3.3.0 (R Foundation for Statistical Computing).

******Notes: STATA and R codes******

**The overall estimates and 95%CI are generated, with the heterogeneity estimate (I^2^) after running the following codes:**

*gen logOR=log(OR)*

*gen selogOR=(log(UCI)-log(LCI))/(2*1.96)*

*metan logOR selogOR, random eform*

**The egger’s P value is generated by the following STATA command:**

*metabias logOR selogOR, egger*

**The prediction interval is obtained from the forest plot after running the following codes:**

*library("meta")*

*dat1<-read.csv("occupational PA.csv")*

*lnor<-log(dat1[,"OR"])*

*lnuci<-log(dat1[,"UCI"])*

*lnlci<-log(dat1[,"LCI"])*

*selnor<-(lnuci-lnlci)/(2*1.96)*

*pfs=metagen(lnor,selnor, sm="OR",data=dat1,studlab=paste(dat1$Author, dat1$Year,sep="-"), prediction=TRUE, method.bias="Egger")*

*metabias(pfs, method.bias="Egger")*

*forest(pfs, prediction=TRUE)*

References:

1. Borenstein M, Hedges LV, Higgins JP, Rothstein HR. A basic introduction to fixed-effect and random-effects models for meta-analysis. Res Synth Methods. 2010;1(2):97-111.

2. Higgins JP, Thompson SG. Quantifying heterogeneity in a meta-analysis. Statistics in medicine. 2002;21(11):1539-58.

3. Sterne JA, Gavaghan D, Egger M. Publication and related bias in meta-analysis: power of statistical tests and prevalence in the literature. Journal of clinical epidemiology. 2000;53(11):1119-29.

4. Egger M, Davey Smith G, Schneider M, Minder C. Bias in meta-analysis detected by a simple, graphical test. BMJ. 1997;315(7109):629-34.

5. Higgins JP, Thompson SG, Spiegelhalter DJ. A re-evaluation of random-effects meta-analysis. J R Stat Soc Ser A Stat Soc. 2009;172(1):137-59.

6. Wang CC, Lee WC. A simple method to estimate prediction intervals and predictive distributions: Summarizing meta-analyses beyond means and confidence intervals. Res Synth Methods. 2019;10(2):255-66.
