# Supplementary material for: Risk factors for prostate cancer: An umbrella review of prospective observational studies and mendelian randomization analyses
Source: PLoS Med. 2024 Mar 15;21(3):e1004362. doi: 10.1371/journal.pmed.1004362 (PMC10980219; doi:10.1371/journal.pmed.1004362)

Category: Clinical variables, diseases, and treatments
Factor: Acne in adolescence
Comparison: patients vs non-patients

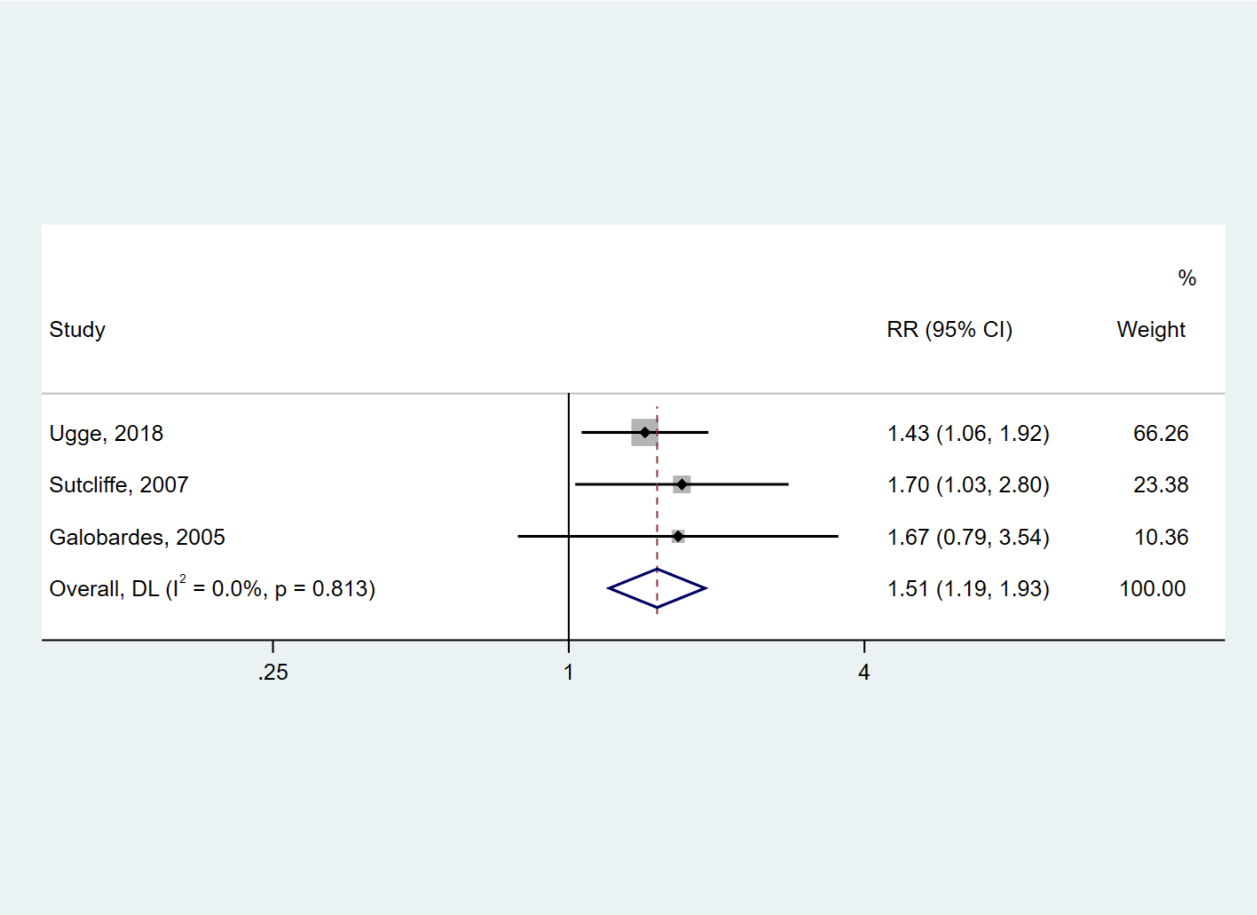

Category: Clinical variables, diseases, and treatments
Factor: Androgenic alopecia
Comparison: patients vs non-patients

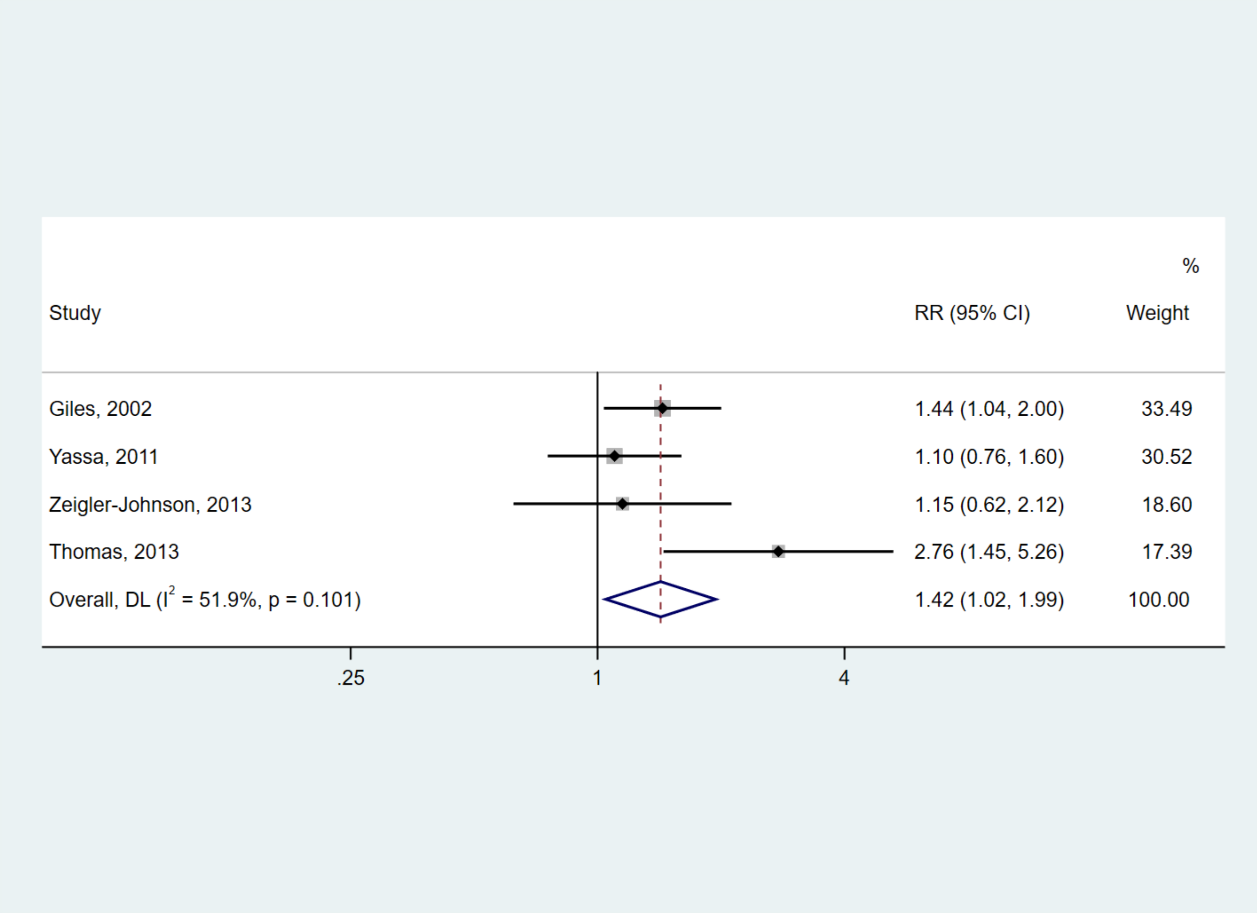

Category: Clinical variables, diseases, and treatments
Factor: Benign prostatic hyperplasia
Comparison: patients vs non-patients

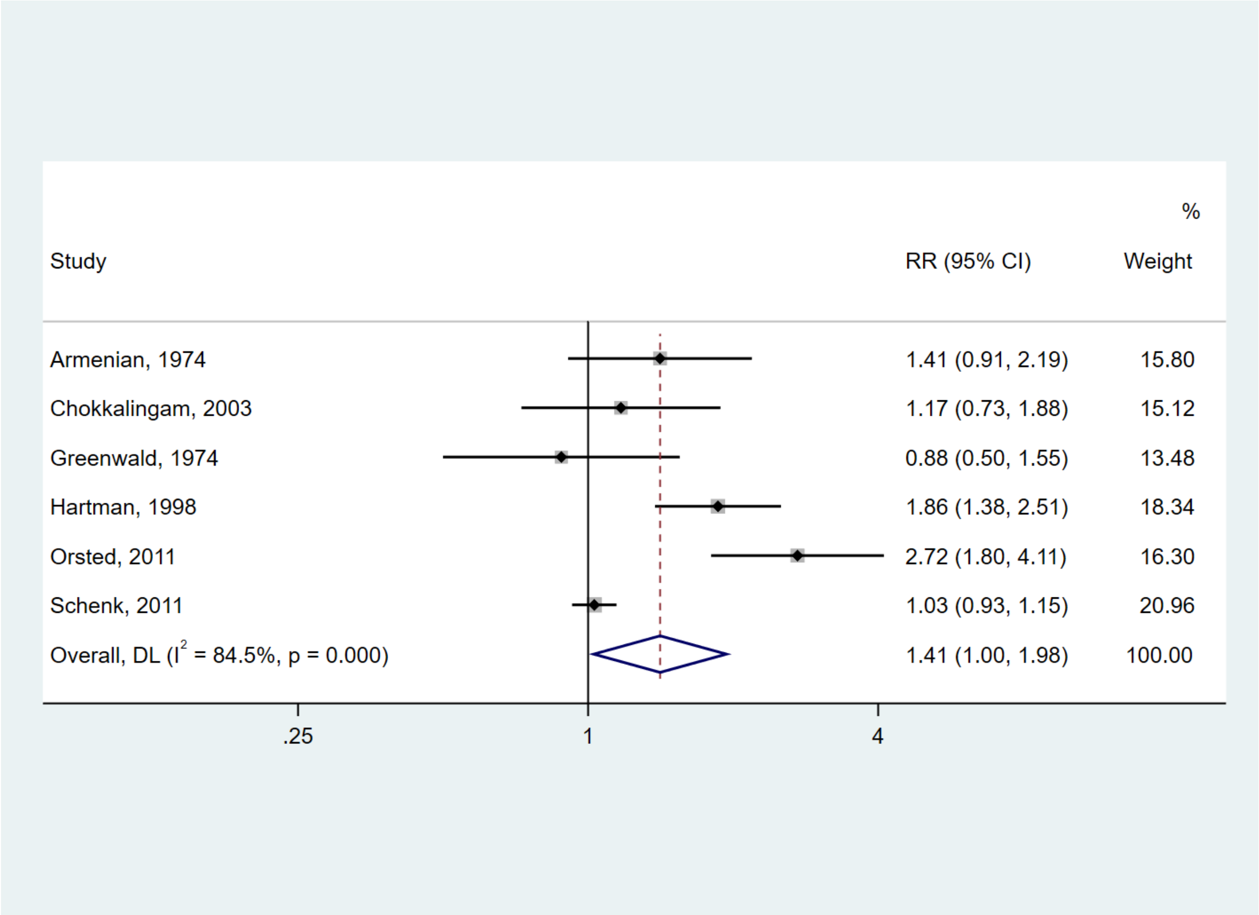

Category: Clinical variables, diseases, and treatments
Factor: First degree family breast cancer
Comparison: patients vs non-patients

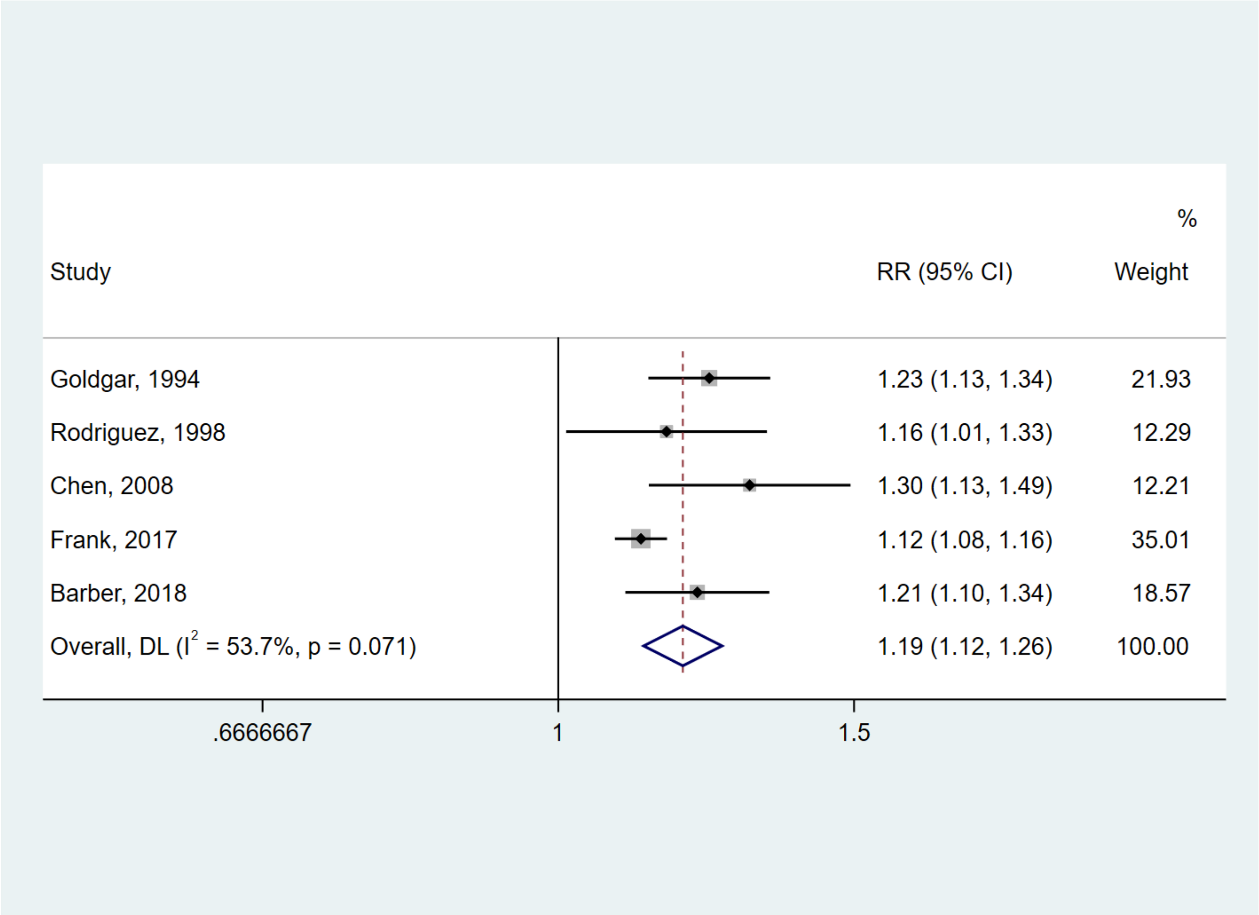

Category: Clinical variables, diseases, and treatments
Factor: HIV
Comparison: patients vs non-patients

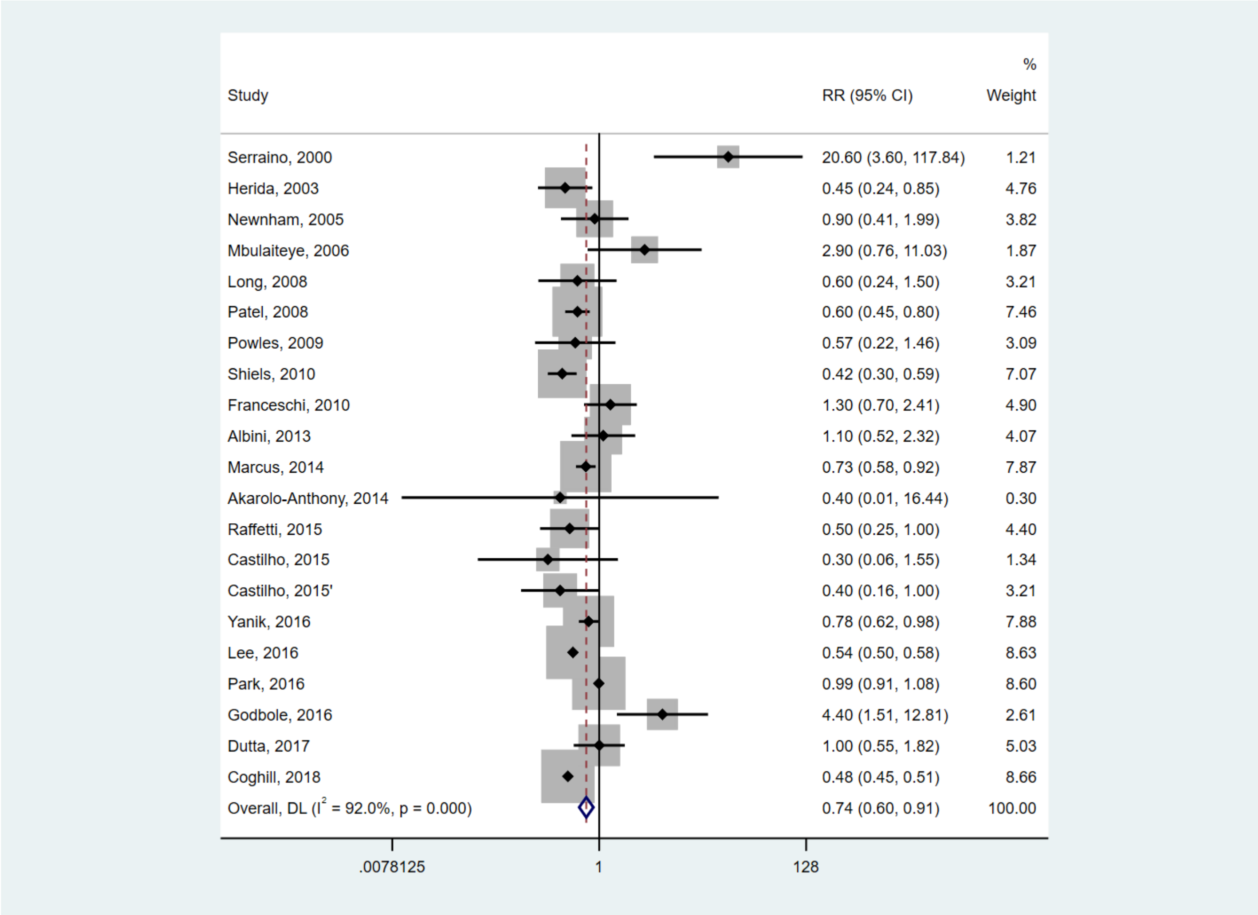

Category: Clinical variables, diseases, and treatments
Factor: Infertility
Comparison: infertile vs fertile

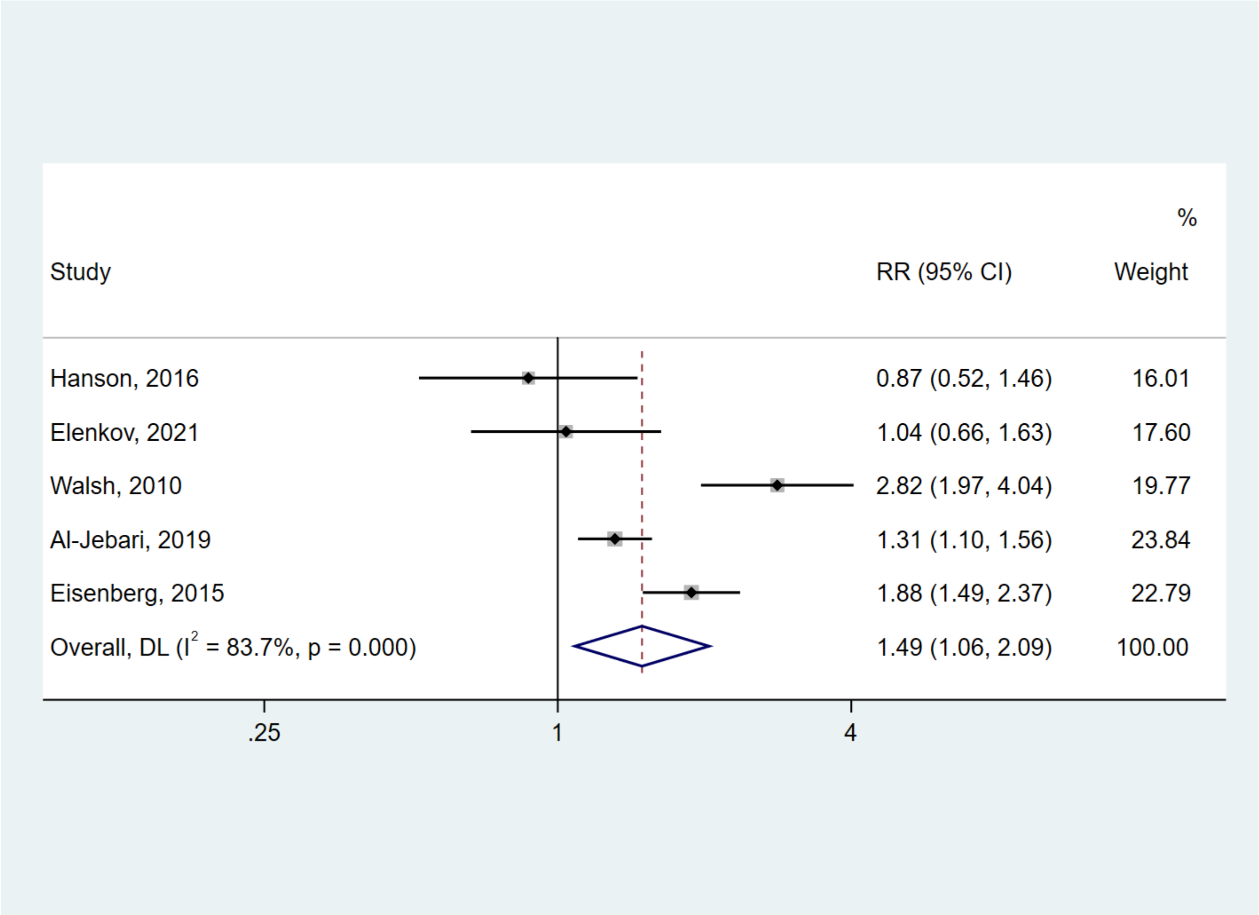

Category: Clinical variables, diseases, and treatments
Factor: Melanoma
Comparison: patients vs non-patients

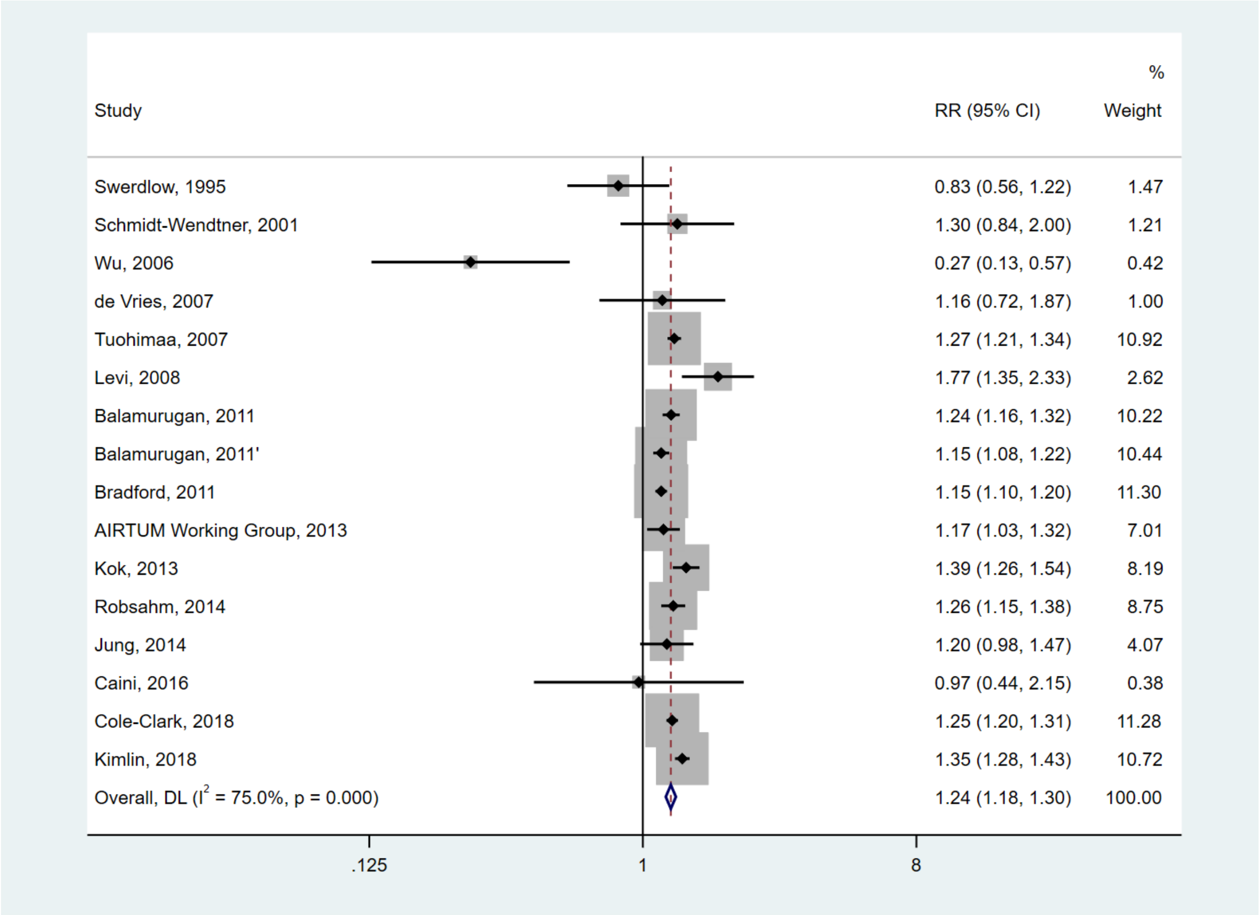

Category: Clinical variables, diseases, and treatments
Factor: Parkinson’s disease
Comparison: patients vs non-patients

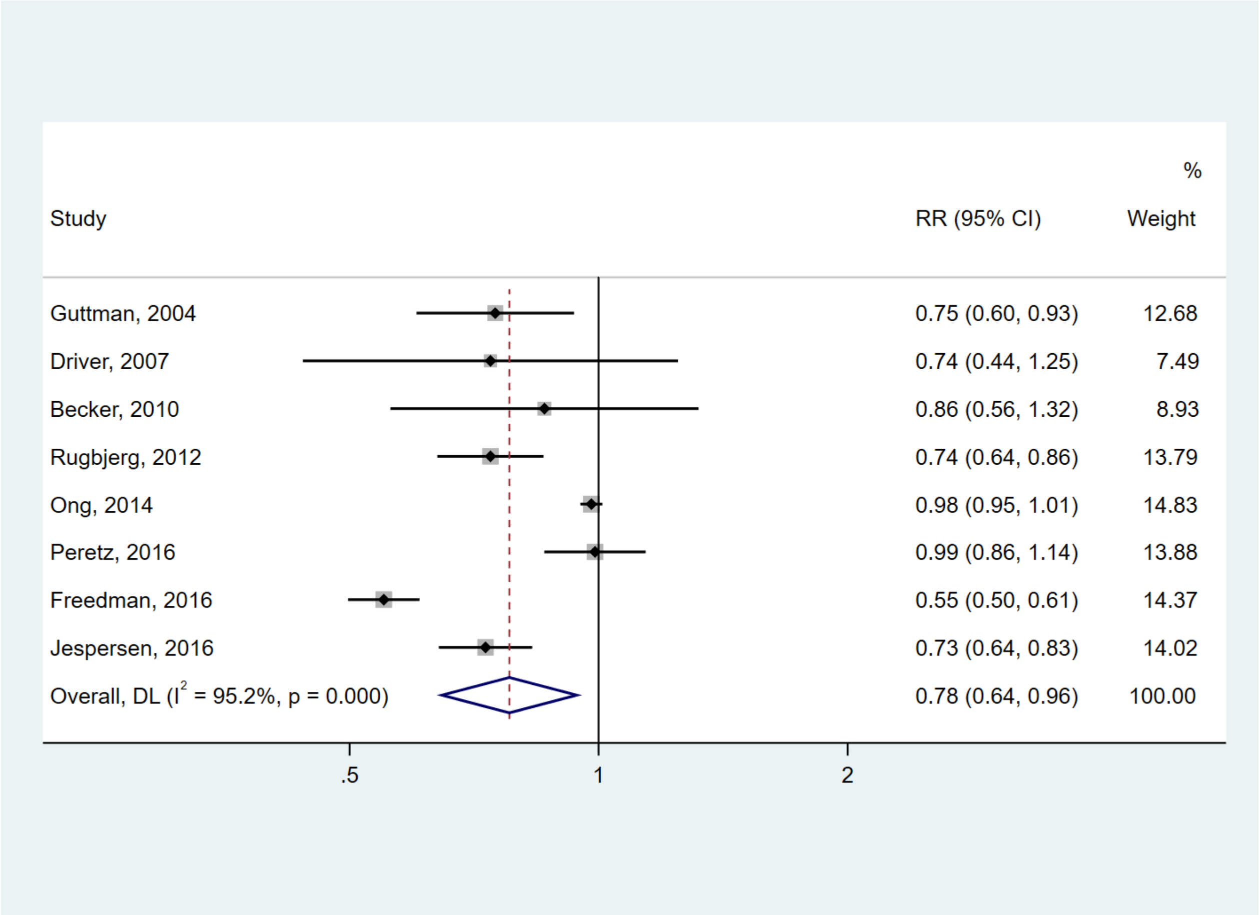

Category: Clinical variables, diseases, and treatments
Factor: Primary Sjögren's syndrome
Comparison: patients vs non-patients

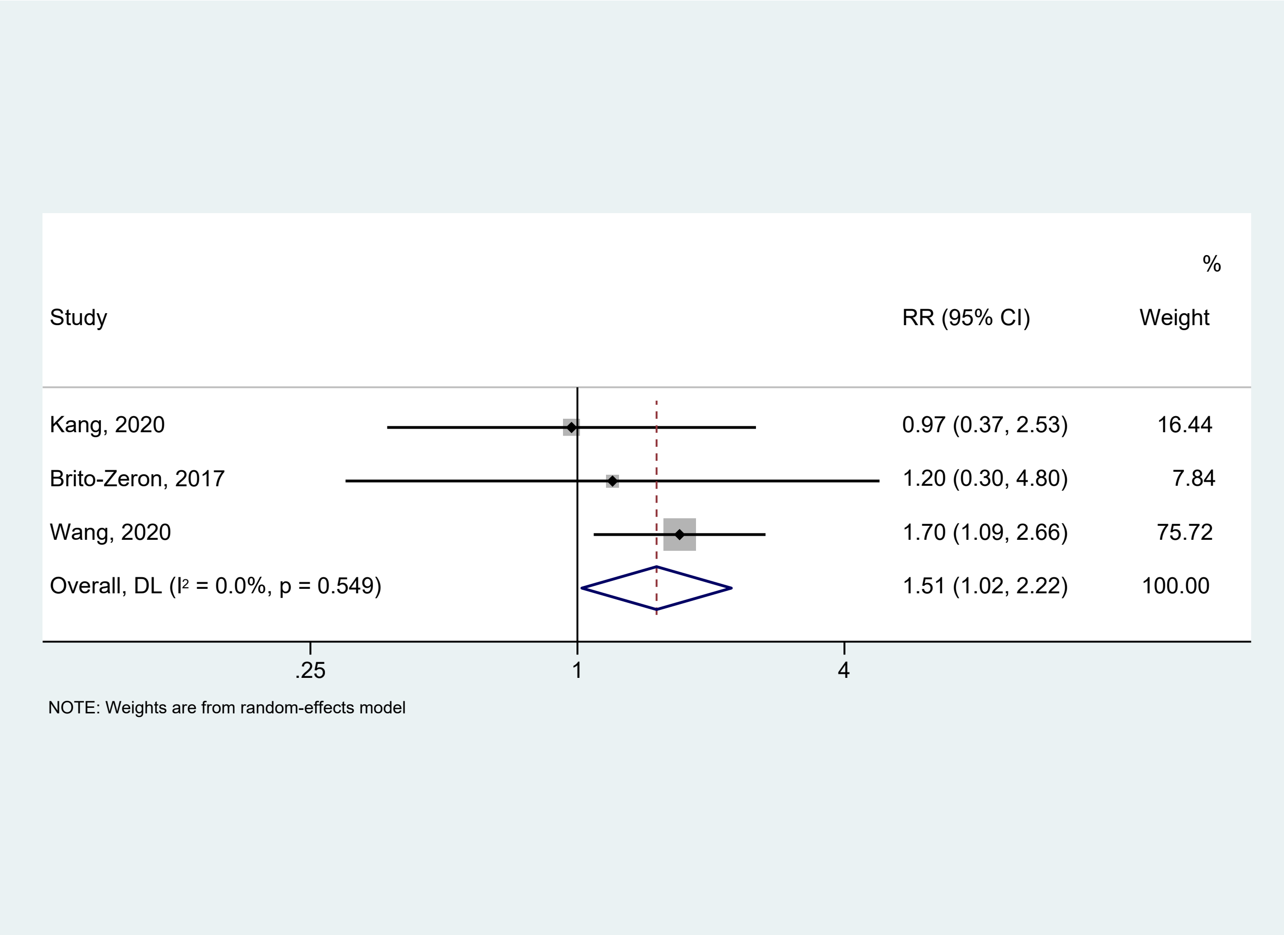

Category: Clinical variables, diseases, and treatments
Factor: Prostatitis
Comparison: patients vs non-patients

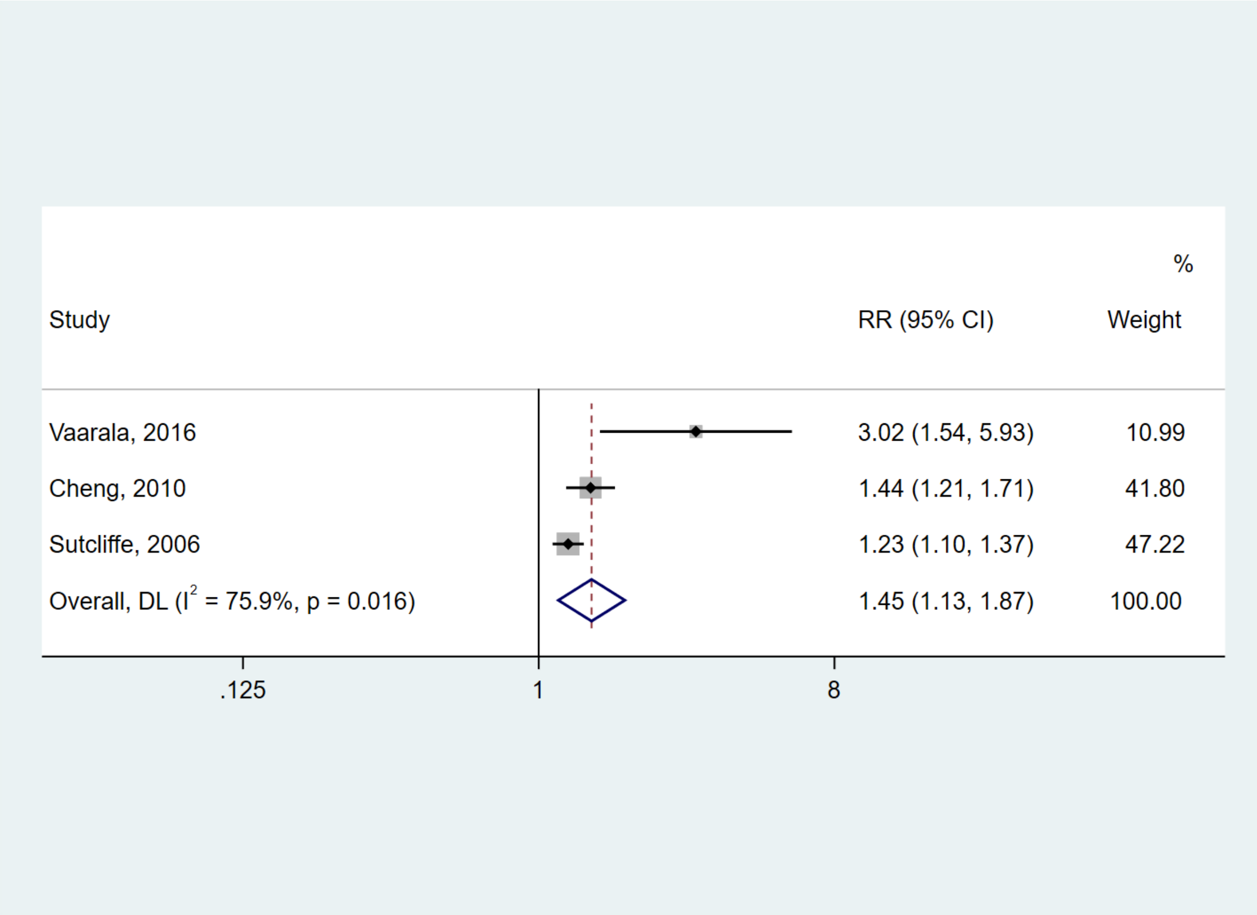

Category: Clinical variables, diseases, and treatments
Factor: Schizophrenia
Comparison: patients vs non-patients

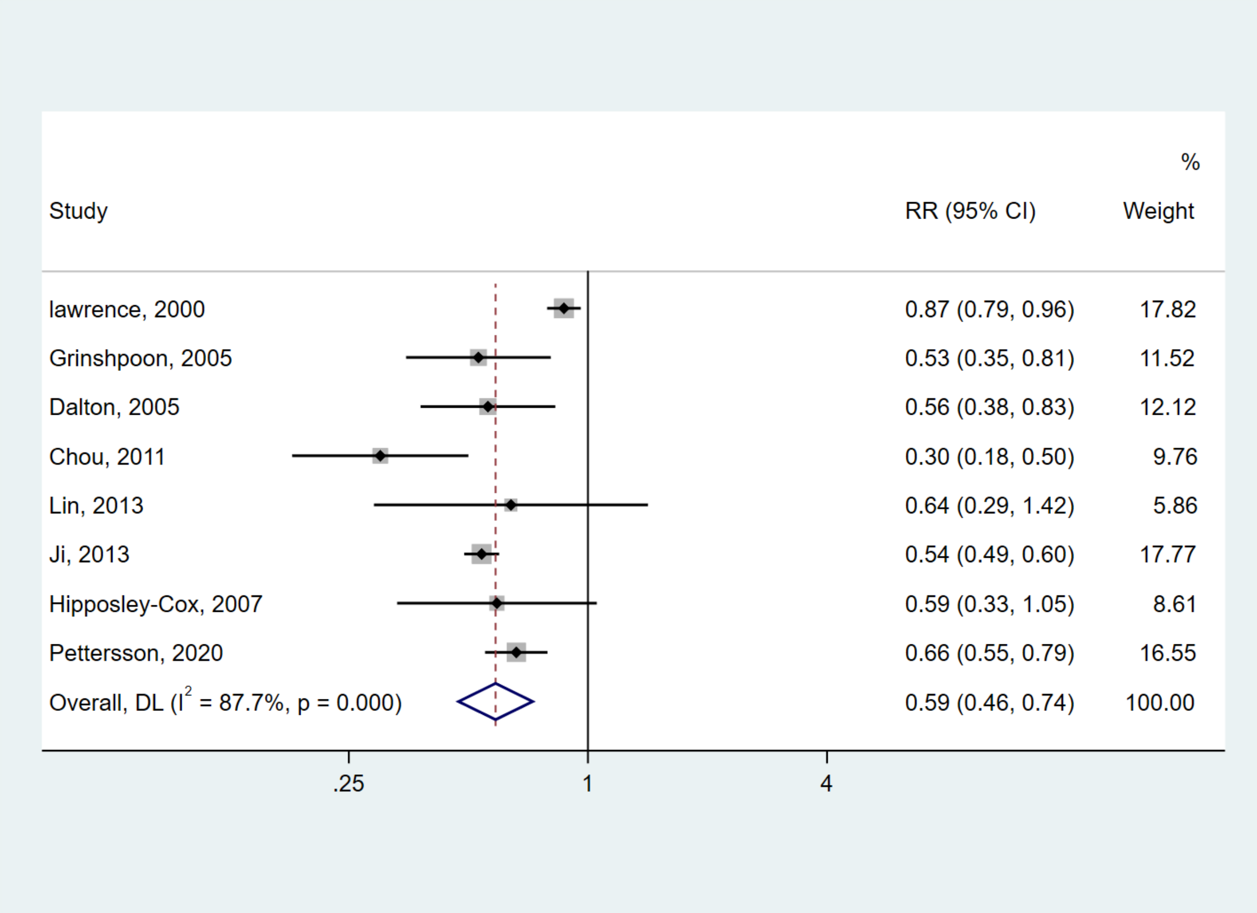

Category: Clinical variables, diseases, and treatments
Factor: Type 2 diabetes
Comparison: patients vs non-patients

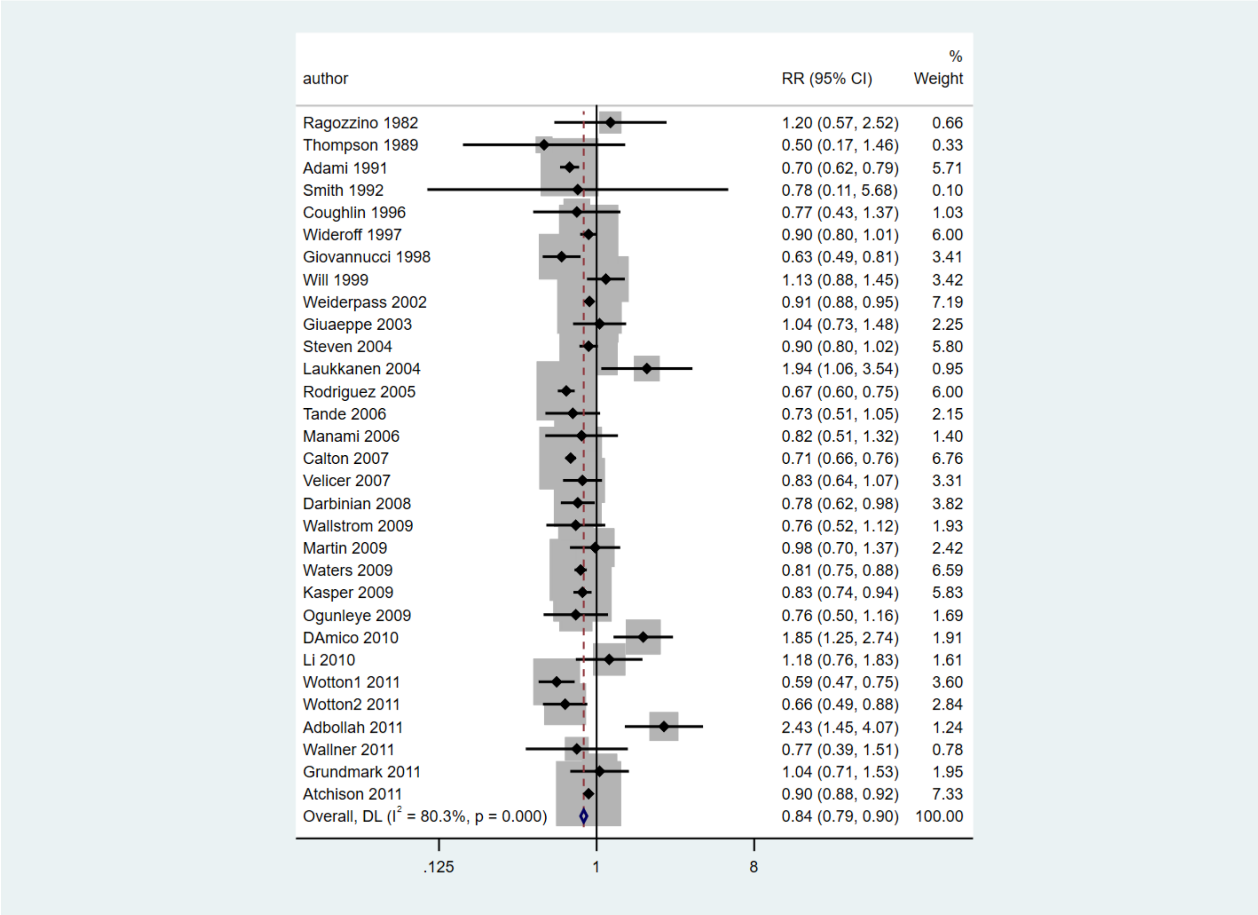

Category: Clinical variables, diseases, and treatments
Factor: Ulcerative colitis
Comparison: patients vs non-patients

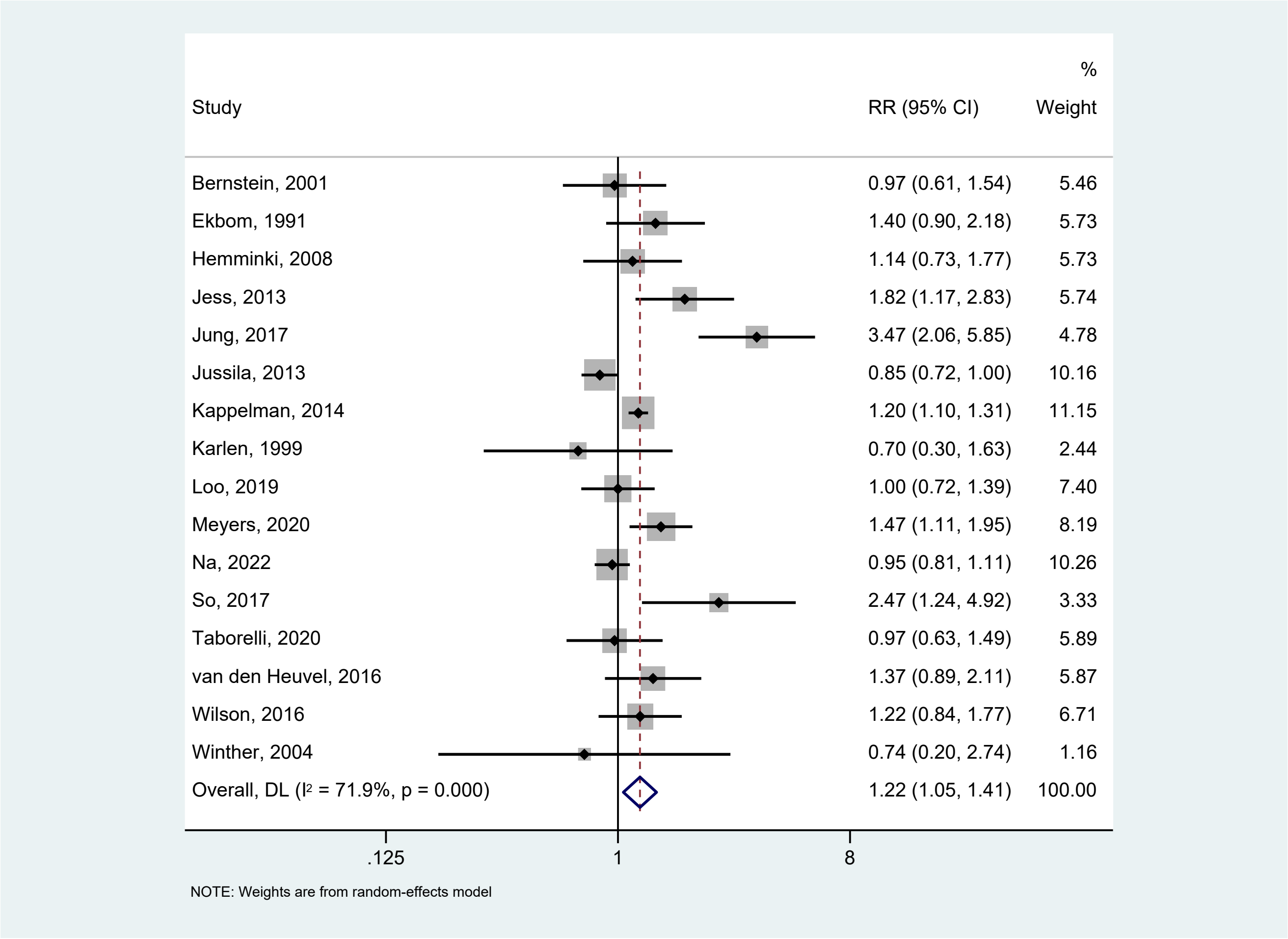

Category: Clinical variables, diseases, and treatments
Factor: Finasteride
Comparison: users vs non-users

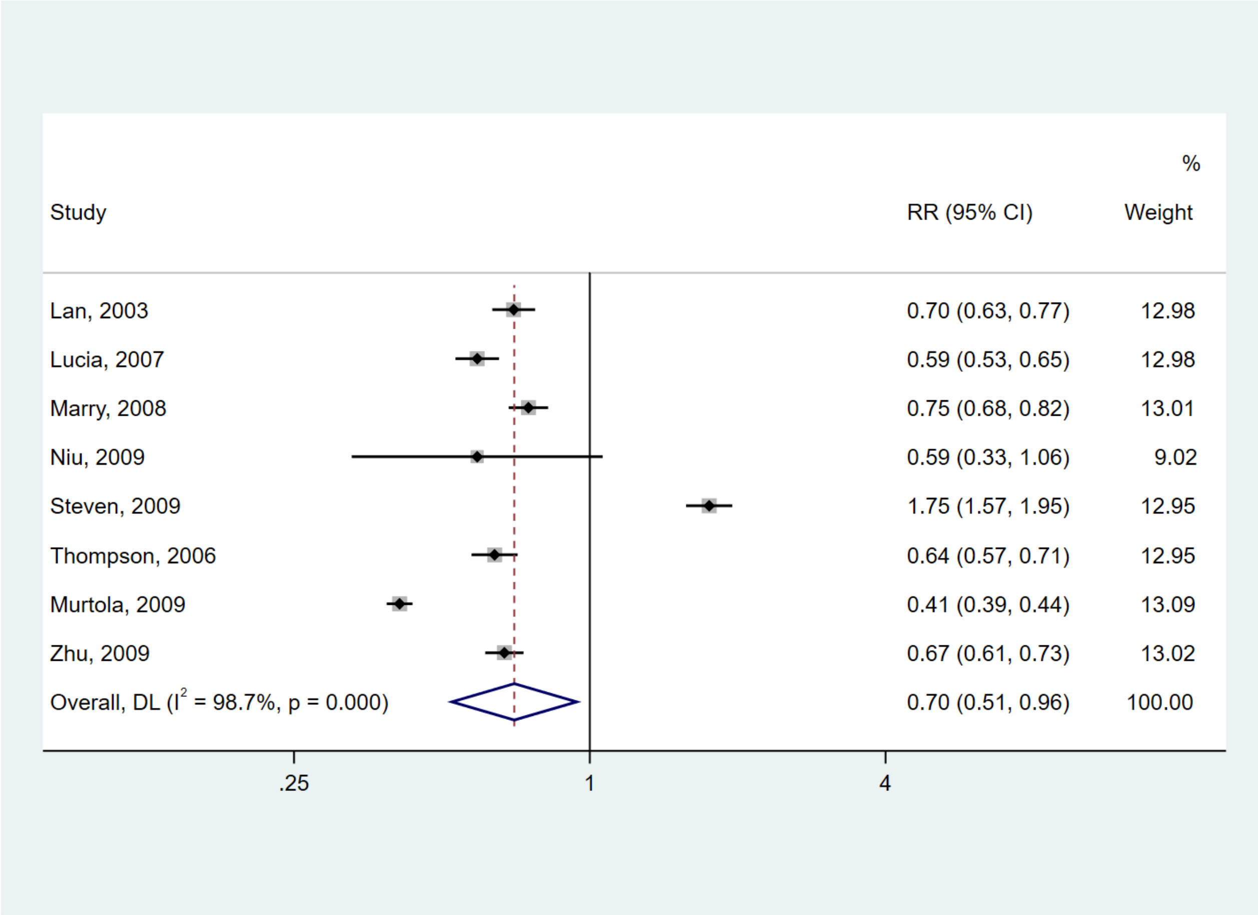

Category: Clinical variables, diseases, and treatments
Factor: Finasteride (for high grade prostate cancer)
Comparison: users vs non-users

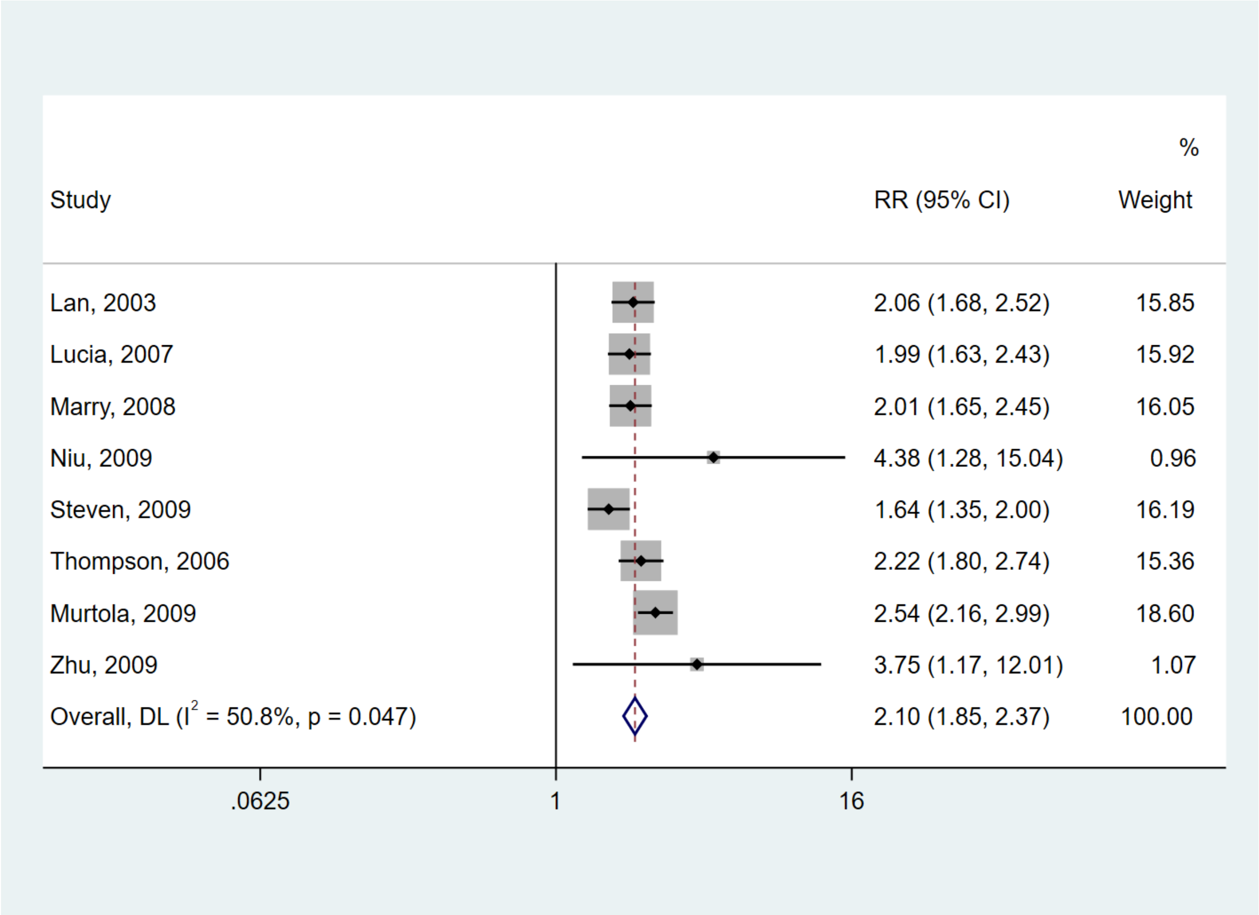

Category: Clinical variables, diseases, and treatments
Factor: Digoxin
Comparison: users vs non-users

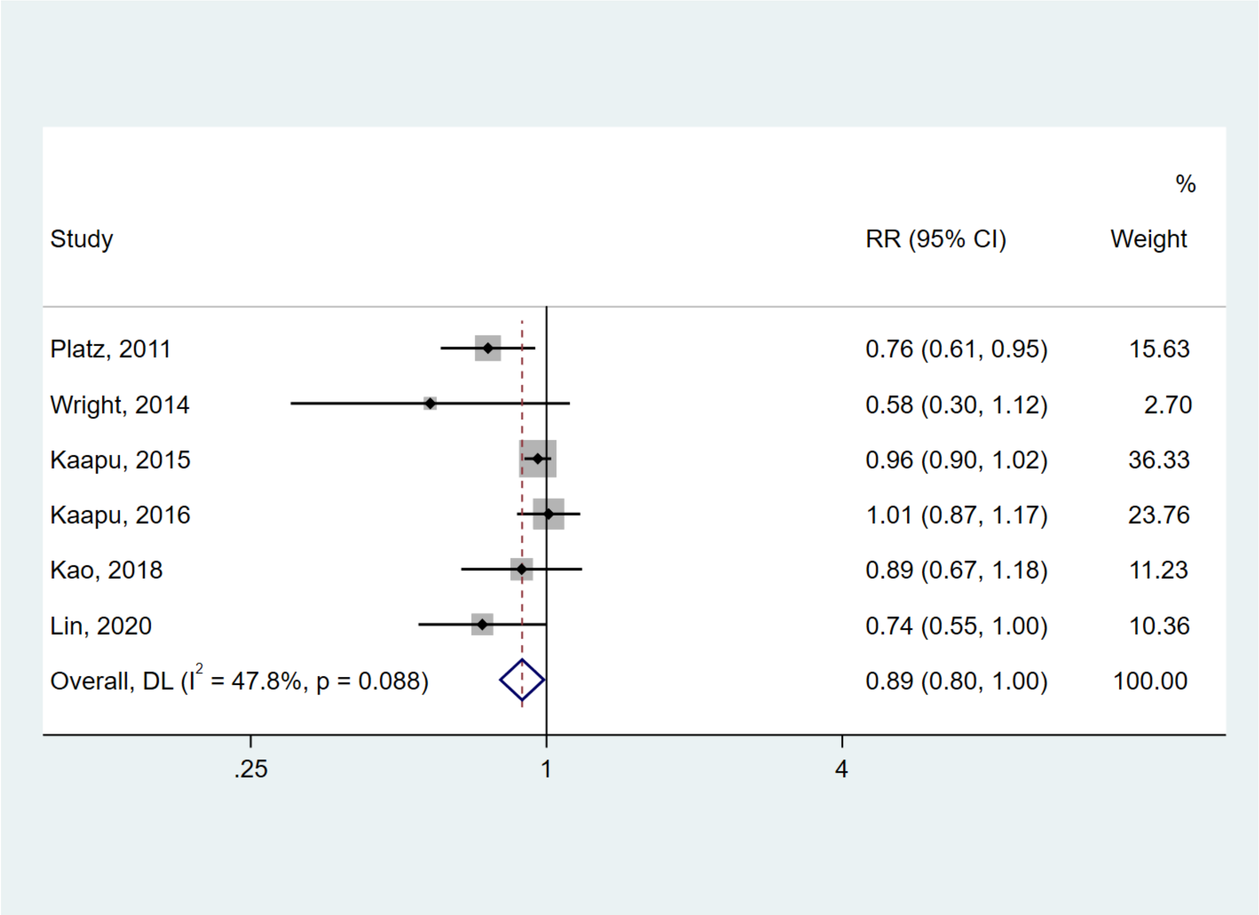

Category: Clinical variables, diseases, and treatments
Factor: Regular use of aspirin
Comparison: users vs non-users

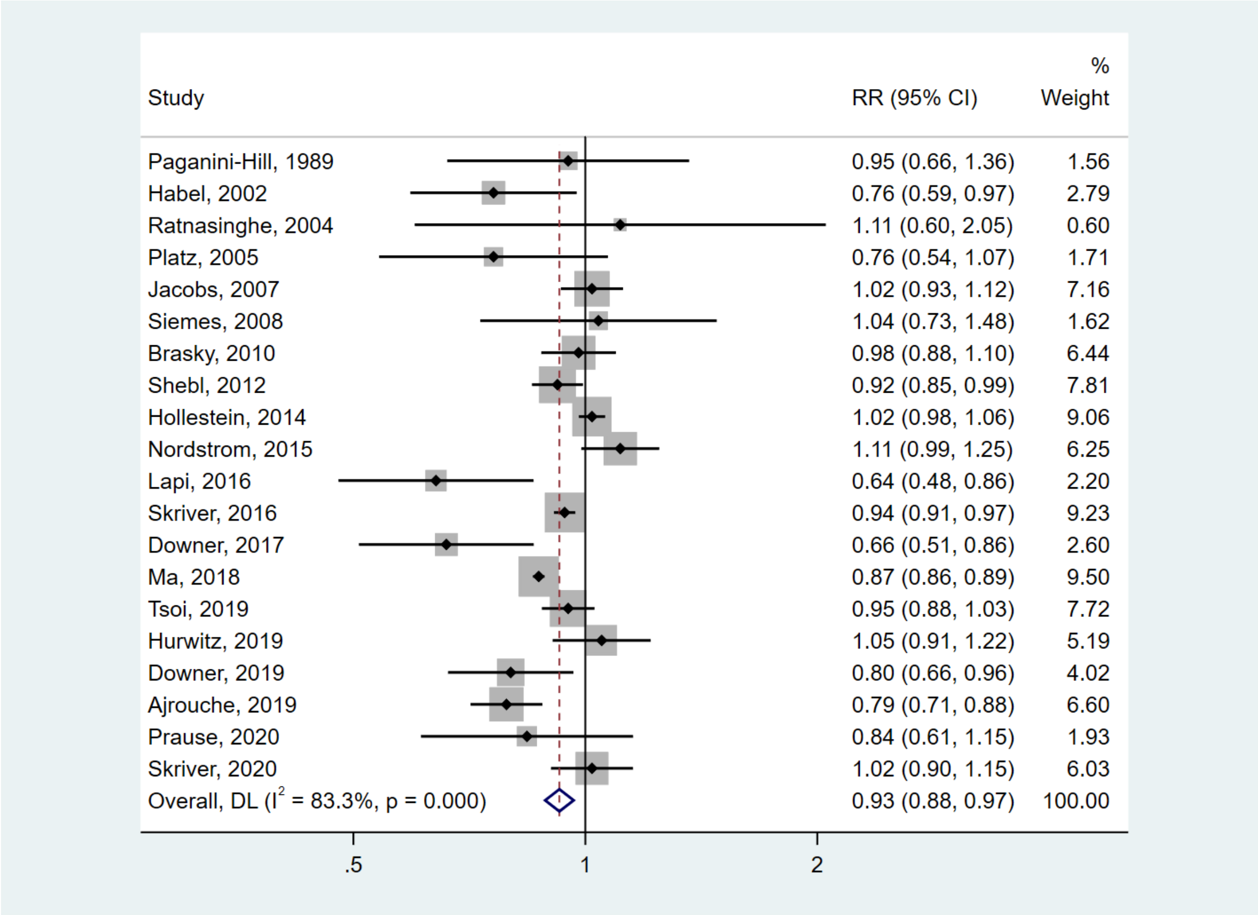

Category: Clinical variables, diseases, and treatments
Factor: Vasectomy
Comparison: treated vs non-treated

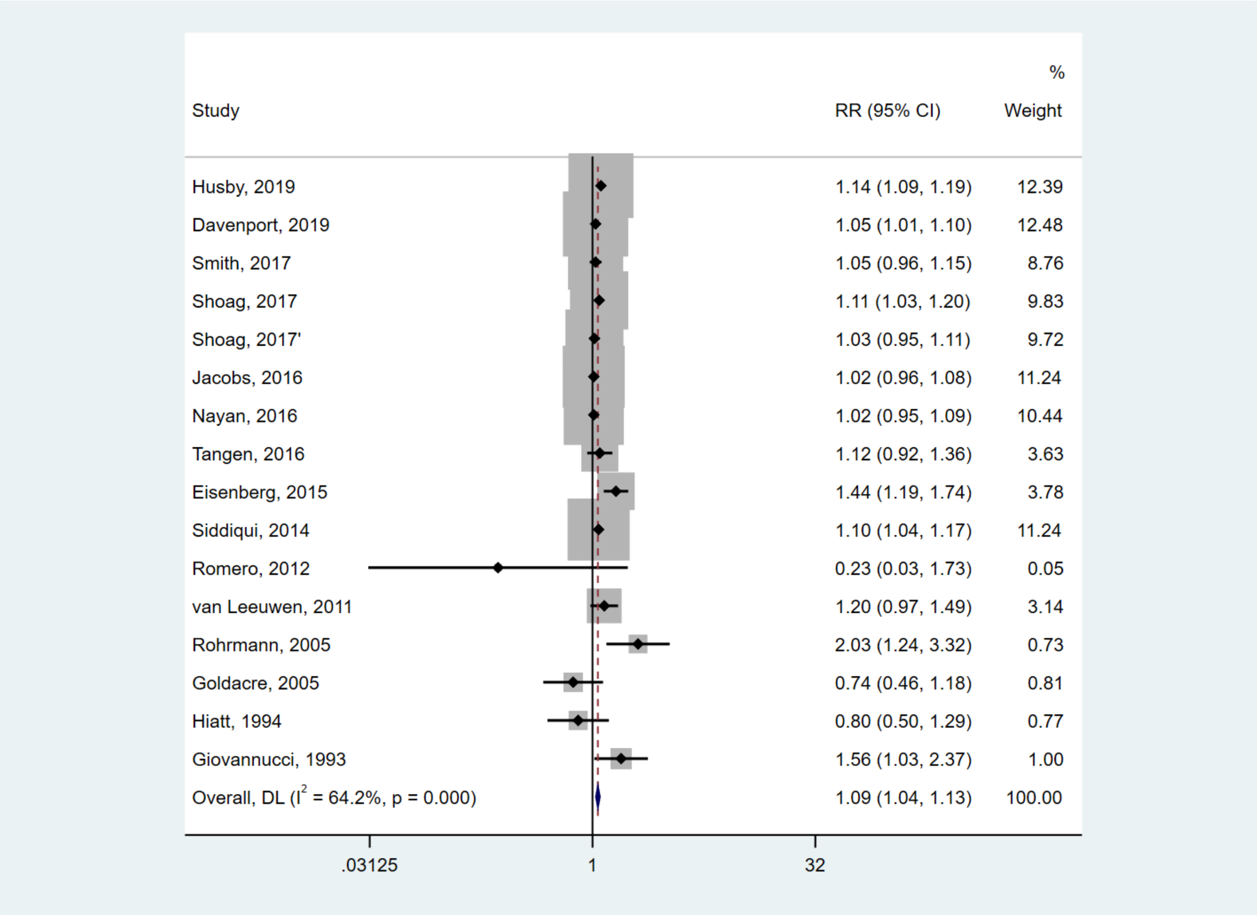

Category: Anthropometric indices
Factor: Adult weight gain
Comparison: highest vs lowest

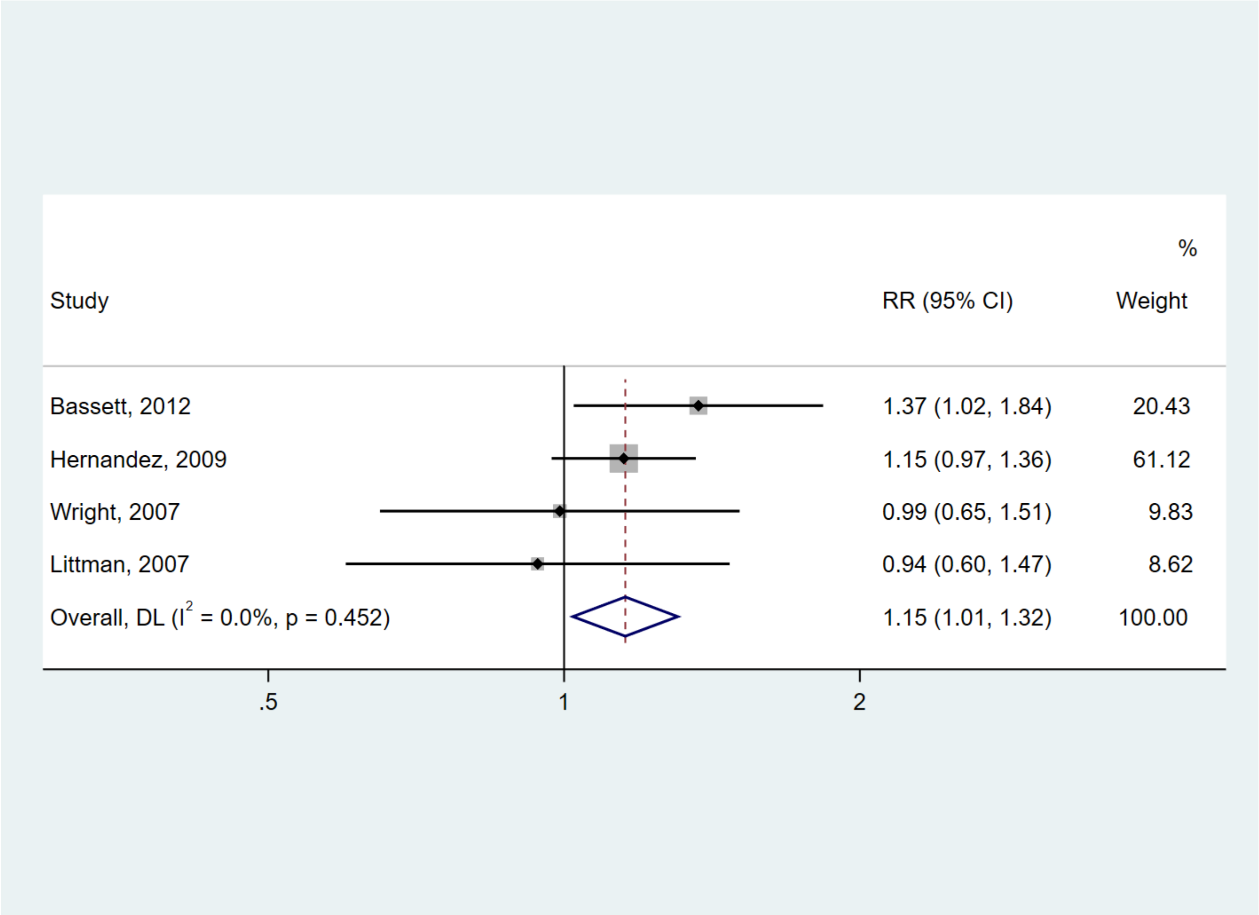

Category: Anthropometric indices
Factor: Birthweight
Comparison: per kg increase

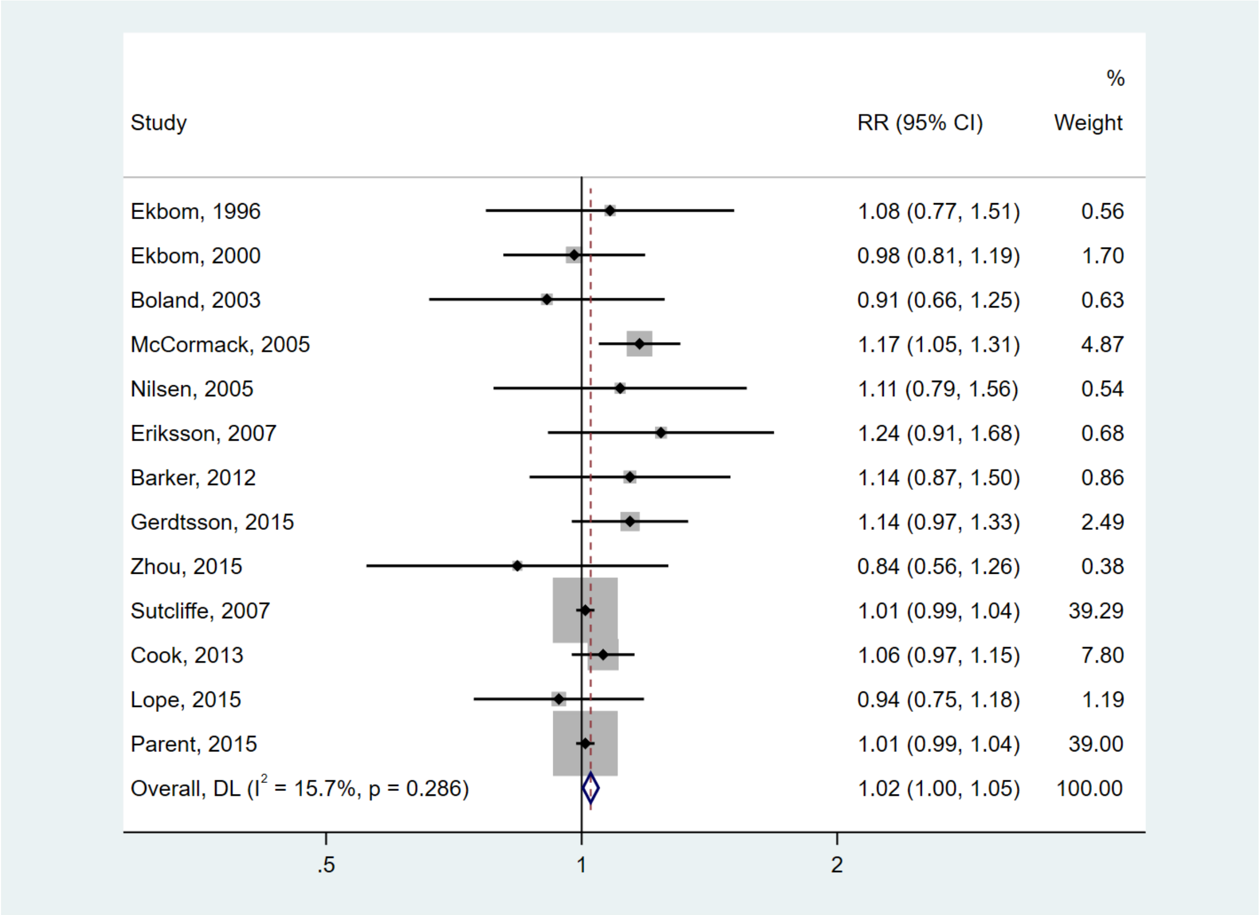

Category: Anthropometric indices
Factor: Fat mass
Comparison: highest vs lowest

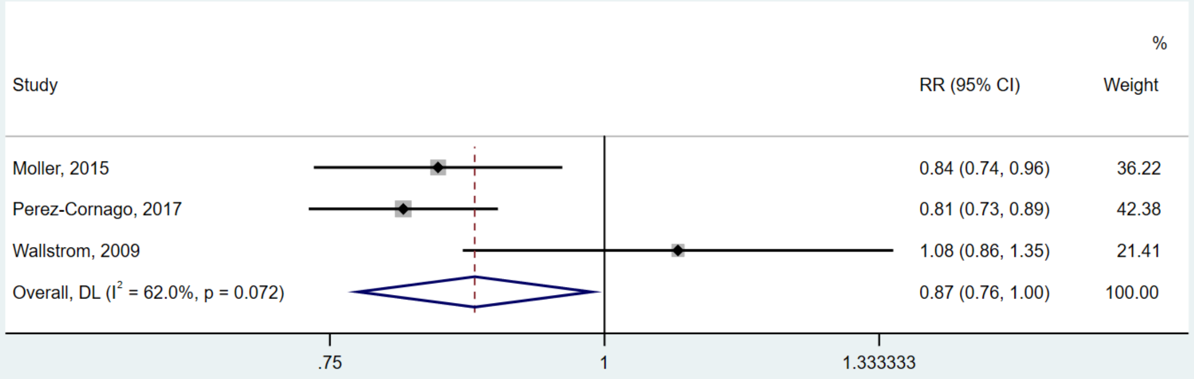

Category: Anthropometric indices
Factor: Height
Comparison: per 10 cm increase

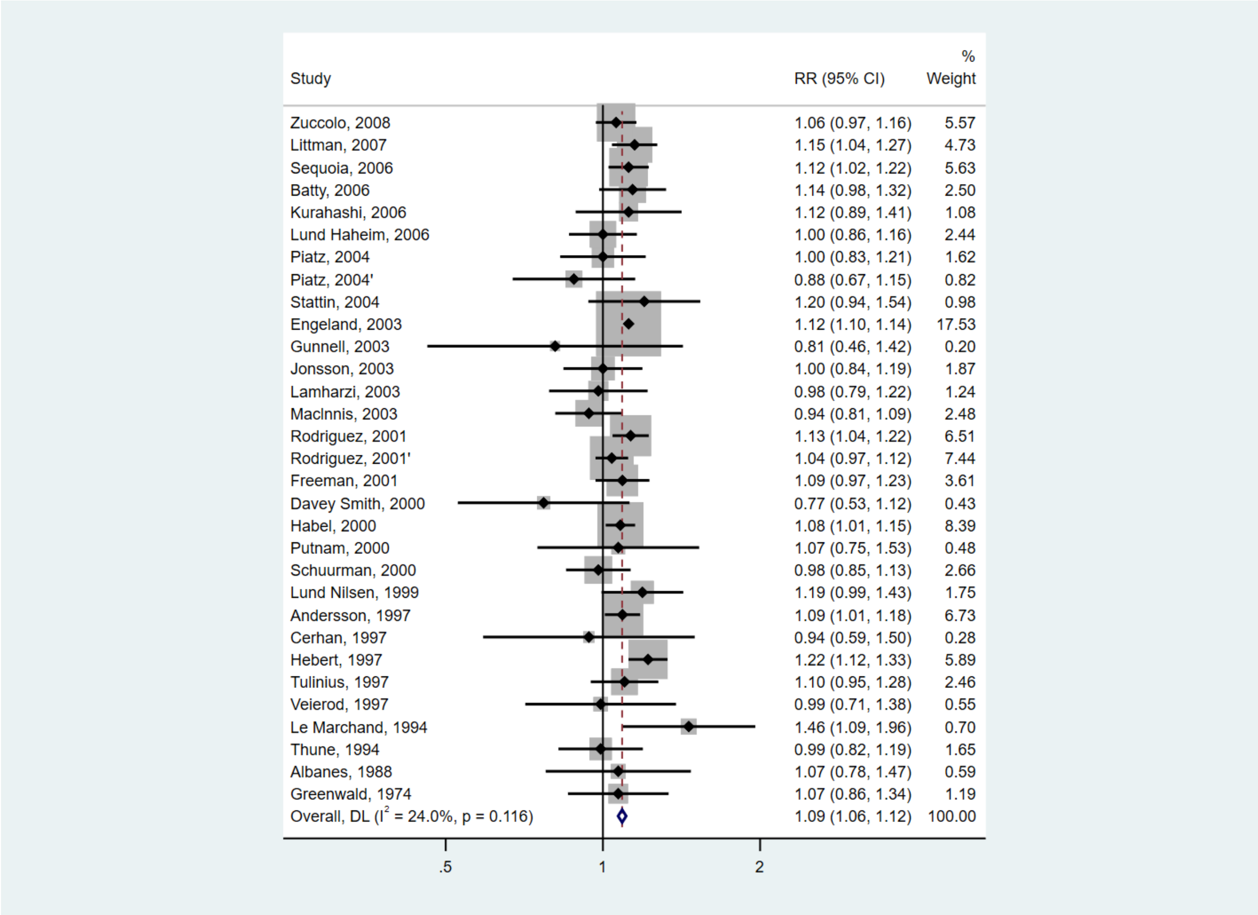

Category: Lifestyle
Factor: Coffee
Comparison: highest vs lowest

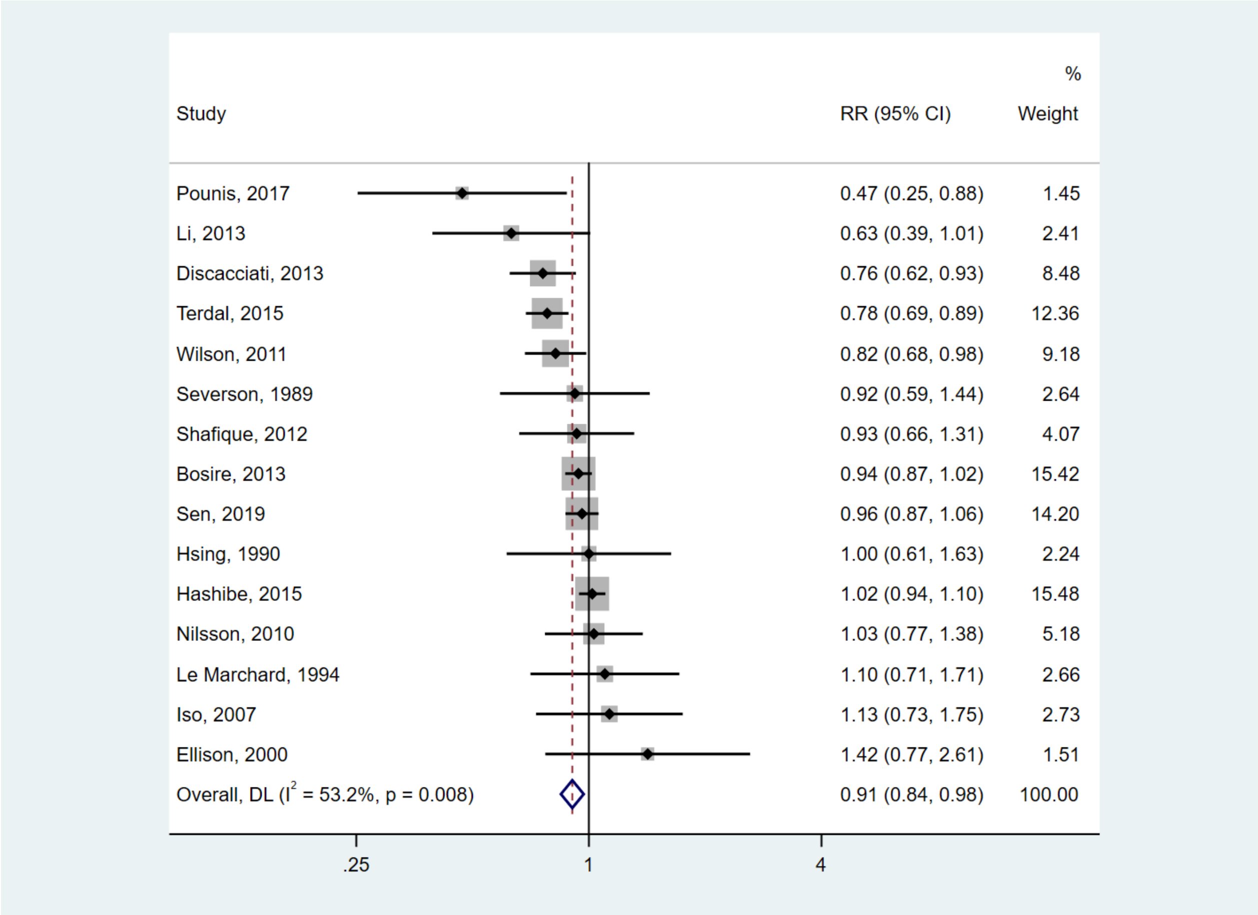

Category: Lifestyle
Factor: Current smoking
Comparison: current smoking vs non-smoker

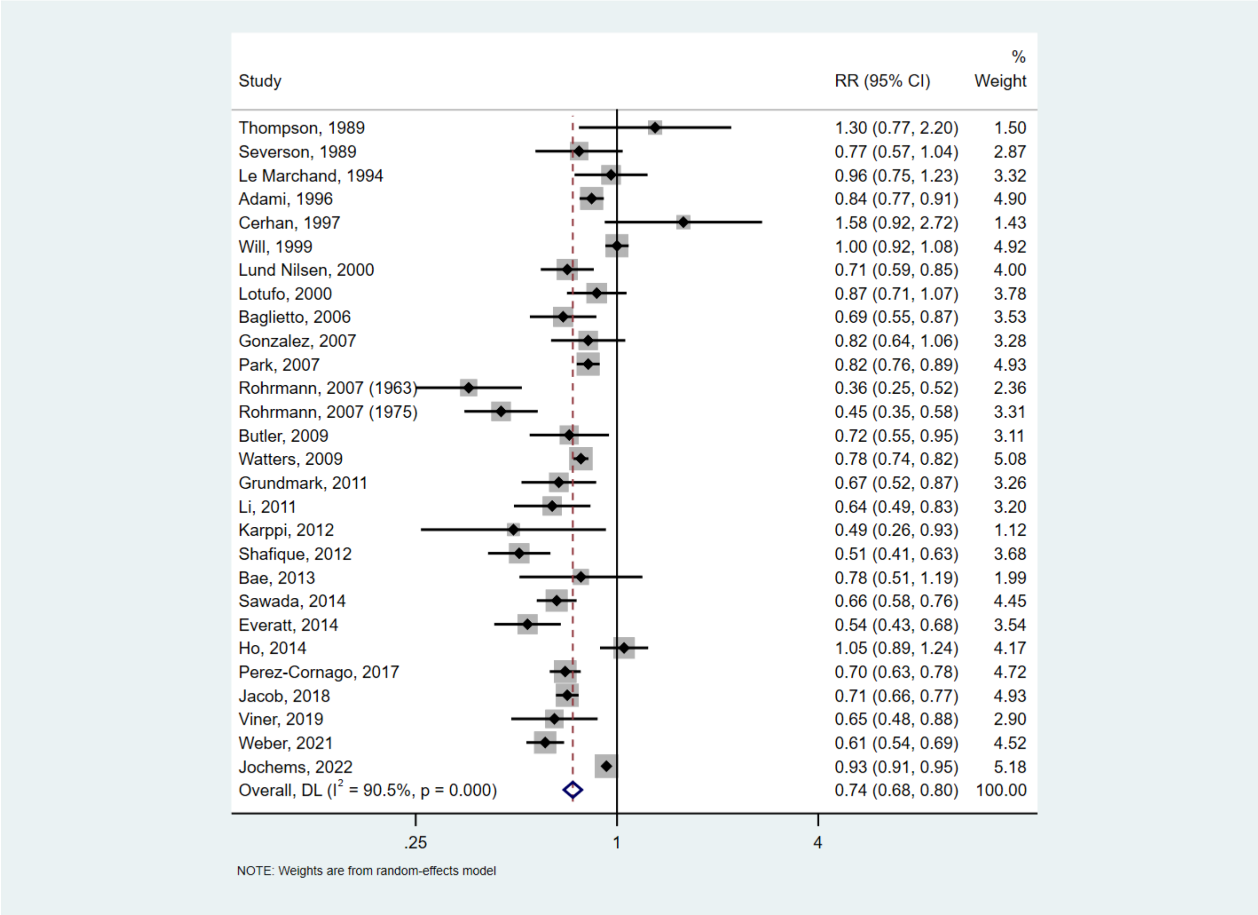

Category: Lifestyle
Factor: Age at first intercourse
Comparison: highest vs lowest

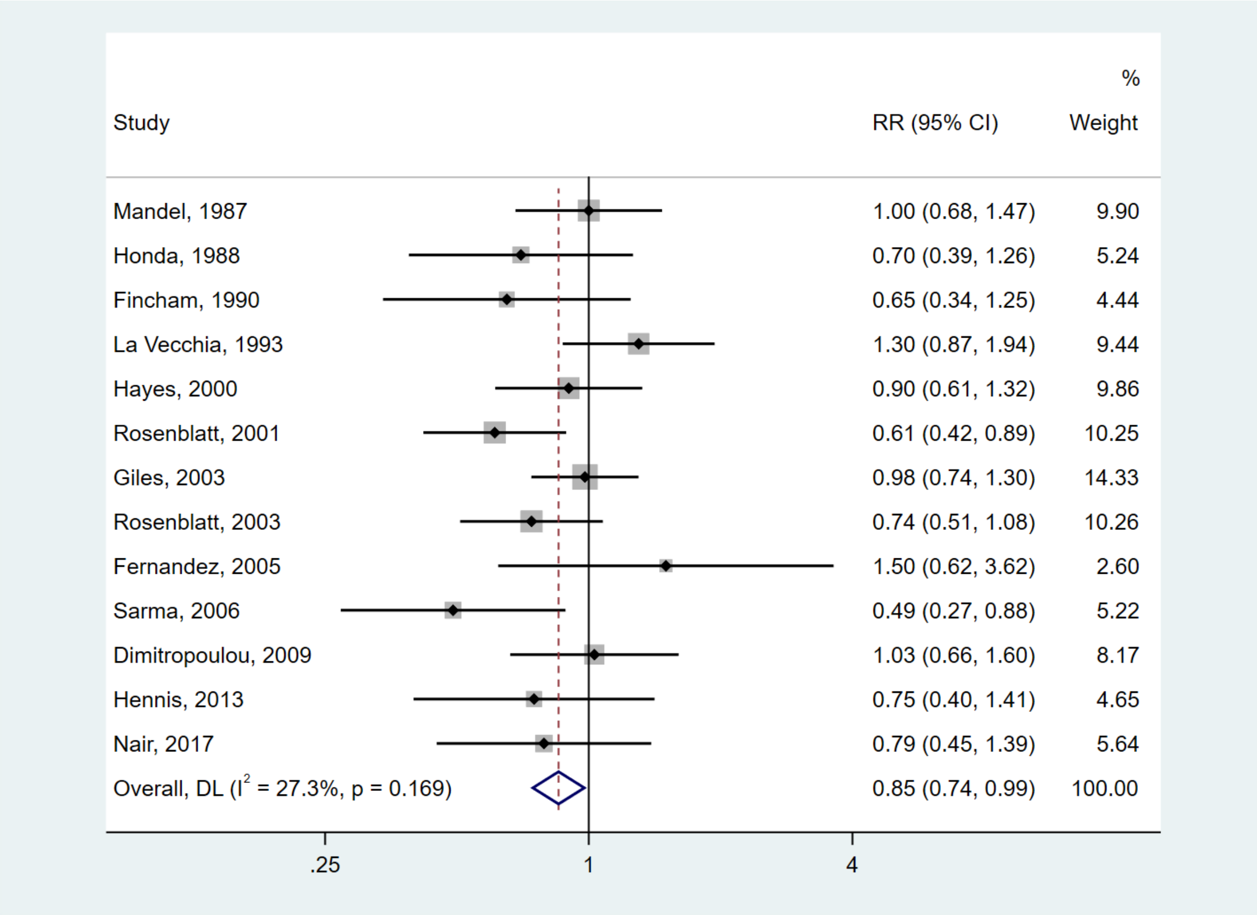

Category: Lifestyle
Factor: Firefighter
Comparison: ever-employment as a career firefighter vs general population

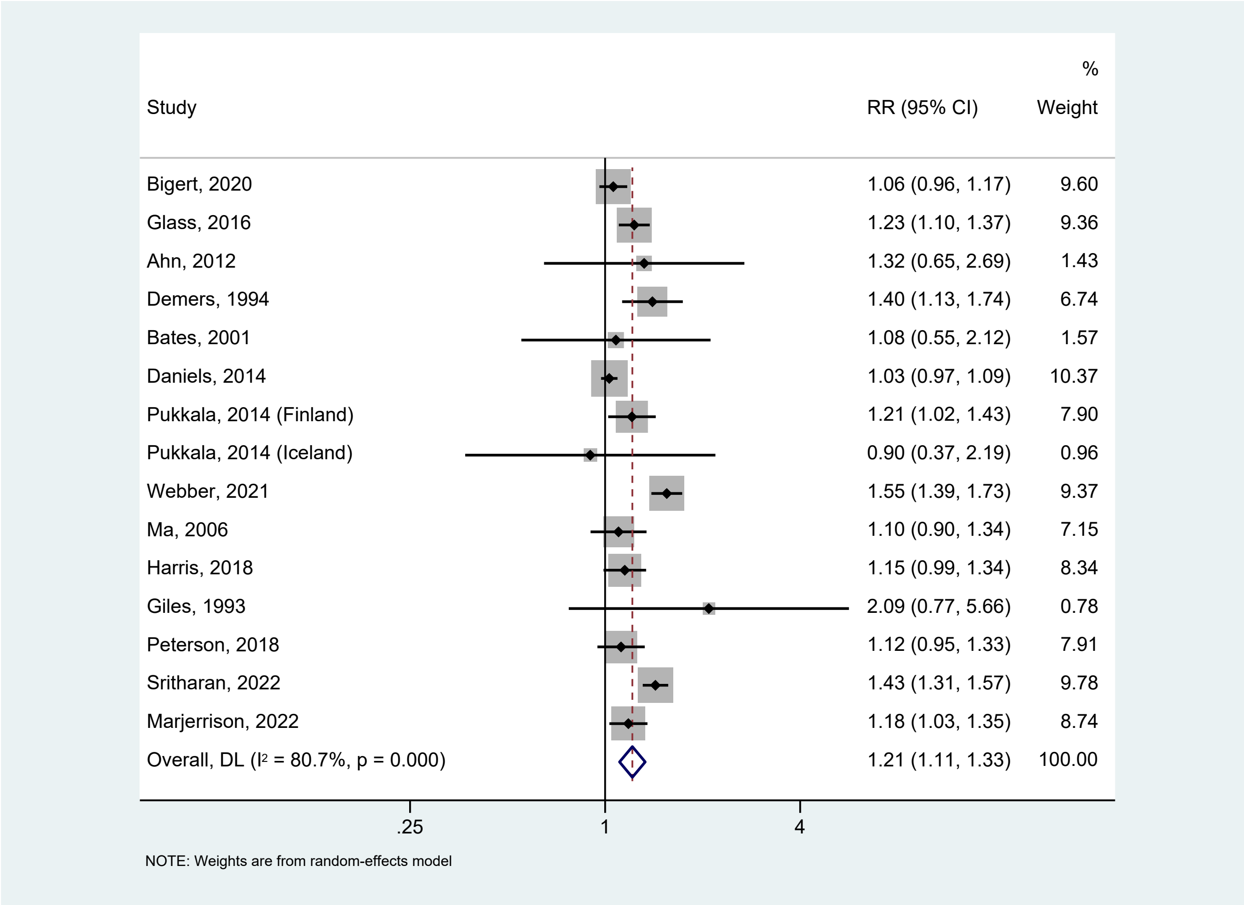

Category: Lifestyle
Factor: Number of female sexual partners
Comparison: highest vs lowest

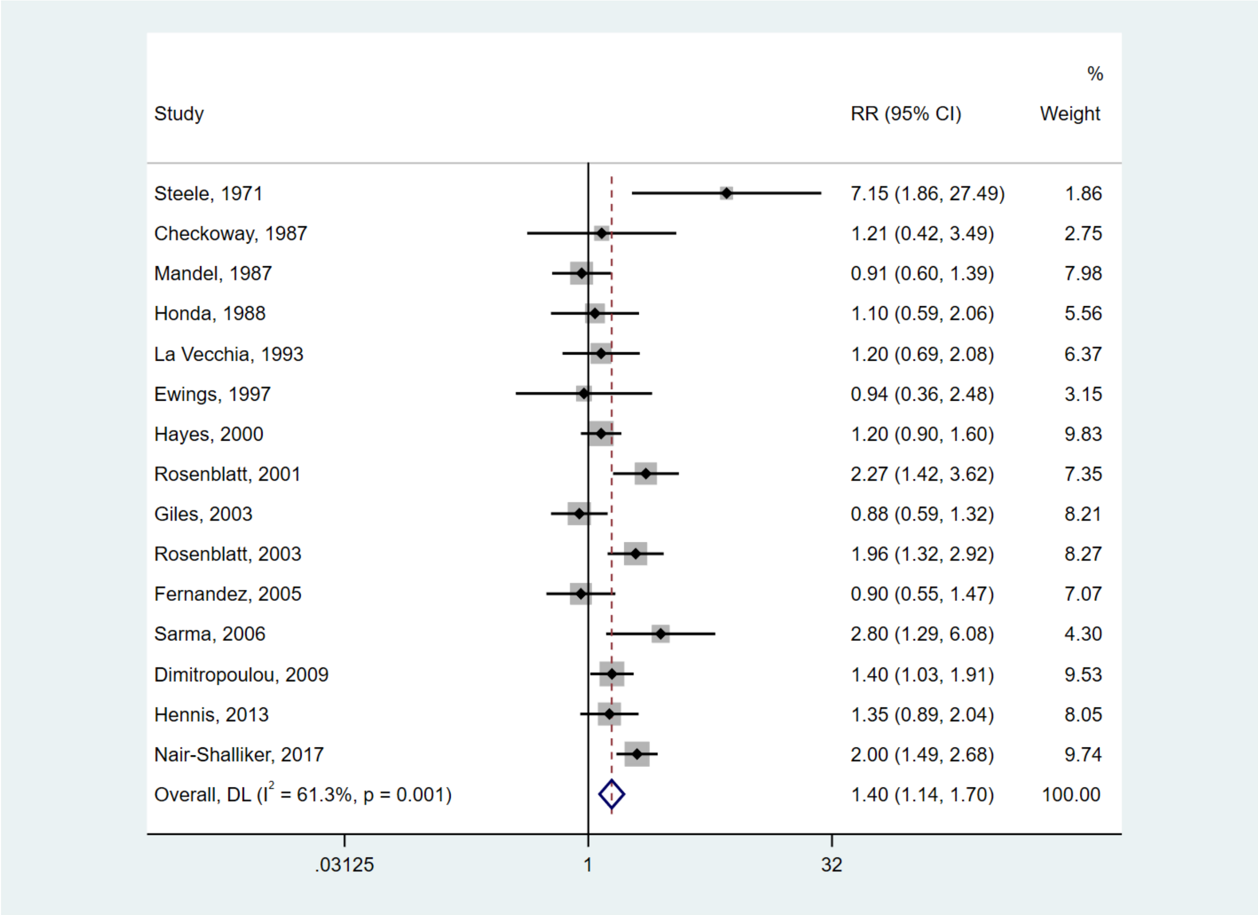

Category: Lifestyle
Factor: Occupational physical activity
Comparison: higher vs lower

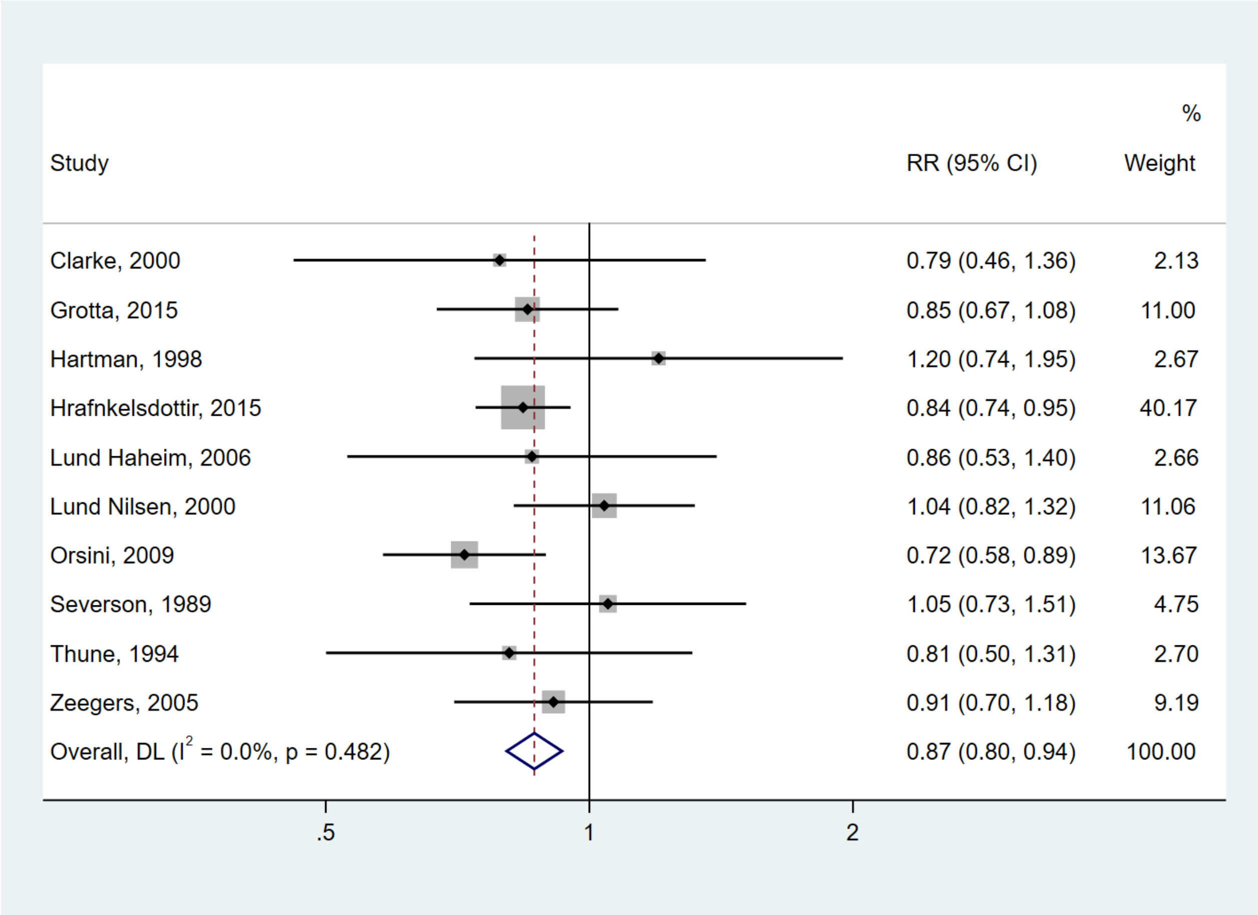

Category: Biomarker
Factor: Blood α-tocopherol level
Comparison: highest vs lowest

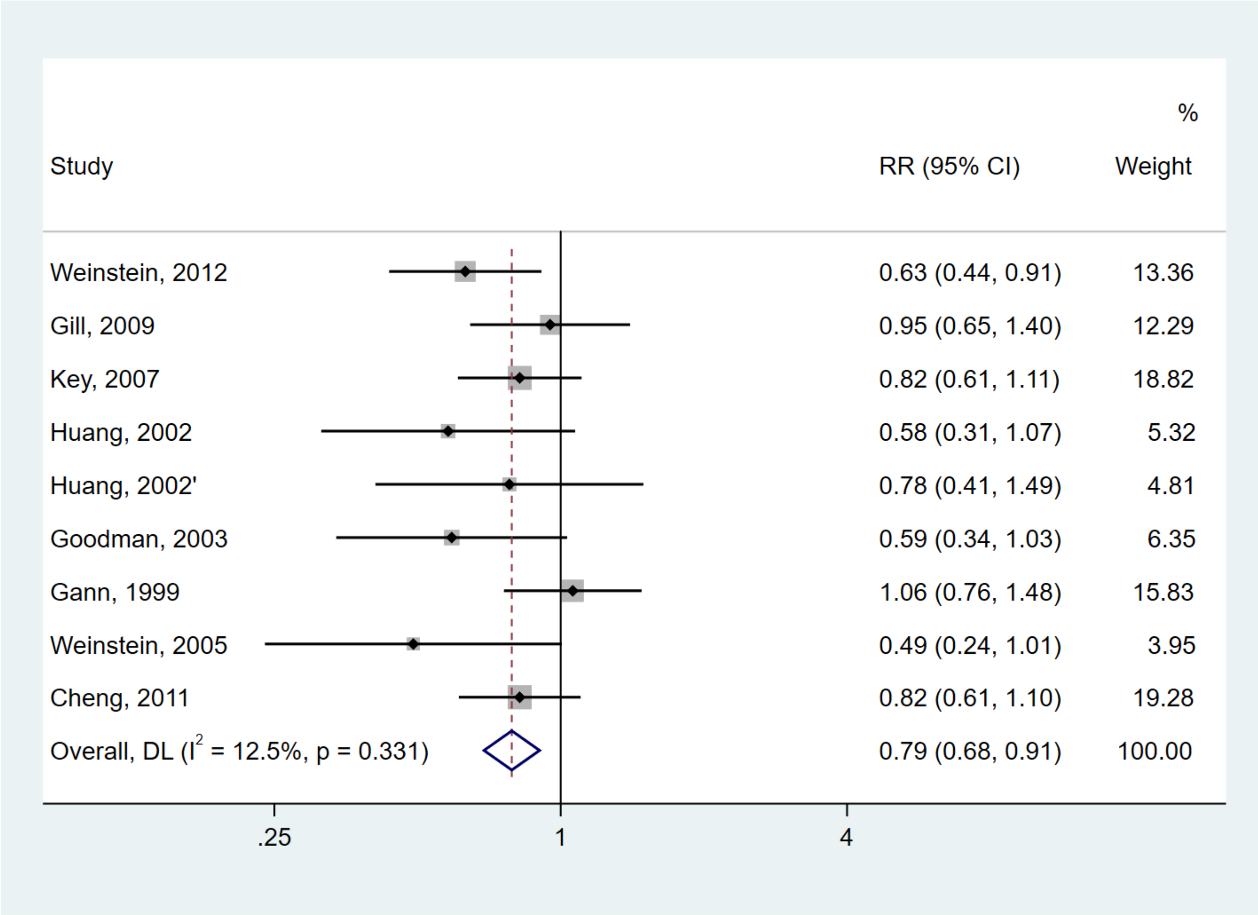

Category: Biomarker
Factor: C-reactive protein
Comparison: highest vs lowest

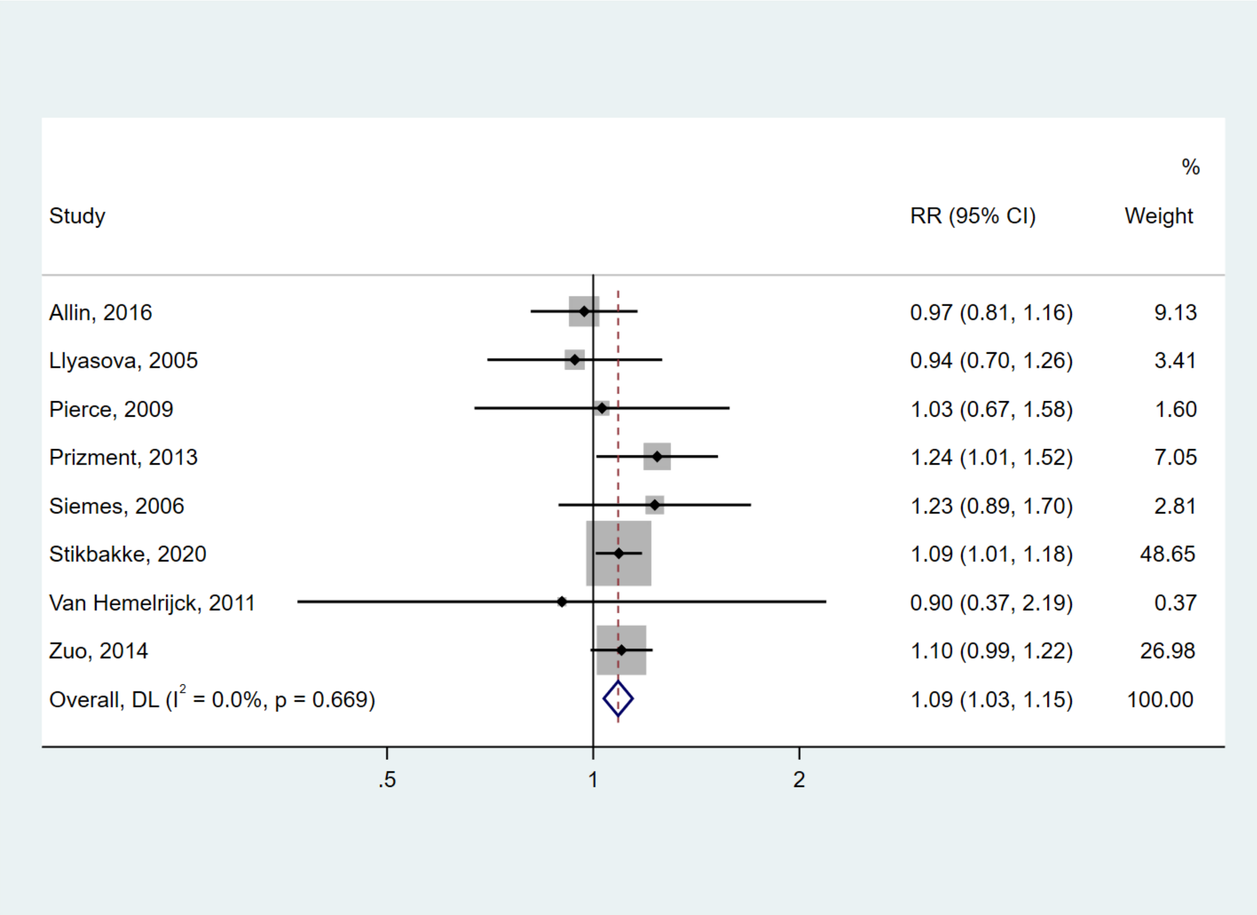

Category: Biomarker
Factor: Tissue level linoleic acid
Comparison: highest vs lowest

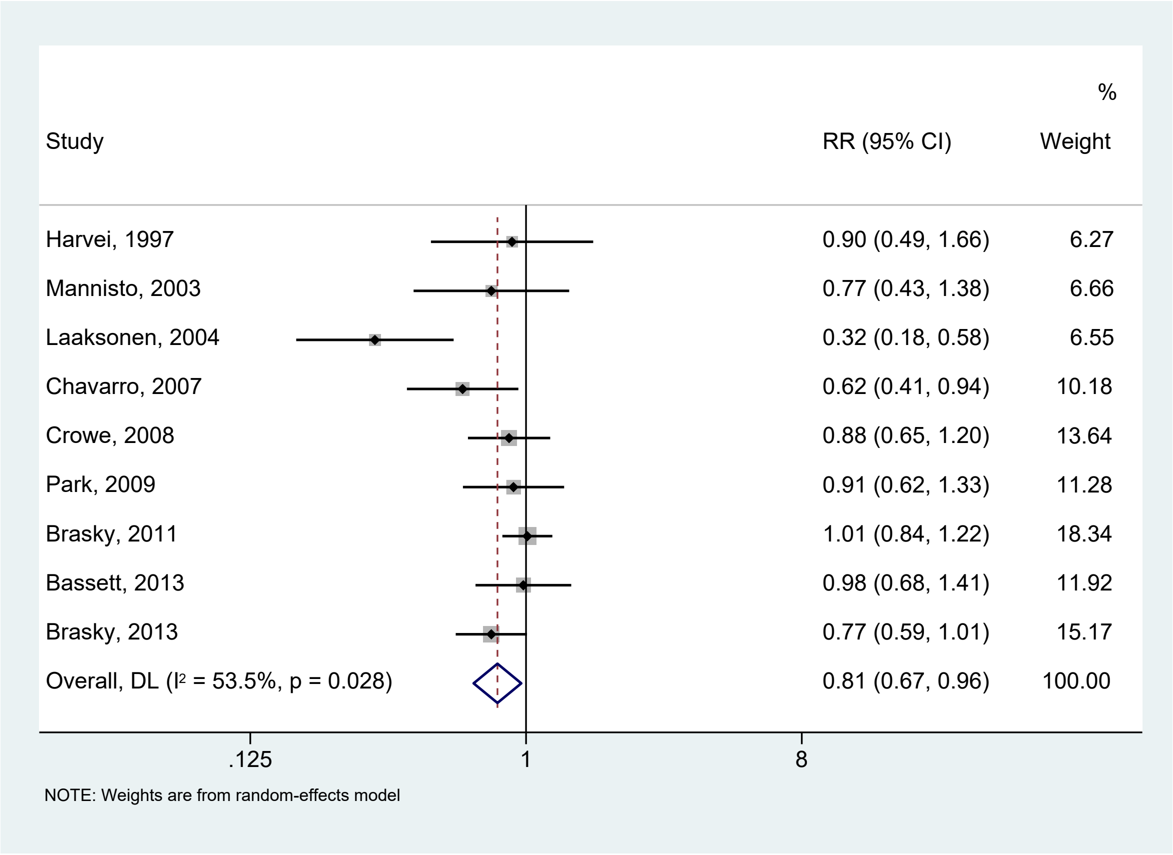

Category: Biomarker
Factor: Total cholesterol
Comparison: highest vs lowest

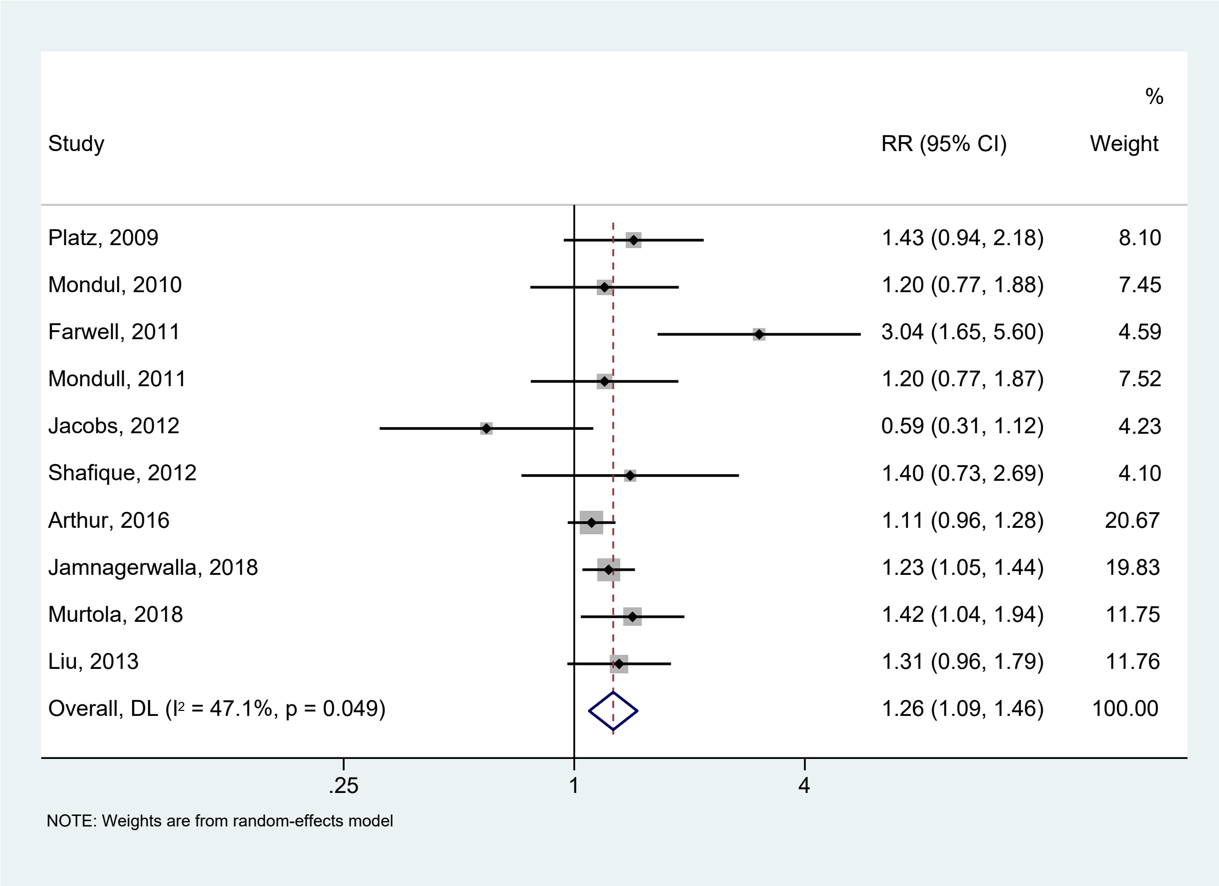

Category: Biomarker
Factor: Serum folate
Comparison: highest vs lowest

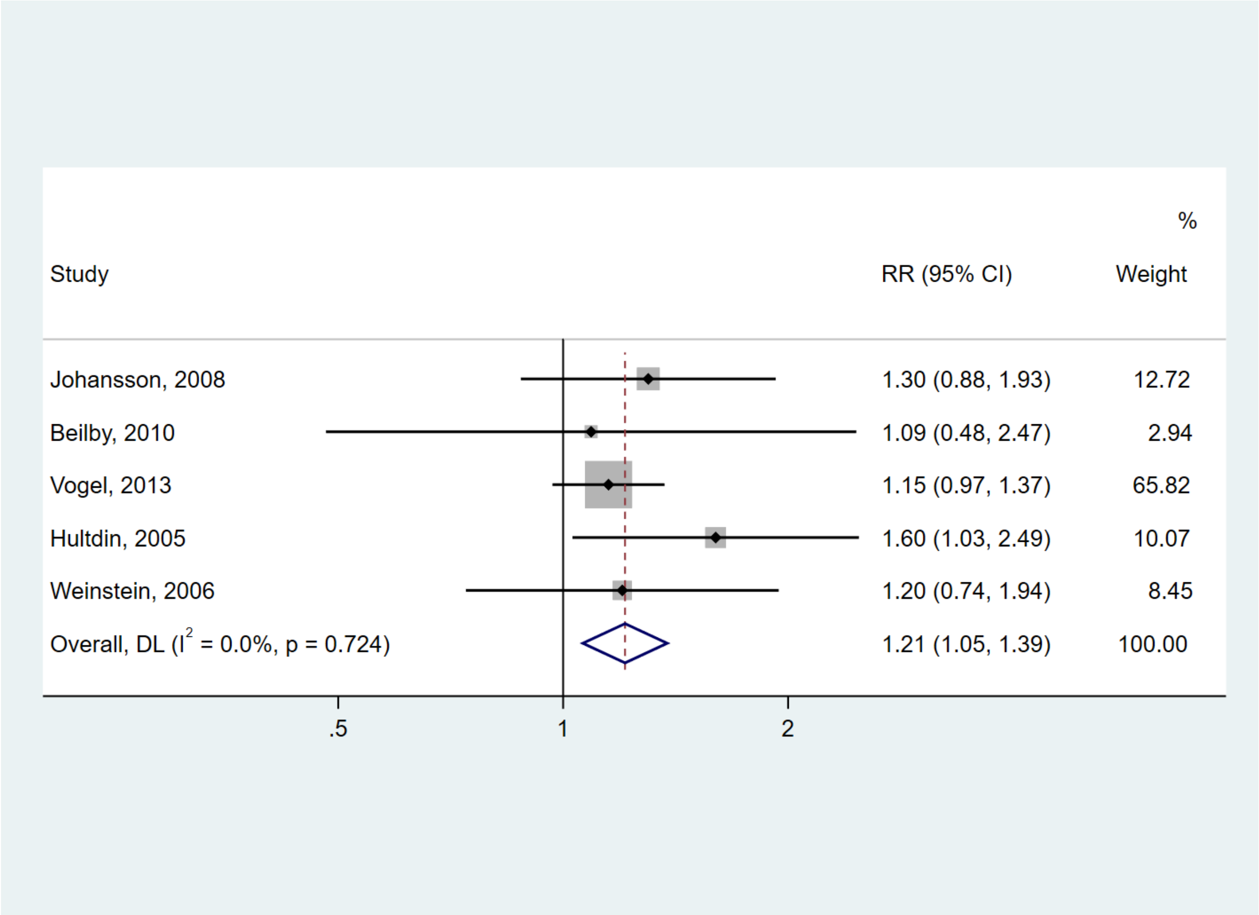

Category: Diet and nutrition
Factor: Selenium
Comparison: not reported

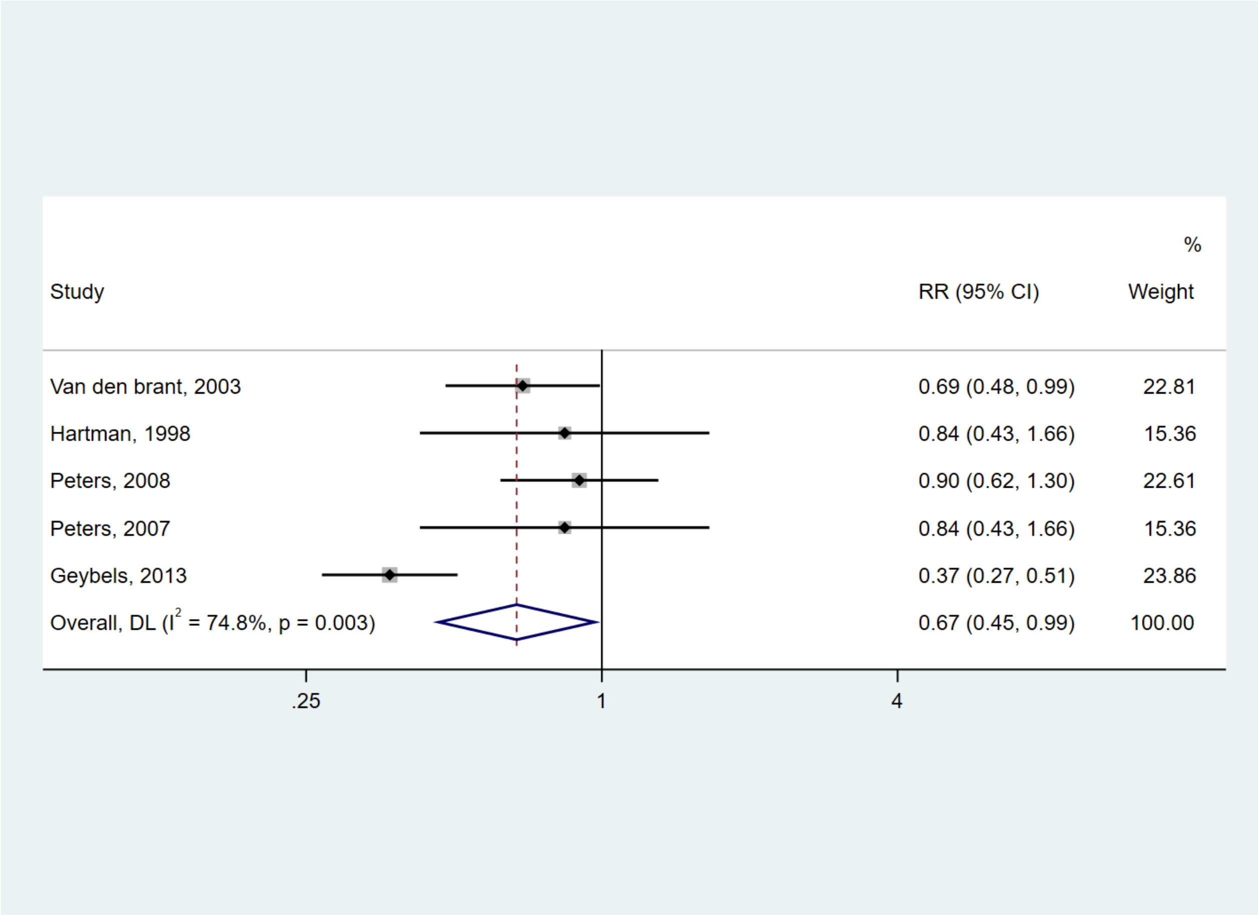

Category: Diet and nutrition
Factor: Total flavonoids
Comparison: highest vs lowest

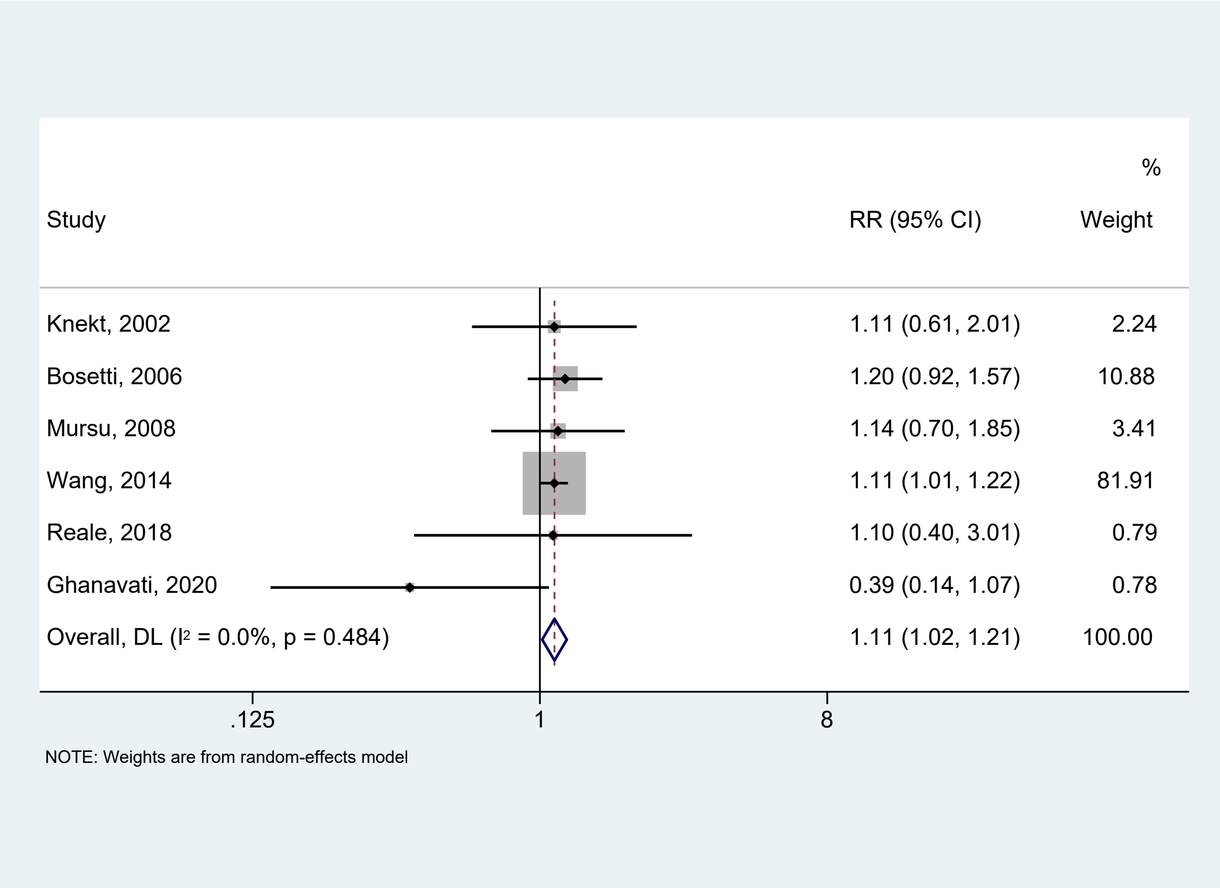

Category: Diet and nutrition
Factor: Circulating 25-hydroxyvitamin D
Comparison: highest vs lowest

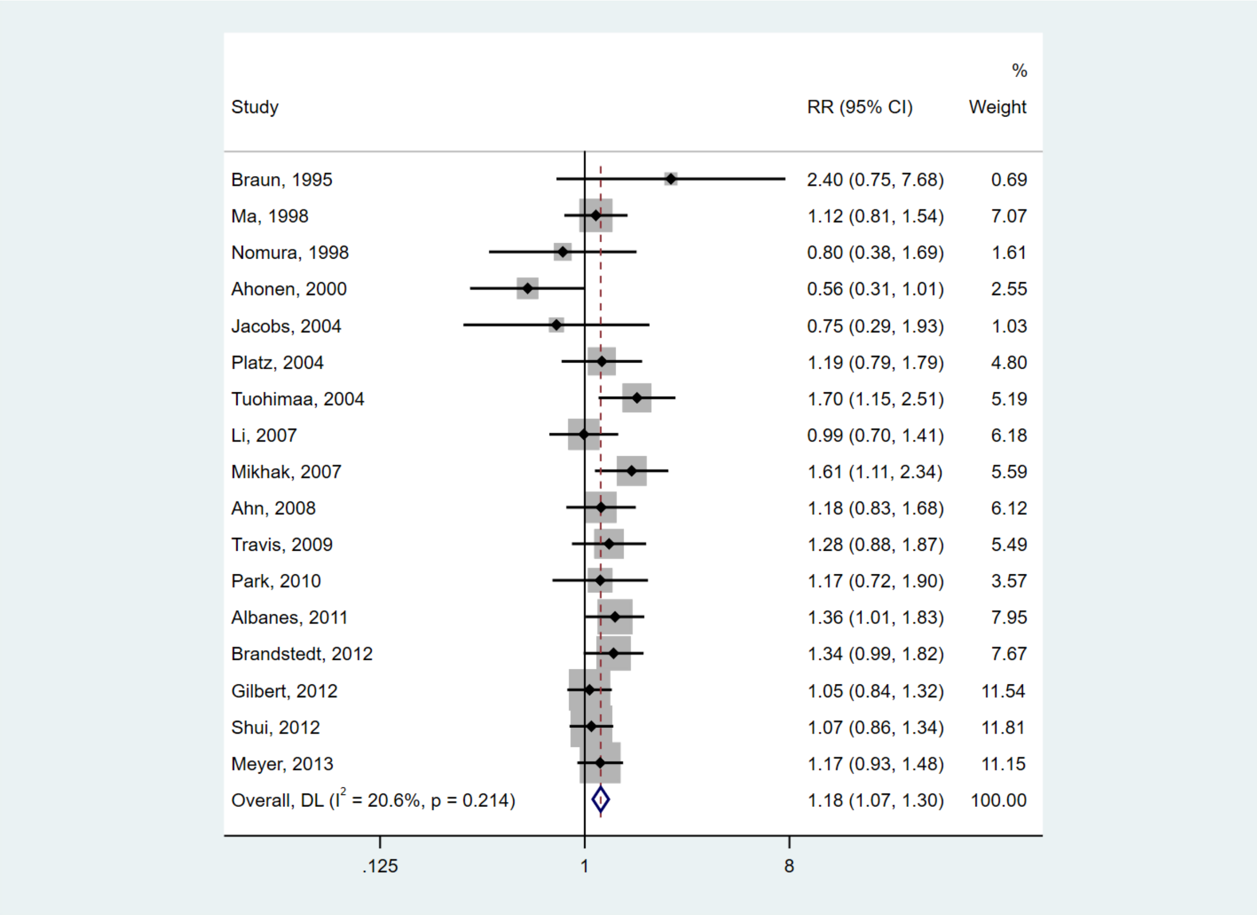

Category: Diet and nutrition
Factor: Daidzein
Comparison: highest vs lowest

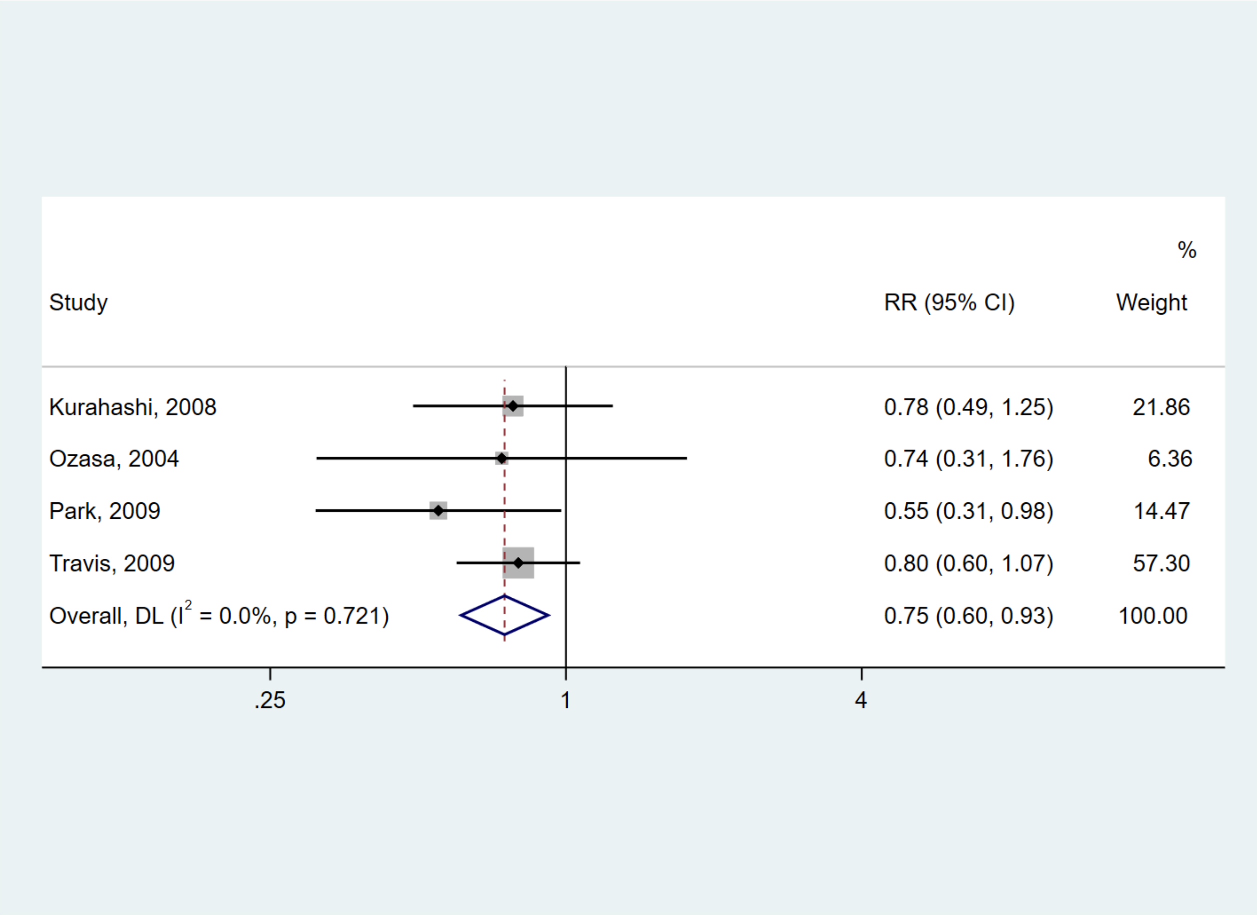

Category: Diet and nutrition
Factor: Total calcium intake
Comparison: per 400mg/d

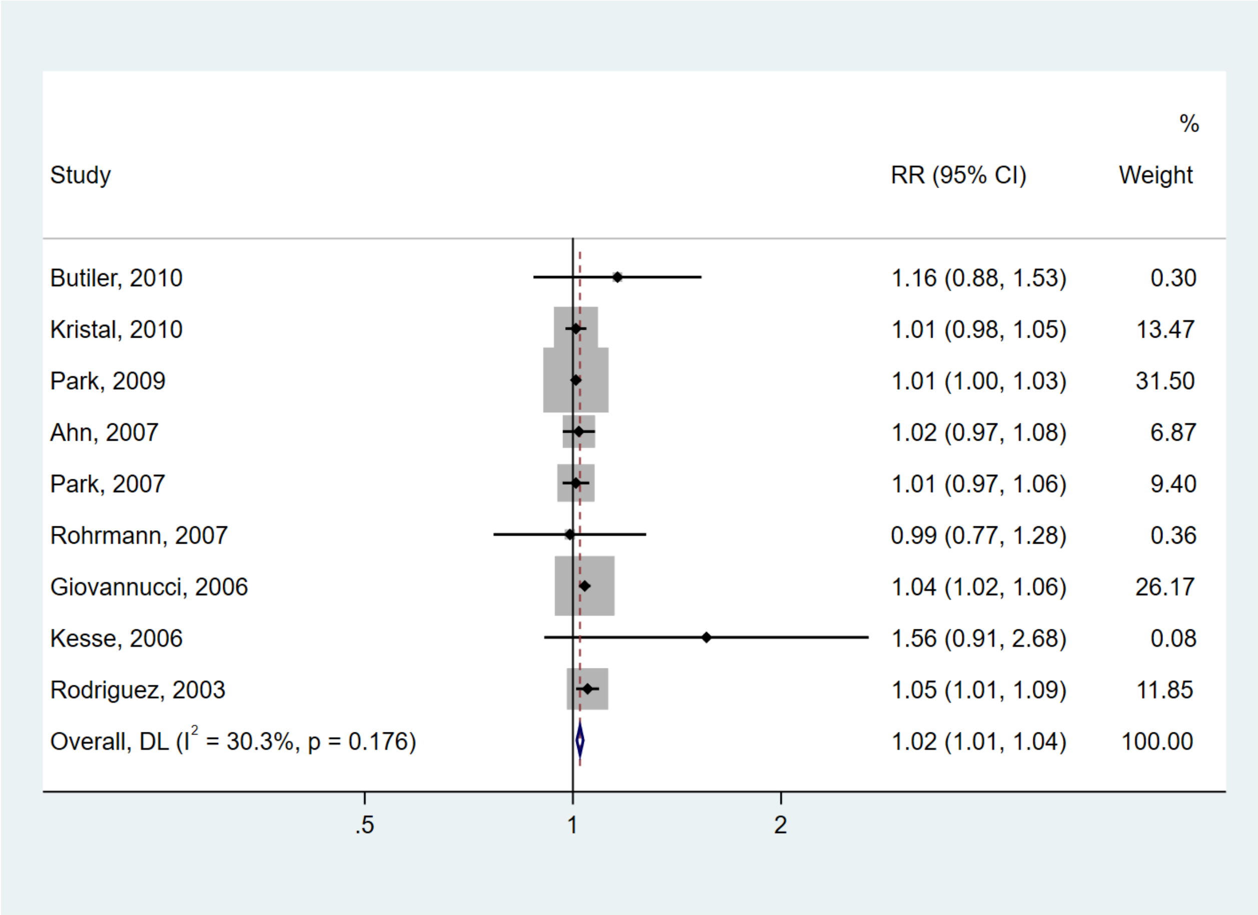

Category: Diet and nutrition
Factor: Total dairy products
Comparison: highest vs lowest

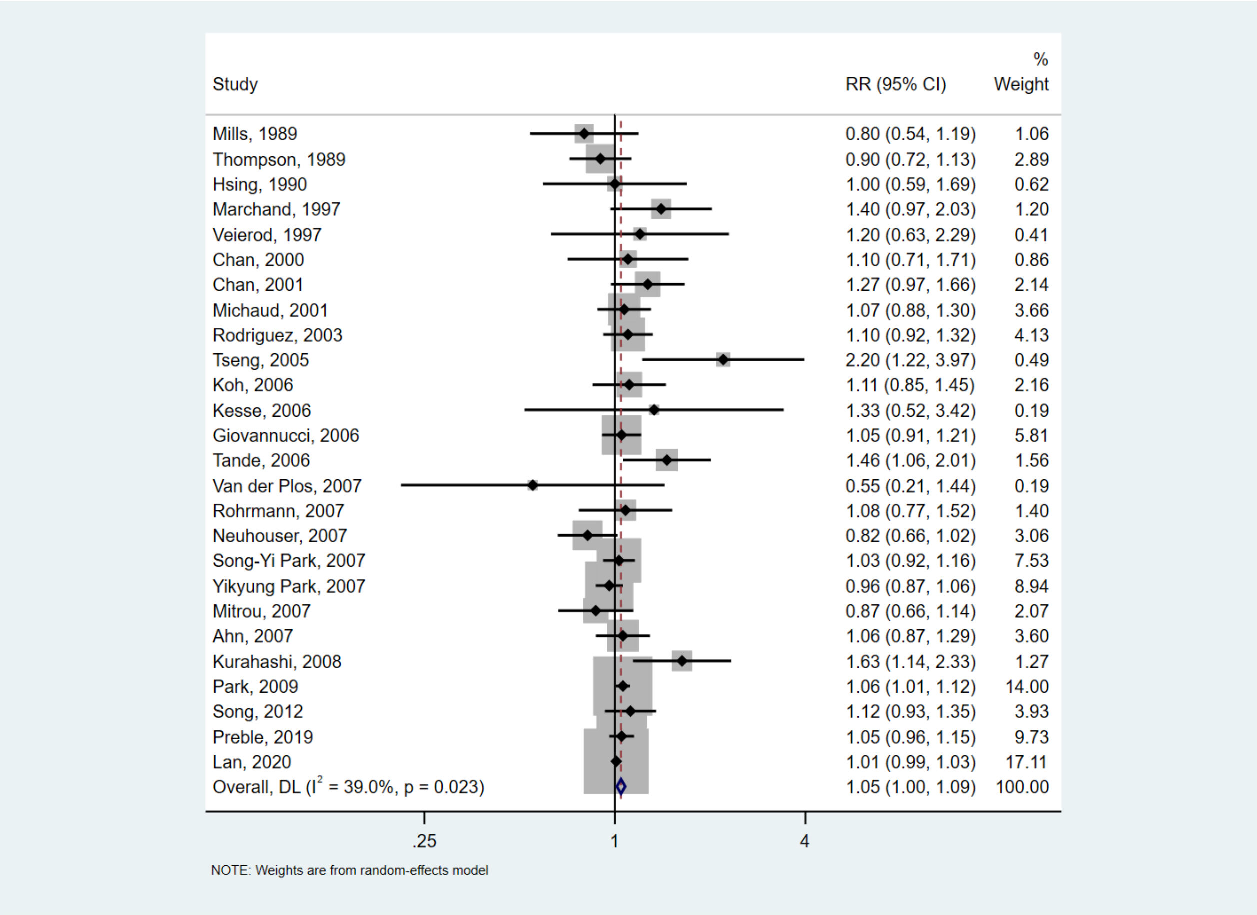

Category: Diet and nutrition
Factor: Egg consumption
Comparison: increase of 5 eggs

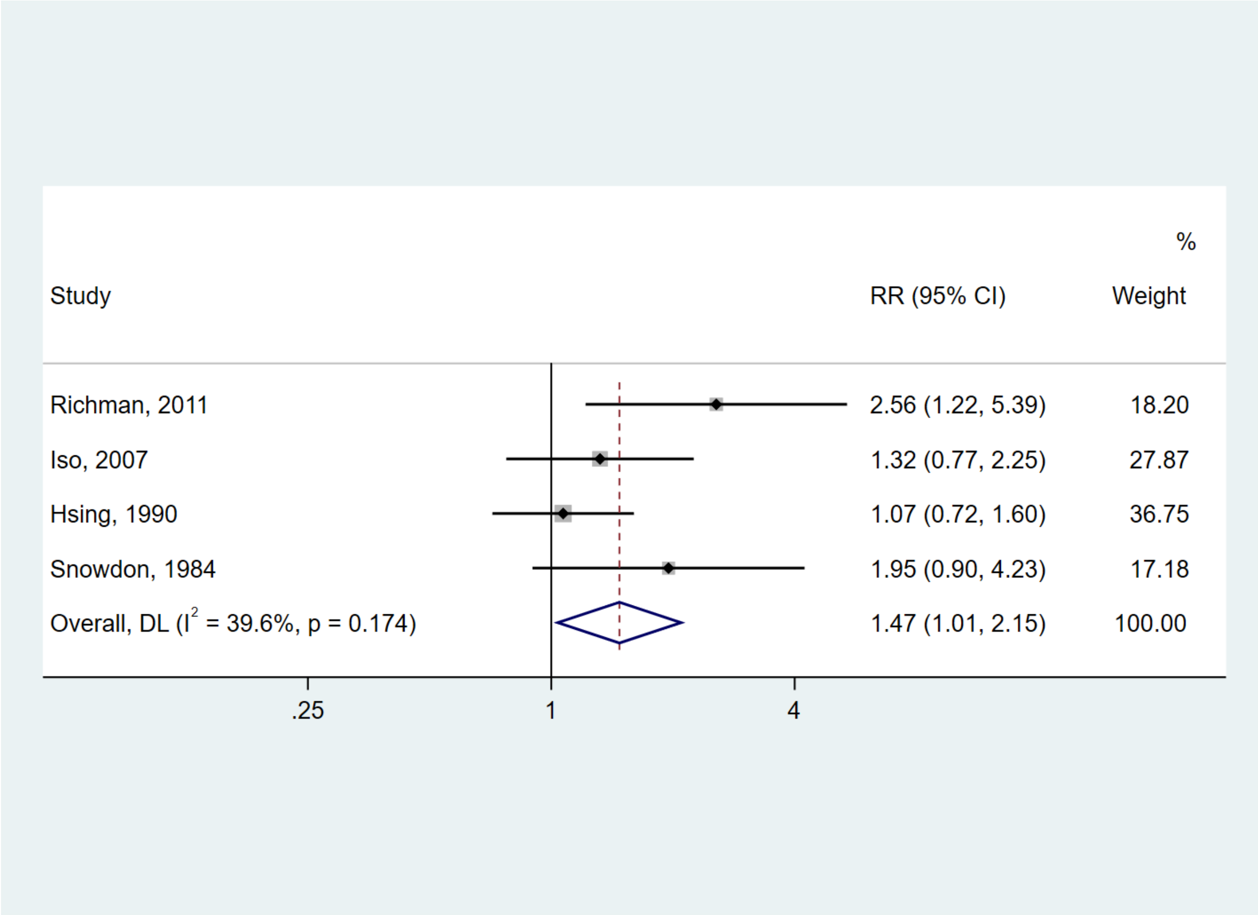

Category: Diet and nutrition
Factor: Processed meat
Comparison: highest vs lowest

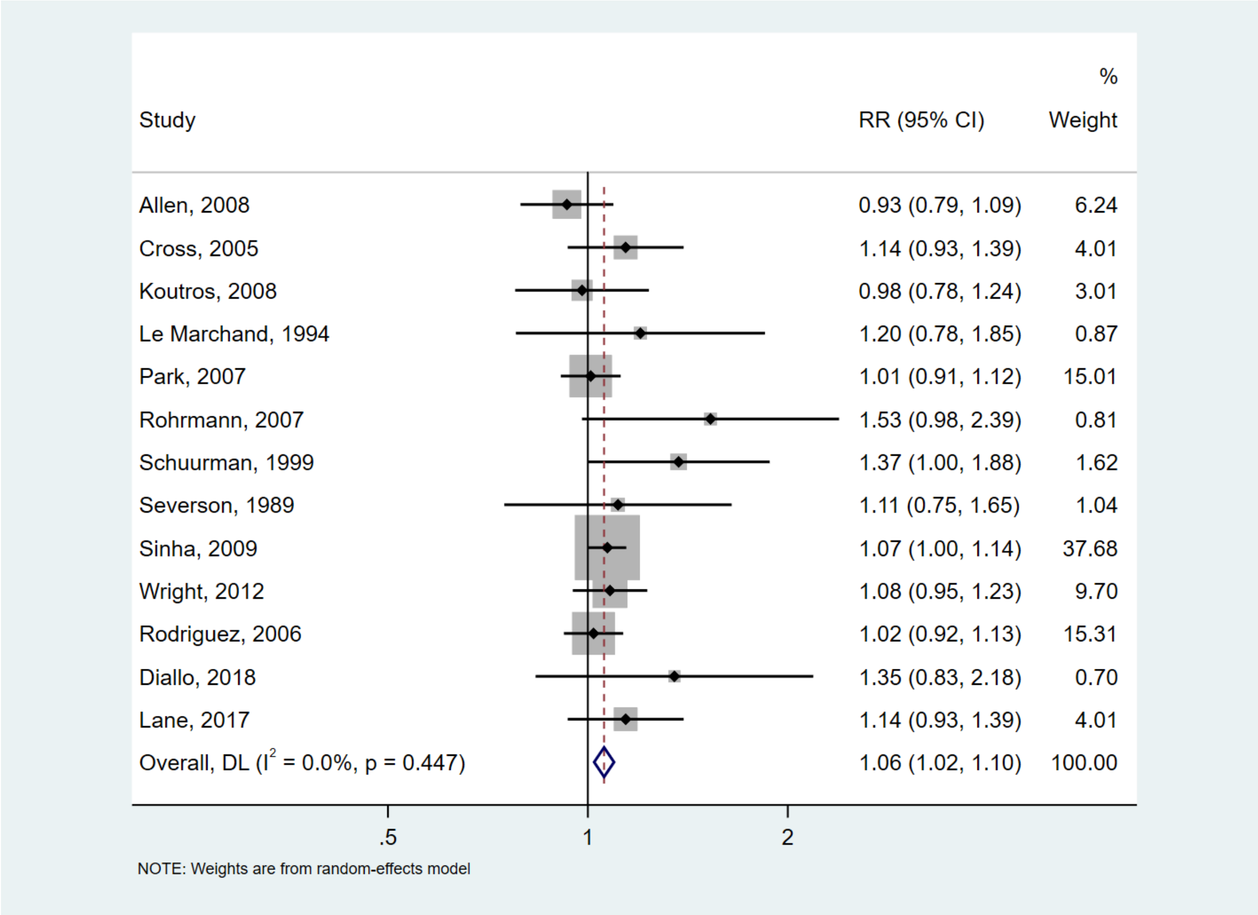

Category: Diet and nutrition
Factor: Soy consumption
Comparison: highest vs lowest

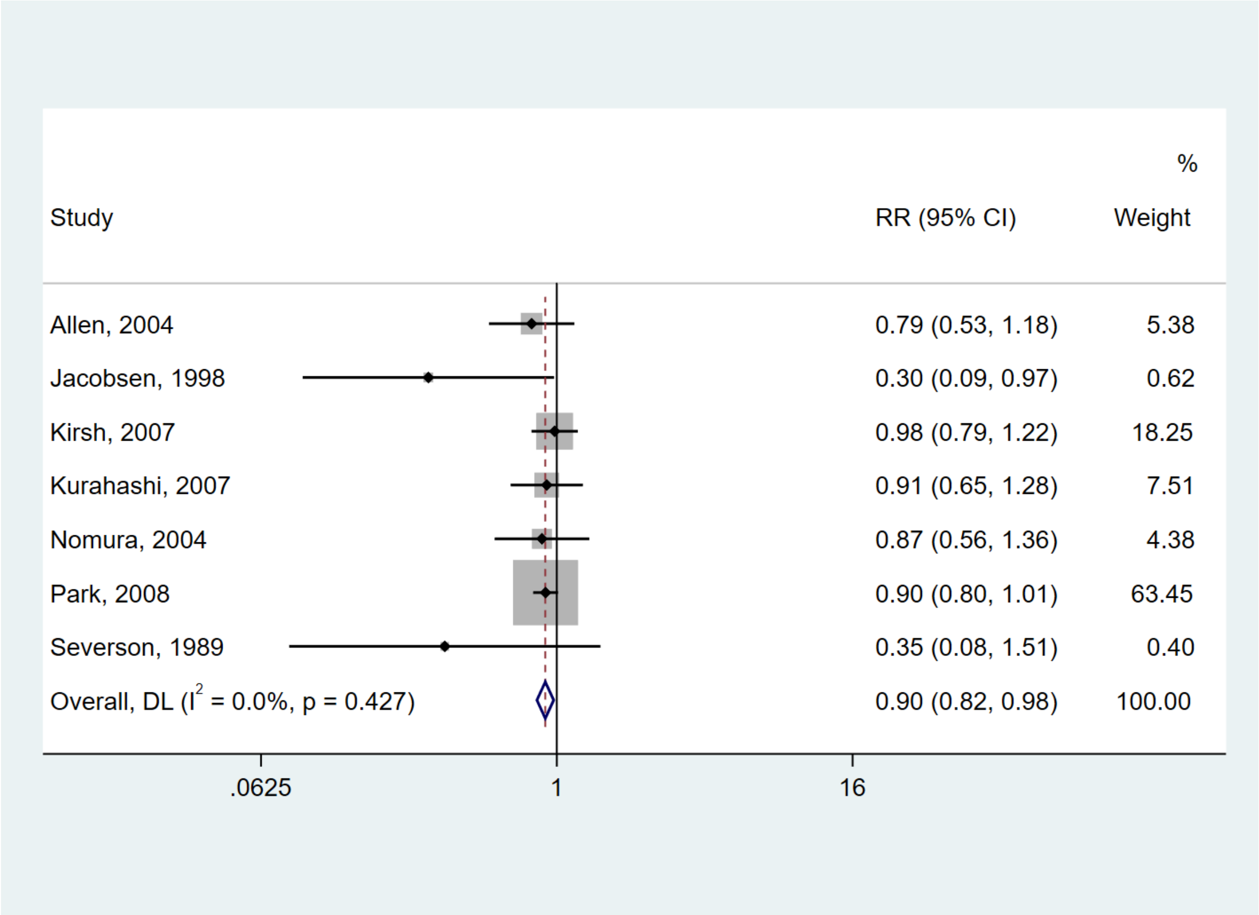

Category: Diet and nutrition
Factor: Sweetened beverage
Comparison: highest vs lowest

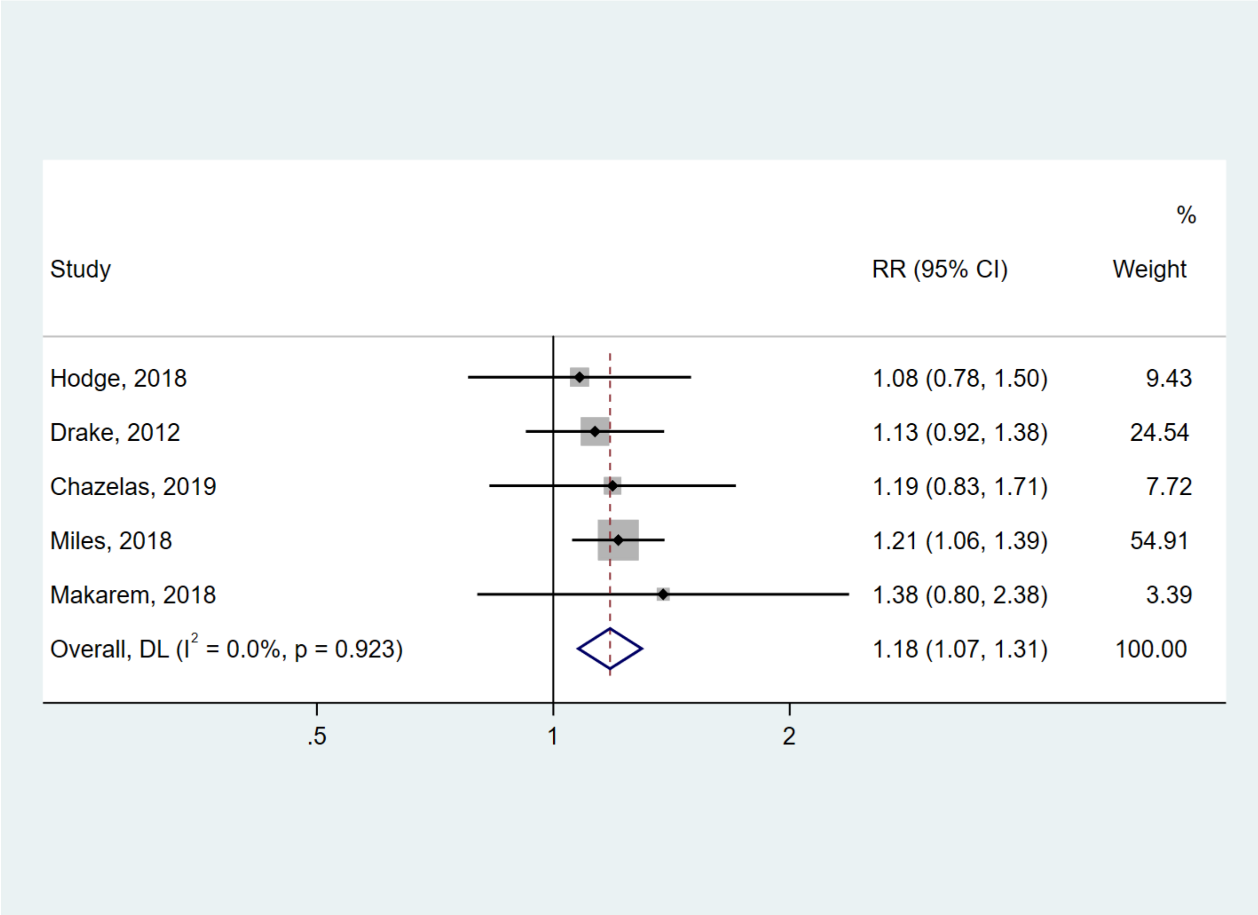

Category: Environmental factors
Factor: Asbestos
Comparison: exposed vs unexposed

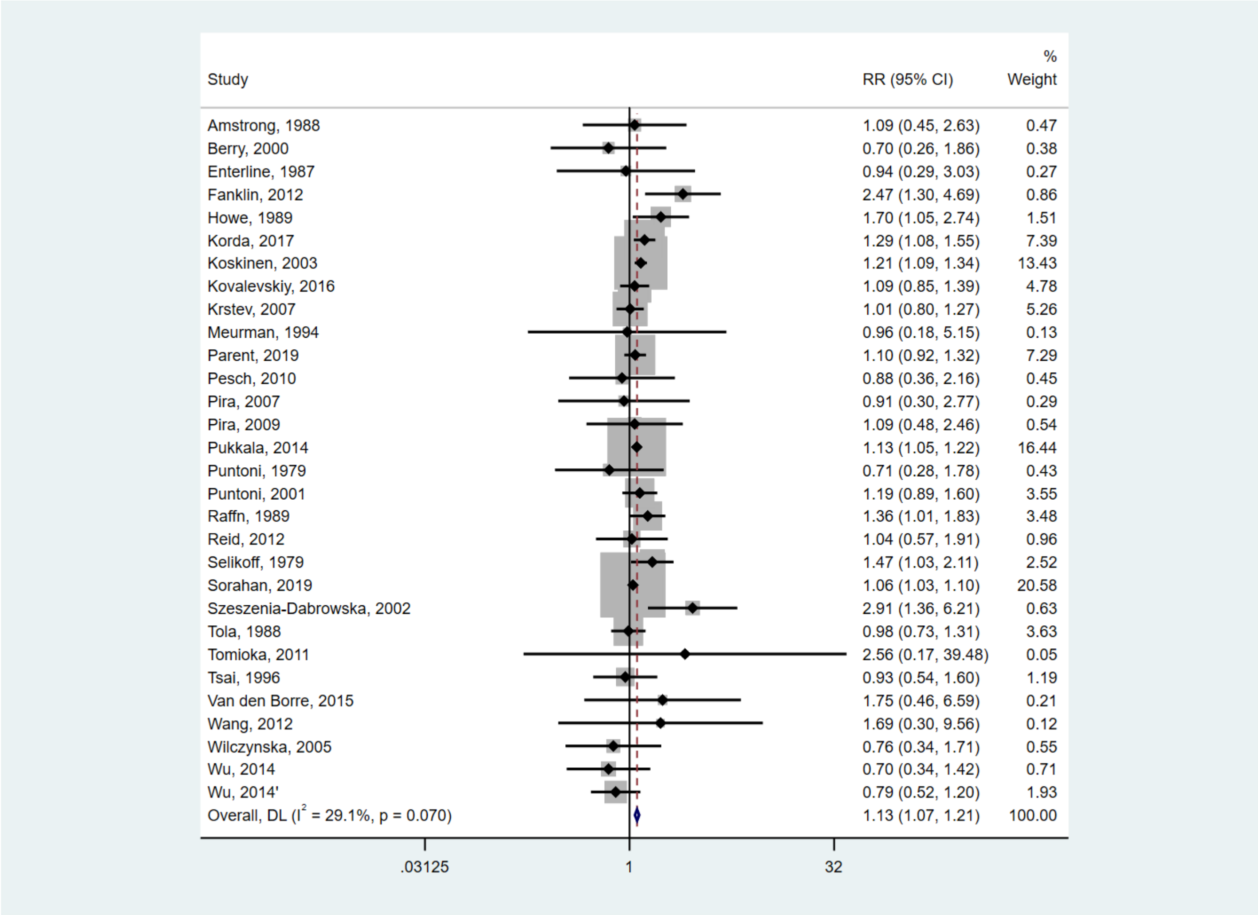

Category: Environmental factors
Factor: Cobalt
Comparison: exposed vs unexposed

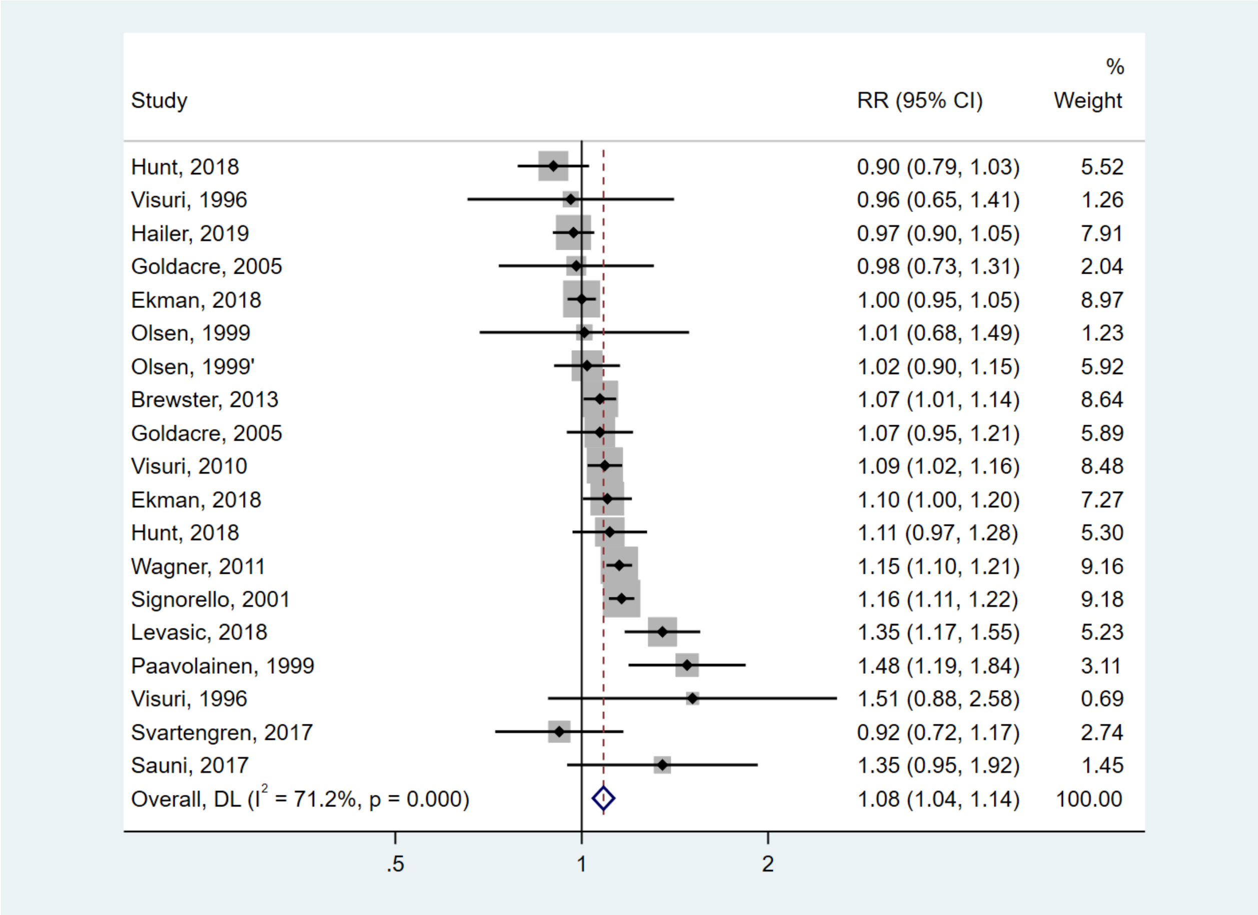

Supplement: S1 Fig — The effect estimates are presented as risk ratios (RR) with 95% confidence intervals (95% CI). (PDF) [file pmed.1004362.s004.pdf]
