# Supplementary material for: Risk factors for prostate cancer: An umbrella review of prospective observational studies and mendelian randomization analyses
Source: PLoS Med. 2024 Mar 15;21(3):e1004362. doi: 10.1371/journal.pmed.1004362 (PMC10980219; doi:10.1371/journal.pmed.1004362)
Supplement: S2 Fig — The 2 dashed line indicated the odds ratios derived from the common effect model (the loosely dashed line) and from random-effects model (the densely dashed line), respectively. W, white population; non-W, non-white population; CI, confidence interval. (PDF) [file pmed.1004362.s005.pdf]

Category: Clinical variables, diseases, and treatments
Factor: Regular use of aspirin
Comparison: users vs non-users

Study	logOR	SE(logOR)	OR	95%-CI	Weight (common)	Weight (random)
ethnicity = W						
Paganini-Hill, 1989	-0.0513	0.1844	0.95	[0.66; 1.36]	0.1%	1.9%
Habel, 2002	-0.2744	0.1268	0.76	[0.59; 0.97]	0.3%	3.3%
Ratnasinghe, 2004	0.1044	0.3134	1.11	[0.60; 2.05]	0.0%	0.8%
Platz, 2005	-0.2744	0.1745	0.76	[0.54; 1.07]	0.1%	2.1%
Jacobs, 2007	0.0198	0.0474	1.02	[0.93; 1.12]	1.9%	6.9%
Siemes, 2008	0.0392	0.1803	1.04	[0.73; 1.48]	0.1%	2.0%
Brasky, 2010	-0.0202	0.0569	0.98	[0.88; 1.10]	1.4%	6.4%
Shebl, 2012	-0.0834	0.0389	0.92	[0.85; 0.99]	2.9%	7.4%
Hollestein, 2014	0.0198	0.0200	1.02	[0.98; 1.06]	10.9%	8.2%
Nordstrom, 2015	0.1044	0.0595	1.11	[0.99; 1.25]	1.2%	6.3%
Lapi, 2016	-0.4463	0.1488	0.64	[0.48; 0.86]	0.2%	2.7%
Skriver, 2016	-0.0619	0.0163	0.94	[0.91; 0.97]	16.5%	8.3%
Downer, 2017	-0.4155	0.1333	0.66	[0.51; 0.86]	0.2%	3.1%
Ma, 2018	-0.1393	0.0087	0.87	[0.86; 0.89]	57.2%	8.4%
Hurtwitz, 2019	0.0488	0.0748	1.05	[0.91; 1.22]	0.8%	5.5%
Downer, 2019	-0.2231	0.0956	0.80	[0.66; 0.96]	0.5%	4.5%
Ajrouche, 2019	-0.2357	0.0548	0.79	[0.71; 0.88]	1.5%	6.5%
Prause, 2020	-0.1744	0.1617	0.84	[0.61; 1.15]	0.2%	2.4%
Skriver, 2020	0.0198	0.0625	1.02	[0.90; 1.15]	1.1%	6.1%
**Common effect model**			**0.91**	**[0.89; 0.92]**	**97.3%**	**--**
**Random effects model**			**0.92**	**[0.86; 0.98]**	**--**	**92.7%**
Heterogeneity: $I^2 = 84\%$, $\tau^2 = 0.0118$, $p < 0.01$						
ethnicity = non-W						
Tsoi, 2019	-0.0513	0.0402	0.95	[0.88; 1.03]	2.7%	7.3%
**Common effect model**			**0.91**	**[0.90; 0.92]**	**100.0%**	**--**
**Random effects model**			**0.92**	**[0.87; 0.97]**	**--**	**100.0%**

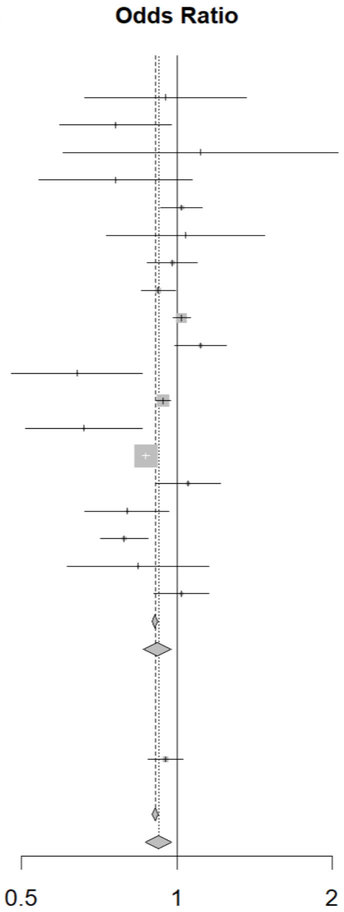

Heterogeneity: $I^2 = 83\%$, $\tau^2 = 0.0101$, $p < 0.01$
Test for subgroup differences (common effect): $\chi^2_1 = 1.35$, df = 1 ($p = 0.24$)
Test for subgroup differences (random effects): $\chi^2_1 = 0.50$, df = 1 ($p = 0.48$)

Category: Diet and nutrition
Factor: Total calcium intake
Comparison: per 400mg/d

Study	logOR	SE(logOR)	OR	95%-CI	Weight (common)	Weight (random)
**ethnicity = non-W**						
Butiler, 2010	0.1484	0.1428	1.16	[0.88; 1.53]	0.1%	0.3%
**ethnicity = W**						
Kristal, 2010	0.0100	0.0178	1.01	[0.98; 1.05]	8.5%	13.5%
Park, 2009	0.0100	0.0075	1.01	[1.00; 1.03]	47.3%	31.3%
Ahn, 2007	0.0198	0.0274	1.02	[0.97; 1.08]	3.6%	6.9%
Park, 2007	0.0100	0.0226	1.01	[0.97; 1.06]	5.2%	9.5%
Rohrmann, 2007	-0.0101	0.1296	0.99	[0.77; 1.28]	0.2%	0.4%
Giovannucci, 2006	0.0392	0.0098	1.04	[1.02; 1.06]	27.9%	26.1%
Kesse, 2006	0.4447	0.2755	1.56	[0.91; 2.68]	0.0%	0.1%
Rodriguez, 2003	0.0488	0.0194	1.05	[1.01; 1.09]	7.1%	11.9%
**Common effect model**			**1.02**	**[1.01; 1.03]**	**99.9%**	**--**
**Random effects model**			**1.02**	**[1.01; 1.04]**	**--**	**99.7%**
Heterogeneity: $I^2 = 34\%$, $\tau^2 = 0.0001$, $p = 0.15$						
**Common effect model**			**1.02**	**[1.01; 1.03]**	**100.0%**	**--**
**Random effects model**			**1.02**	**[1.01; 1.04]**	**--**	**100.0%**

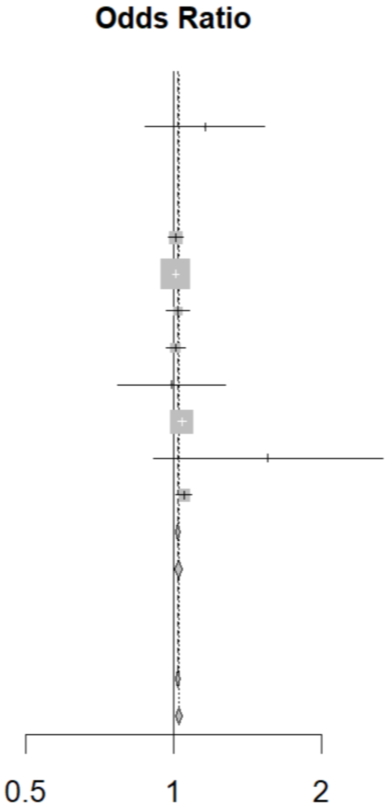

Heterogeneity: $I^2 = 30\%$, $\tau^2 = 0.0001$, $p = 0.18$
Test for subgroup differences (common effect): $\chi^2_1 = 0.79$, df = 1 ($p = 0.37$)
Test for subgroup differences (random effects): $\chi^2_1 = 0.77$, df = 1 ($p = 0.38$)

Category: Lifestyle
Factor: Coffee
Comparison: highest vs lowest

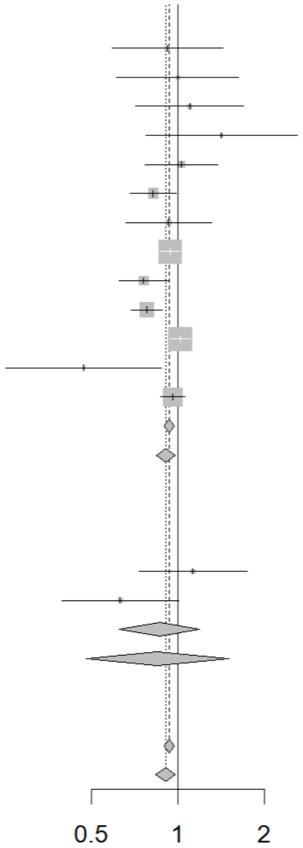

Study	logOR	SE(logOR)	OR	95%-CI	Weight (common)	Weight (random)
ethnicity = W						
Severson, 1989	-0.0834	0.2276	0.92	[0.59; 1.44]	0.9%	2.6%
Hsing, 1990	0.0000	0.2502	1.00	[0.61; 1.63]	0.7%	2.2%
Le Marchard, 1994	0.0953	0.2227	1.10	[0.71; 1.70]	0.9%	2.7%
Ellison, 2000	0.3507	0.3114	1.42	[0.77; 2.61]	0.5%	1.5%
Nilsson, 2010	0.0296	0.1488	1.03	[0.77; 1.38]	2.0%	5.1%
Wilson, 2011	-0.1985	0.0932	0.82	[0.68; 0.98]	5.1%	9.2%
Shafique, 2012	-0.0726	0.1749	0.93	[0.66; 1.31]	1.4%	4.0%
Bosire, 2013	-0.0619	0.0406	0.94	[0.87; 1.02]	26.8%	15.5%
Discacciati, 2013	-0.2744	0.1007	0.76	[0.62; 0.93]	4.4%	8.4%
Terdal, 2015	-0.2485	0.0649	0.78	[0.69; 0.89]	10.5%	12.4%
Hashibe, 2015	0.0198	0.0401	1.02	[0.94; 1.10]	27.5%	15.5%
Pounis, 2017	-0.7550	0.3181	0.47	[0.25; 0.88]	0.4%	1.4%
Sen, 2019	-0.0408	0.0504	0.96	[0.87; 1.06]	17.4%	14.2%
Common effect model			**0.93**	**[0.89; 0.97]**	**98.3%**	**--**
Random effects model			**0.91**	**[0.84; 0.98]**	**--**	**94.9%**
Heterogeneity: $I^2 = 55\%$, $\tau^2 = 0.0082$, $p < 0.01$						
ethnicity = non-W						
Iso, 2007	0.1222	0.2230	1.13	[0.73; 1.75]	0.9%	2.7%
Li, 2013	-0.4620	0.2402	0.63	[0.39; 1.01]	0.8%	2.4%
Common effect model			**0.86**	**[0.63; 1.19]**	**1.7%**	**--**
Random effects model			**0.85**	**[0.48; 1.51]**	**--**	**5.1%**
Heterogeneity: $I^2 = 69\%$, $\tau^2 = 0.1170$, $p = 0.07$						
**Common effect model**			**0.93**	**[0.89; 0.97]**	**100.0%**	**--**
**Random effects model**			**0.91**	**[0.84; 0.98]**	**--**	**100.0%**

Heterogeneity: $I^2 = 53\%$, $\tau^2 = 0.0085$, $p < 0.01$
Test for subgroup differences (common effect): $\chi^2_1 = 0.23$, df = 1 ($p = 0.63$)
Test for subgroup differences (random effects): $\chi^2_1 = 0.05$, df = 1 ($p = 0.82$)

Category: Lifestyle
Factor: Current smoking
Comparison: current smoking vs non-smoker

Study	logOR	SE(logOR)	OR	95%−CI	Weight (common)	Weight (random)
ethnicity = W						
Thompson, 1989	0.2624	0.2678	1.30	[0.77; 2.20]	0.1%	1.9%
Severson, 1989	−0.2614	0.1534	0.77	[0.57; 1.04]	0.3%	3.1%
Le Marchand, 1994	−0.0408	0.1283	0.96	[0.75; 1.23]	0.4%	3.5%
Adami, 1996	−0.1744	0.0426	0.84	[0.77; 0.91]	3.9%	4.5%
Cerhan, 1997	0.4574	0.2765	1.58	[0.92; 2.72]	0.1%	1.8%
Will, 1999	0.0000	0.0409	1.00	[0.92; 1.08]	4.3%	4.5%
Lund Nilsen, 2000	−0.3425	0.0931	0.71	[0.59; 0.85]	0.8%	3.9%
Lotufo, 2000	−0.1393	0.1046	0.87	[0.71; 1.07]	0.7%	3.8%
Baglietto, 2006	−0.3711	0.1170	0.69	[0.55; 0.87]	0.5%	3.6%
Gonzalez, 2007	−0.1985	0.1303	0.82	[0.64; 1.06]	0.4%	3.5%
Park, 2007	−0.1985	0.0403	0.82	[0.76; 0.89]	4.4%	4.5%
Rohrmann, 2007 (1963)	−1.0217	0.1868	0.36	[0.25; 0.52]	0.2%	2.7%
Rohrmann, 2007 (1975)	−0.7985	0.1289	0.45	[0.35; 0.58]	0.4%	3.5%
Watters, 2009	−0.2485	0.0262	0.78	[0.74; 0.82]	10.4%	4.6%
Grundmark, 2011	−0.4005	0.1313	0.67	[0.52; 0.87]	0.4%	3.4%
Karppi, 2012	−0.7133	0.3251	0.49	[0.26; 0.93]	0.1%	1.5%
Shafique, 2012	−0.6733	0.1096	0.51	[0.41; 0.63]	0.6%	3.7%
Everatt, 2014	−0.6162	0.1169	0.54	[0.43; 0.68]	0.5%	3.6%
Ho, 2014	0.0488	0.0846	1.05	[0.89; 1.24]	1.0%	4.1%
Perez−Cornago, 2017	−0.3567	0.0545	0.70	[0.63; 0.78]	2.4%	4.4%
Jacob, 2018	−0.3425	0.0399	0.71	[0.66; 0.77]	4.5%	4.5%
Viner, 2019	−0.4308	0.1517	0.65	[0.48; 0.88]	0.3%	3.2%
Weber, 2021	−0.4943	0.0662	0.61	[0.54; 0.69]	1.6%	4.3%
Jochems, 2022	−0.0726	0.0110	0.93	[0.91; 0.95]	59.2%	4.6%
**Common effect model**			**0.87**	**[0.85; 0.88]**	**97.6%**	**--**
**Random effects model**			**0.74**	**[0.67; 0.83]**	**--**	**86.7%**
Heterogeneity: $I^2 = 91\%$, $\tau^2 = 0.0600$, $p < 0.01$						
ethnicity = non−W						
Butler, 2009	−0.3285	0.1394	0.72	[0.55; 0.95]	0.4%	3.3%
Li, 2011	−0.4463	0.1344	0.64	[0.49; 0.83]	0.4%	3.4%
Bae, 2013	−0.2485	0.2161	0.78	[0.51; 1.19]	0.2%	2.4%
Sawada, 2014	−0.4155	0.0700	0.66	[0.58; 0.76]	1.5%	4.2%
**Common effect model**			**0.67**	**[0.60; 0.75]**	**2.4%**	**--**
**Random effects model**			**0.67**	**[0.60; 0.75]**	**--**	**13.3%**
Heterogeneity: $I^2 = 0\%$, $\tau^2 = 0$, $p = 0.82$						
**Common effect model**			**0.86**	**[0.85; 0.88]**	**100.0%**	**--**
**Random effects model**			**0.74**	**[0.67; 0.81]**	**--**	**100.0%**

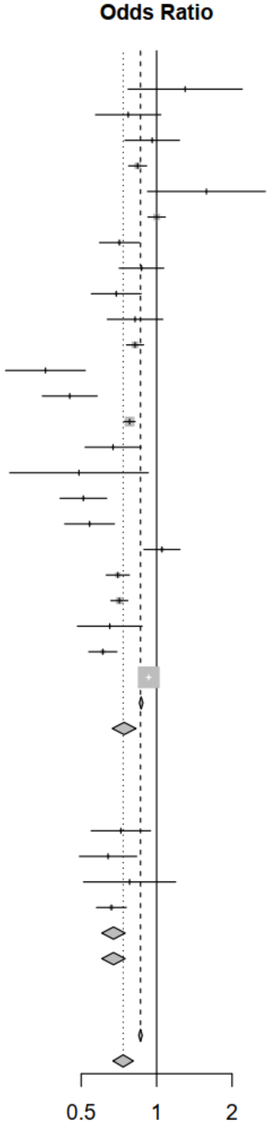

Heterogeneity: $I^2 = 90\%$, $\tau^2 = 0.0493$, $p < 0.01$
Test for subgroup differences (common effect): $\chi^2_1 = 20.95$, df = 1 ($p < 0.01$)
Test for subgroup differences (random effects): $\chi^2_1 = 1.57$, df = 1 ($p = 0.21$)

Category: Diet and nutrition
Factor: Daidzein
Comparison: highest vs lowest

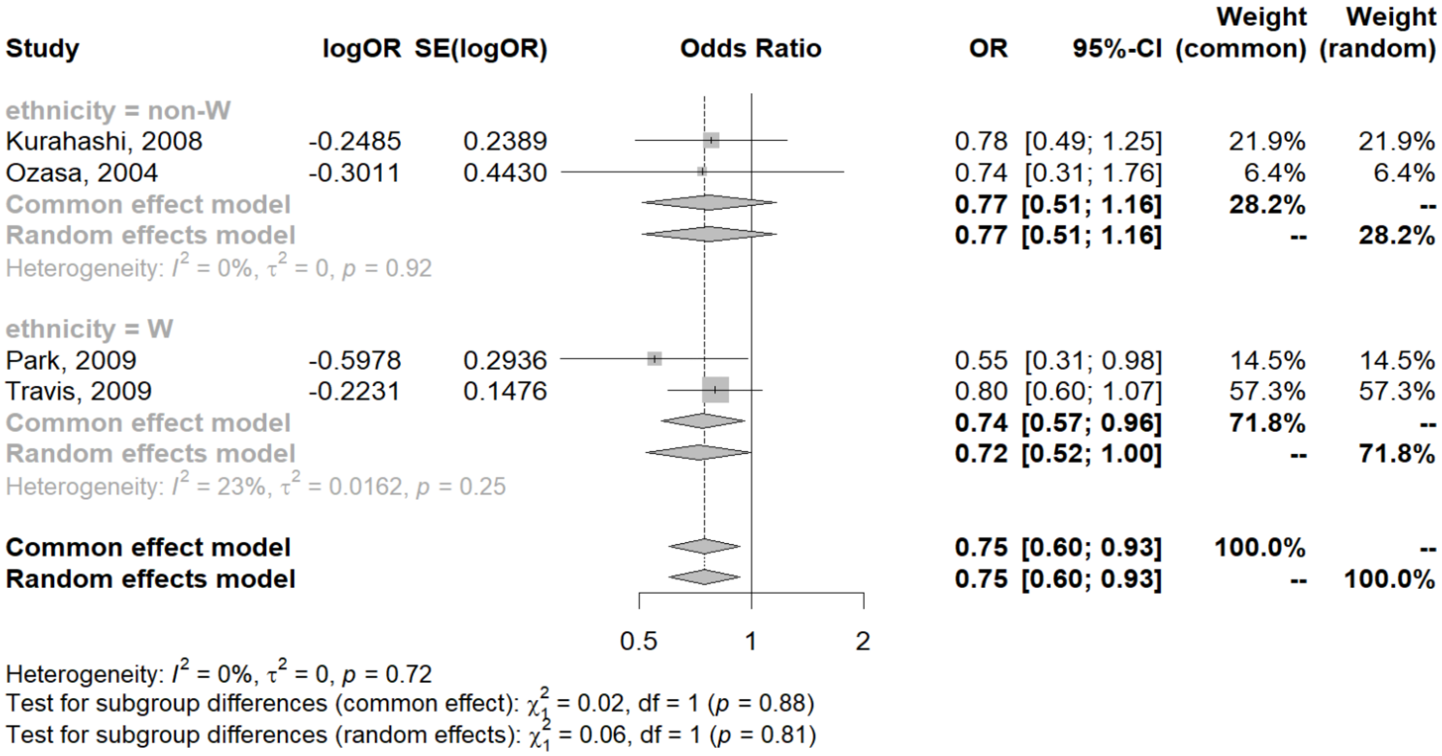

Category: Clinical variables, diseases, and treatments
Factor: Finasteride
Comparison: users vs non-users

Study	logOR	SE(logOR)	OR	95%-CI	Weight (common)	Weight (random)
ethnicity = W						
Ian, 2003	-0.3567	0.0512	0.70	[0.63; 0.77]	11.2%	13.0%
Lucia, 2007	-0.5276	0.0521	0.59	[0.53; 0.65]	10.8%	13.0%
Marry, 2008	-0.2877	0.0471	0.75	[0.68; 0.82]	13.2%	13.1%
Steven, 2009	0.5596	0.0553	1.75	[1.57; 1.95]	9.6%	13.0%
Thompson, 2006	-0.4463	0.0552	0.64	[0.57; 0.71]	9.7%	13.0%
Murtola, 2009	-0.8916	0.0308	0.41	[0.39; 0.44]	31.1%	13.1%
**Common effect model**			**0.63**	**[0.60; 0.65]**	**85.7%**	**--**
**Random effects model**			**0.72**	**[0.49; 1.06]**	**--**	**78.2%**
Heterogeneity: $I^2 = 99\%$, $\tau^2 = 0.2299$, $p < 0.01$						
ethnicity = non-W						
Niu, 2009	-0.5276	0.3001	0.59	[0.33; 1.06]	0.3%	8.7%
Zhu, 2009	-0.4005	0.0458	0.67	[0.61; 0.73]	14.0%	13.1%
**Common effect model**			**0.67**	**[0.61; 0.73]**	**14.3%**	**--**
**Random effects model**			**0.67**	**[0.61; 0.73]**	**--**	**21.8%**
Heterogeneity: $I^2 = 0\%$, $\tau^2 = 0$, $p = 0.68$						
**Common effect model**			**0.63**	**[0.61; 0.65]**	**100.0%**	**--**
**Random effects model**			**0.70**	**[0.52; 0.95]**	**--**	**100.0%**

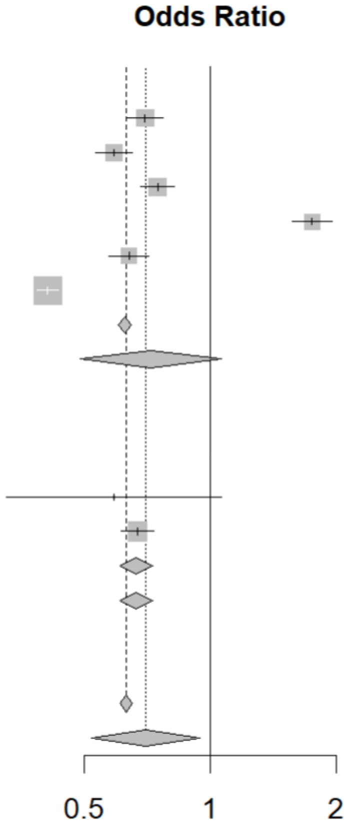

Heterogeneity: $I^2 = 99\%$, $\tau^2 = 0.1739$, $p < 0.01$
Test for subgroup differences (common effect): $\chi^2_1 = 1.79$, df = 1 ($p = 0.18$)
Test for subgroup differences (random effects): $\chi^2_1 = 0.15$, df = 1 ($p = 0.70$)

Category: Lifestyle
Factor: Firefighter
Comparison: ever-employment as a career firefighter vs general population

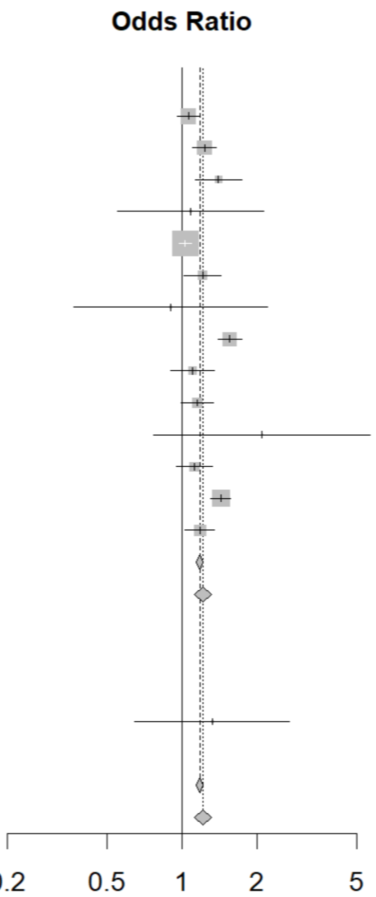

Study	logOR	SE(logOR)	OR	95%-CI	Weight (common)	Weight (random)
ethnicity = W						
Bigert, 2020	0.0583	0.0505	1.06	[0.96; 1.17]	11.3%	9.9%
Glass, 2016	0.2070	0.0560	1.23	[1.10; 1.37]	9.2%	9.6%
Demers, 1994	0.3365	0.1101	1.40	[1.13; 1.74]	2.4%	6.4%
Bates, 2001	0.0770	0.3430	1.08	[0.55; 2.12]	0.2%	1.3%
Daniels, 2014	0.0296	0.0298	1.03	[0.97; 1.09]	32.4%	11.0%
Pukkala, 2014 (Finland)	0.1906	0.0862	1.21	[1.02; 1.43]	3.9%	7.7%
Pukkala, 2014 (Iceland)	-0.1054	0.4536	0.90	[0.37; 2.19]	0.1%	0.8%
Webber, 2021	0.4383	0.0558	1.55	[1.39; 1.73]	9.2%	9.6%
Ma, 2006	0.0953	0.1015	1.10	[0.90; 1.34]	2.8%	6.8%
Harris, 2018	0.1398	0.0772	1.15	[0.99; 1.34]	4.8%	8.3%
Giles, 1993	0.7372	0.5080	2.09	[0.77; 5.66]	0.1%	0.6%
Peterson, 2018	0.1133	0.0858	1.12	[0.95; 1.33]	3.9%	7.8%
Sritharan, 2022	0.3577	0.0462	1.43	[1.31; 1.57]	13.5%	10.2%
Marjerrison, 2022	0.1655	0.0690	1.18	[1.03; 1.35]	6.0%	8.8%
Common effect model			**1.18**	**[1.14; 1.22]**	**99.8%**	**--**
Random effects model			**1.21**	**[1.12; 1.32]**	**--**	**98.8%**
Heterogeneity: $I^2 = 82\%$, $\tau^2 = 0.0148$, $p < 0.01$						
ethnicity = non-W						
Ahn, 2012	0.2776	0.3633	1.32	[0.65; 2.69]	0.2%	1.2%
**Common effect model**			**1.18**	**[1.14; 1.22]**	**100.0%**	**--**
**Random effects model**			**1.21**	**[1.12; 1.32]**	**--**	**100.0%**

Heterogeneity: $I^2 = 81\%$, $\tau^2 = 0.0146$, $p < 0.01$
Test for subgroup differences (common effect): $\chi^2_1 = 0.10$, df = 1 ($p = 0.75$)
Test for subgroup differences (random effects): $\chi^2_1 = 0.05$, df = 1 ($p = 0.82$)

Category: Anthropometric indices
Factor: Height
Comparison: per 10 cm increase

Study	logOR	SE(logOR)	OR	95%−CI	Weight (common)	Weight (random)
ethnicity = W						
Zuccolo, 2008	0.0583	0.0456	1.06	[0.97; 1.16]	2.5%	5.5%
Littman, 2007	0.1398	0.0510	1.15	[1.04; 1.27]	2.0%	4.7%
Sequoia, 2006	0.1133	0.0453	1.12	[1.02; 1.22]	2.6%	5.6%
Batty, 2006	0.1310	0.0753	1.14	[0.98; 1.32]	0.9%	2.5%
Lund Haheim, 2006	0.0000	0.0763	1.00	[0.86; 1.16]	0.9%	2.4%
Piatz, 2004	0.0000	0.0962	1.00	[0.83; 1.21]	0.6%	1.6%
Piatz, 2004'	−0.1278	0.1378	0.88	[0.67; 1.15]	0.3%	0.8%
Stattin, 2004	0.1823	0.1259	1.20	[0.94; 1.54]	0.3%	1.0%
Engeland, 2003	0.1133	0.0091	1.12	[1.10; 1.14]	63.5%	18.2%
Gunnell, 2003	−0.2107	0.2875	0.81	[0.46; 1.42]	0.1%	0.2%
Jonsson, 2003	0.0000	0.0889	1.00	[0.84; 1.19]	0.7%	1.8%
Lamharzi, 2003	−0.0202	0.1109	0.98	[0.79; 1.22]	0.4%	1.2%
MacInnis, 2003	−0.0619	0.0757	0.94	[0.81; 1.09]	0.9%	2.4%
Rodriguez, 2001	0.1222	0.0407	1.13	[1.04; 1.22]	3.2%	6.5%
Rodriguez, 2001'	0.0392	0.0367	1.04	[0.97; 1.12]	3.9%	7.4%
Freeman, 2001	0.0862	0.0606	1.09	[0.97; 1.23]	1.4%	3.6%
Davey Smith, 2000	−0.2614	0.1931	0.77	[0.53; 1.12]	0.1%	0.4%
Habel, 2000	0.0770	0.0331	1.08	[1.01; 1.15]	4.8%	8.4%
Putnam, 2000	0.0677	0.1819	1.07	[0.75; 1.53]	0.2%	0.5%
Schuurman, 2000	−0.0202	0.0726	0.98	[0.85; 1.13]	1.0%	2.6%
Lund Nilsen, 1999	0.1740	0.0920	1.19	[0.99; 1.43]	0.6%	1.7%
Andersson, 1997	0.0862	0.0397	1.09	[1.01; 1.18]	3.3%	6.7%
Cerhan, 1997	−0.0619	0.2380	0.94	[0.59; 1.50]	0.1%	0.3%
Herbet, 1997	0.1989	0.0438	1.22	[1.12; 1.33]	2.7%	5.9%
Tulinius, 1997	0.0953	0.0761	1.10	[0.95; 1.28]	0.9%	2.4%
Veierod, 1997	−0.0101	0.1695	0.99	[0.71; 1.38]	0.2%	0.5%
Le Marchand, 1994	0.3784	0.1497	1.46	[1.09; 1.96]	0.2%	0.7%
Thune, 1994	−0.0101	0.0950	0.99	[0.82; 1.19]	0.6%	1.6%
Albanes, 1988	0.0677	0.1634	1.07	[0.78; 1.47]	0.2%	0.6%
Greenwald, 1974	0.0677	0.1131	1.07	[0.86; 1.34]	0.4%	1.2%
**Common effect model**			**1.11**	**[1.09; 1.12]**	**99.6%**	**--**
**Random effects model**			**1.09**	**[1.06; 1.12]**	**--**	**98.9%**
Heterogeneity: $I^2 = 26\%$, $\tau^2 = 0.0008$, $p = 0.09$						
ethnicity = non−W						
Kurahashi, 2006	0.1133	0.1192	1.12	[0.89; 1.41]	0.4%	1.1%
**Common effect model**			**1.11**	**[1.09; 1.12]**	**100.0%**	**--**
**Random effects model**			**1.09**	**[1.06; 1.12]**	**--**	**100.0%**

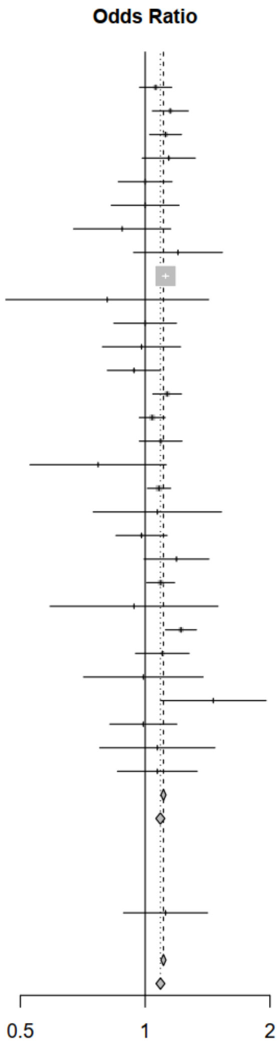

Heterogeneity: $I^2 = 24\%$, $\tau^2 = 0.0008$, $p = 0.12$
Test for subgroup differences (common effect): $\chi^2_1 = 0.01$, df = 1 ($p = 0.92$)
Test for subgroup differences (random effects): $\chi^2_1 = 0.06$, df = 1 ($p = 0.81$)

Category: Diet and nutrition
Factor: Soy consumption
Comparison: highest vs lowest

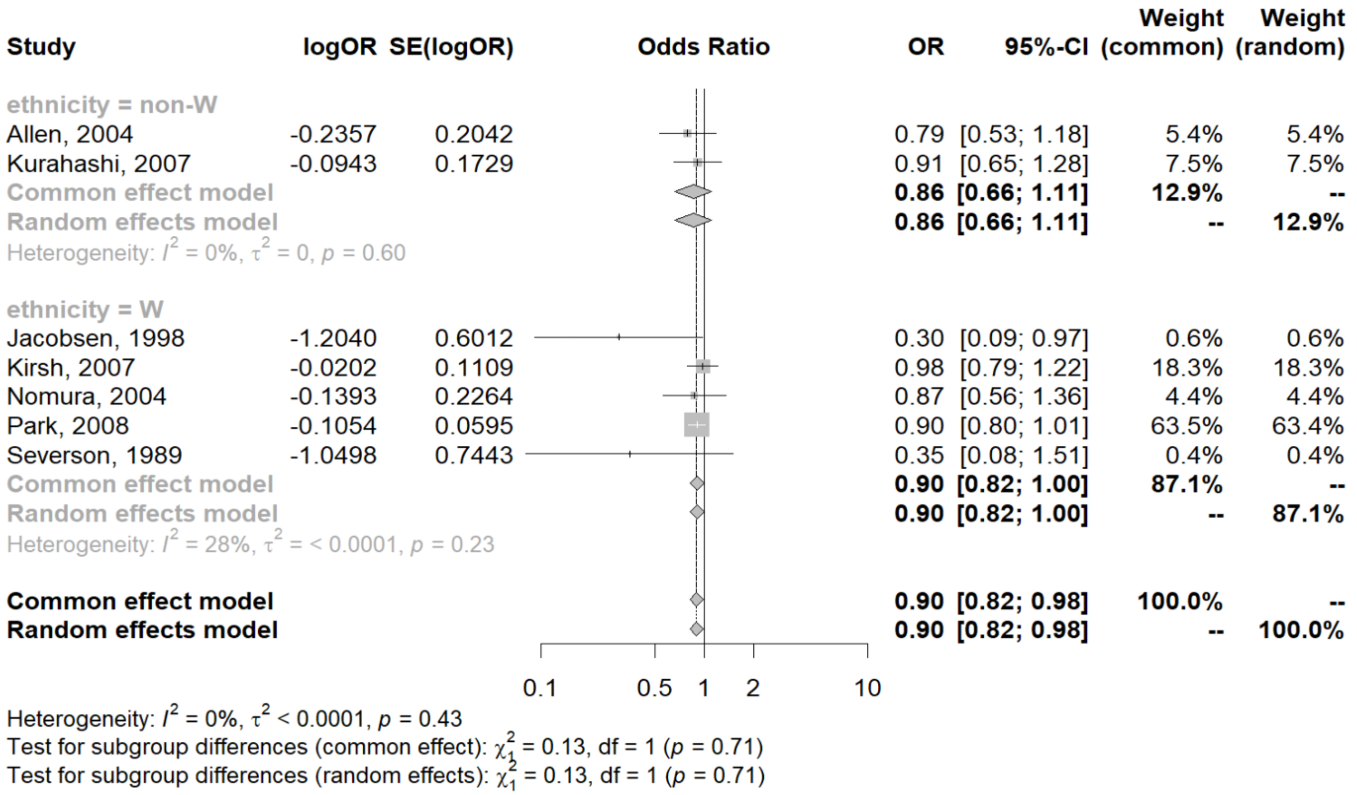

Category: Diet and nutrition
Factor: Total dairy products
Comparison: highest vs lowest

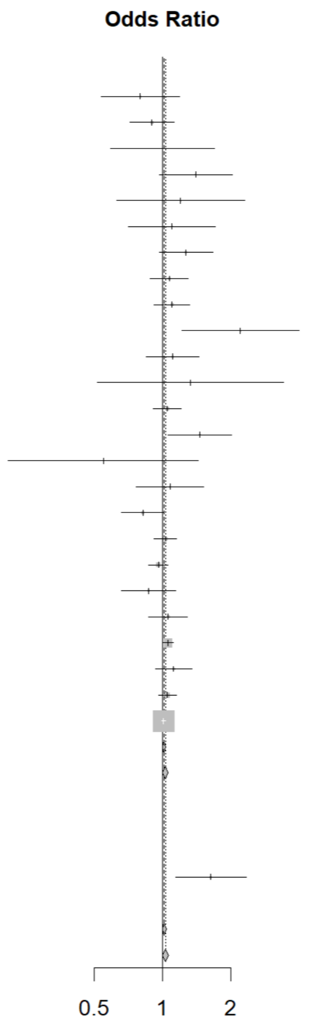

Study	logOR	SE(logOR)	OR	95%-CI	Weight (common)	Weight (random)
ethnicity = W						
Mills, 1989	-0.2231	0.2016	0.80	[0.54; 1.19]	0.2%	0.6%
Thompson, 1989	-0.1054	0.1150	0.90	[0.72; 1.13]	0.6%	1.7%
Hsing, 1990	0.0000	0.2669	1.00	[0.59; 1.69]	0.1%	0.3%
Marchand, 1997	0.3365	0.1884	1.40	[0.97; 2.03]	0.2%	0.7%
Veierod, 1997	0.1823	0.3303	1.20	[0.63; 2.29]	0.1%	0.2%
Chan, 2000	0.0953	0.2242	1.10	[0.71; 1.71]	0.1%	0.5%
Chan, 2001	0.2390	0.1371	1.27	[0.97; 1.66]	0.4%	1.2%
Michaud, 2001	0.0677	0.0995	1.07	[0.88; 1.30]	0.7%	2.2%
Rodriguez, 2003	0.0953	0.0921	1.10	[0.92; 1.32]	0.9%	2.6%
Tseng, 2005	0.7885	0.3010	2.20	[1.22; 3.97]	0.1%	0.3%
Koh, 2006	0.1044	0.1362	1.11	[0.85; 1.45]	0.4%	1.2%
Kesse, 2006	0.2852	0.4812	1.33	[0.52; 3.42]	0.0%	0.1%
Giovannucci, 2006	0.0488	0.0727	1.05	[0.91; 1.21]	1.4%	4.0%
Tande, 2006	0.3784	0.1632	1.46	[1.06; 2.01]	0.3%	0.9%
Van der Plos, 2007	-0.5978	0.4894	0.55	[0.21; 1.44]	0.0%	0.1%
Rohrmann, 2007	0.0770	0.1735	1.08	[0.77; 1.52]	0.2%	0.8%
Neuhouser, 2007	-0.1985	0.1111	0.82	[0.66; 1.02]	0.6%	1.8%
Song-Yi Park, 2007	0.0296	0.0591	1.03	[0.92; 1.16]	2.1%	5.8%
Yikyung Park, 2007	-0.0408	0.0504	0.96	[0.87; 1.06]	2.9%	7.5%
Mitrou, 2007	-0.1393	0.1394	0.87	[0.66; 1.14]	0.4%	1.2%
Ahn, 2007	0.0583	0.1005	1.06	[0.87; 1.29]	0.7%	2.2%
Park, 2009	0.0583	0.0264	1.06	[1.01; 1.12]	10.6%	18.3%
Song, 2012	0.1133	0.0951	1.12	[0.93; 1.35]	0.8%	2.5%
Preble, 2019	0.0488	0.0461	1.05	[0.96; 1.15]	3.5%	8.7%
Lan, 2020	0.0100	0.0101	1.01	[0.99; 1.03]	72.4%	33.9%
Common effect model			**1.02**	**[1.00; 1.04]**	**99.8%**	**--**
Random effects model			**1.03**	**[1.00; 1.06]**	**--**	**99.3%**
Heterogeneity: $I^2 = 30\%$, $\tau^2 = 0.0004$, $p = 0.08$						
ethnicity = non-W						
Kurahashi, 2008	0.4886	0.1824	1.63	[1.14; 2.33]	0.2%	0.7%
**Common effect model**			**1.02**	**[1.00; 1.04]**	**100.0%**	**--**
**Random effects model**			**1.04**	**[1.00; 1.07]**	**--**	**100.0%**

Heterogeneity: $I^2 = 39\%$, $\tau^2 = 0.0006$, $p = 0.02$
Test for subgroup differences (common effect): $\chi^2_1 = 6.61$, df = 1 ($p = 0.01$)
Test for subgroup differences (random effects): $\chi^2_1 = 6.28$, df = 1 ($p = 0.01$)

Category: Clinical variables, diseases, and treatments
Factor: Ulcerative colitis
Comparison: patients vs non-patients

Study	logOR	SE(logOR)	OR	95%-CI	Weight (common)	Weight (random)
ethnicity = W						
Bernstein, 2001	-0.0305	0.2362	0.97	[0.61; 1.54]	1.7%	5.8%
Ekbom, 1991	0.3365	0.2257	1.40	[0.90; 2.18]	1.9%	6.0%
Hemminki, 2008	0.1310	0.2257	1.14	[0.73; 1.77]	1.9%	6.0%
Jess, 2013	0.5988	0.2253	1.82	[1.17; 2.83]	1.9%	6.1%
Jussila, 2013	-0.1625	0.0838	0.85	[0.72; 1.00]	13.4%	9.2%
Kappelman, 2014	0.1823	0.0446	1.20	[1.10; 1.31]	47.5%	9.7%
Karlen, 1999	-0.3567	0.4318	0.70	[0.30; 1.63]	0.5%	2.9%
Loo, 2019	0.0000	0.1678	1.00	[0.72; 1.39]	3.3%	7.3%
Meyers, 2020	0.3853	0.1437	1.47	[1.11; 1.95]	4.6%	7.9%
Taborelli, 2020	-0.0305	0.2196	0.97	[0.63; 1.49]	2.0%	6.2%
van den Heuvel, 2016	0.3148	0.2202	1.37	[0.89; 2.11]	1.9%	6.2%
Wilson, 2016	0.1989	0.1901	1.22	[0.84; 1.77]	2.6%	6.8%
Winther, 2004	-0.3011	0.6677	0.74	[0.20; 2.74]	0.2%	1.5%
Common effect model			**1.14**	**[1.07; 1.22]**	**83.3%**	**--**
Random effects model			**1.15**	**[1.00; 1.31]**	**--**	**81.7%**
Heterogeneity: $I^2 = 54\%$, $\tau^2 = 0.0256$, $p = 0.01$						
ethnicity = non-W						
Jung, 2017	1.2442	0.2663	3.47	[2.06; 5.85]	1.3%	5.2%
Na, 2022	-0.0513	0.0804	0.95	[0.81; 1.11]	14.6%	9.2%
So, 2017	0.9042	0.3516	2.47	[1.24; 4.92]	0.8%	3.9%
Common effect model			**1.10**	**[0.95; 1.28]**	**16.7%**	**--**
Random effects model			**1.93**	**[0.86; 4.33]**	**--**	**18.3%**
Heterogeneity: $I^2 = 93\%$, $\tau^2 = 0.4476$, $p < 0.01$						
**Common effect model**			**1.13**	**[1.07; 1.20]**	**100.0%**	**--**
**Random effects model**			**1.23**	**[1.03; 1.46]**	**--**	**100.0%**

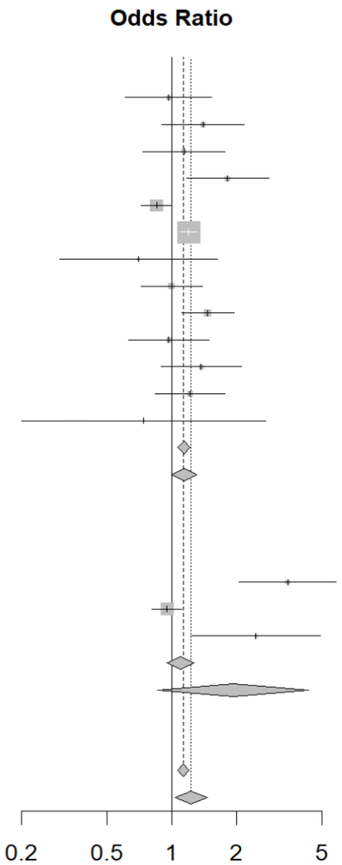

Heterogeneity: $I^2 = 72\%$, $\tau^2 = 0.0781$, $p < 0.01$
Test for subgroup differences (common effect): $\chi^2_1 = 0.19$, df = 1 ($p = 0.66$)
Test for subgroup differences (random effects): $\chi^2_1 = 1.56$, df = 1 ($p = 0.21$)
