# Supplementary material for: Risk factors for prostate cancer: An umbrella review of prospective observational studies and mendelian randomization analyses
Source: PLoS Med. 2024 Mar 15;21(3):e1004362. doi: 10.1371/journal.pmed.1004362 (PMC10980219; doi:10.1371/journal.pmed.1004362)
Supplement: S4 Table — NA: not available; PI, prediction interval; PA, physical activity; CRP, C-reactive protein; T2D, type 2 diabetes; BPH, benign prostate hyperplasia; UC, ulcerative colitis; HIV, human immunodeficiency virus; AIDS, acquired immune deficiency syndrome. (DOCX) [file pmed.1004362.s009.docx]

| S4 Table. Details of evidence grading for significant associations from meta-analyses. | | | | | | | | | | | | | |
| --- | --- | --- | --- | --- | --- | --- | --- | --- | --- | --- | --- | --- | --- |
| Factors | cases | P-value | 95%PI | I^2^ (%) | Egger’s P value | P | 95%PI | Heterogeneity | Cases | Egger’s P value | largest weight  yes=Y  no=N | Evidence Convincing=1; highly suggestive=2; suggestive=3; weak=4 | Reason:  H=heterogeneity; B=bias; N=number of cases; P=P value; L=large |
| Increased risk | | | | | | | | | | | | | |
| sweetened beverage | 1899 | 0.001 | 1.0-1.39 | 0 | 0.989 | <0.001 | excluding 1 | <50% | >1000 | >0.1 | Y | 2 | P |
| circulating 25-hydroxyvitamin D | 11380 | 0.001 | 1.07-1.28 | 20.6 | 0.904 | <0.001 | excluding 1 | <50% | >1000 | >0.1 | N | 2 | P&L |
| asbestos | 15687 | 4.47E-05 | 1.00-1.28 | 29.7 | 0.217 | <0.001 | excluding 1 | <50% | >1000 | >0.1 | Y | 2 | P |
| CRP | 3132 | 0.002 | 1.02-1.16 | 0 | 0.69 | <0.05 | excluding 1 | <50% | >1000 | >0.1 | Y | 3 | P |
| calcium | 33127 | 0.003 | 0.99-1.06 | 30.3 | 0.288 | <0.05 | including 1 | <50% | >1000 | >0.1 | Y | 3 | P |
| finasteride | 3552 | 1.75E-32 | 1.53-2.88 | 50.8 | 0.476 | <0.000001 | excluding 1 | >50% | >1000 | >0.1 | Y | 3 | H |
| acne in adolescence | 3823 | 0.001 | 0.32-7.25 | 0 | 0.338 | <0.001 | including 1 | <50% | >1000 | >0.1 | Y | 3 | P & PI |
| infertility | 4199 | 0.021 | 0.34-6.36 | 83.7 | 0.93 | <0.05 | including 1 | >50% | >1000 | >0.1 | Y | 3 | H |
| prostatitis | 3901 | 0.004 | 0.01-184.18 | 75.9 | 0.122 | <0.05 | including 1 | >50% | >1000 | >0.1 | Y | 3 | H |
| BPH | >10000 | 0.048 | 0.47-4.22 | 84.5 | 0.218 | <0.05 | including 1 | >50% | >1000 | >0.1 | N | 3 | H |
| number of female partners | 7414 | 0.001 | 0.73-2.67 | 61.3 | 0.728 | <0.001 | including 1 | >50% | >1000 | >0.1 | N | 3 | P&H |
| melanoma | 10544 | 7.06E-17 | 1.08-1.42 | 75 | 0.571 | <0.000001 | excluding 1 | >50% | >1000 | >0.1 | Y | 3 | H |
| serum folate | 4443 | 0.008 | 0.96-1.52 | 0 | 0.428 | <0.05 | including 1 | <50% | >1000 | >0.1 | N | 3 | P |
| adult weight gain | >1000 | 0.033 | 0.86-1.54 | 0 | 0.605 | <0.05 | including 1 | <50% | >1000 | >0.1 | N | 3 | P |
| firefighter | >1000 | 3.196E-5 | 0.92-1.60 | 80.7 | 0.417 | <0.001 | including 1 | >50% | >1000 | >0.1 | N | 3 | P&H |
| total dairy products | 110982 | 0.039 | 0.98-1.10 | 39 | 0.089 | <0.05 | including 1 | <50% | >1000 | <0.1 | N | 3 | B |
| vasectomy | 80354 | 7.90E-5 | NA | 64.2 | 0.654 | <0.001 | NA | >50% | >1000 | >0.1 | - | 3 | H |
| height | 11791 | 3.43E-11 | 1.02-1.16 | 24 | 0.012 | <0.000001 | excluding 1 | <50% | >1000 | <0.1 | Y | 3 | B |
| cobalt | >1000 | 0.001 | 0.90-1.31 | 71.2 | 0.975 | <0.001 | including 1 | >50% | >1000 | >0.1 | Y | 3 | P&H |
| processed meat | 29644 | 0.006 | 1.01-1.11 | 0 | 0.085 | <0.05 | excluding 1 | <50% | >1000 | <0.1 | Y | 3 | P&H |
| birthweight | 8059 | 0.045 | NA | 13.2 | 0.202 | <0.05 | - | <50% | >1000 | >0.1 | N | 3 | P |
| egg consumption | 609 | 0.043 | 0.38-5.7 | 39.6 | 0.042 | <0.05 | including 1 | <50% | <1000 | <0.1 | N | 4 | N&B |
| androgenic alopecia | 737 | 0.038 | 0.40-5.03 | 51.9 | 0.552 | <0.05 | including 1 | >50% | <1000 | >0.1 | Y | 4 | N |
| UC | >1000 | 0.009 | 0.66-2.31 | 71.9 | 0.382 | <0.05 | including 1 | >50% | >1000 | >0.1 | Y | 3 | P&H |
| first degree family breast cancer | 8025 | 7.21E-09 | 1.11-1.36 | 53.7 | 0.066 | <0.000001 | excluding 1 | >50% | >1000 | <0.1 | Y | 4 | H&B |
| total cholesterol level for high-grade | >1000 | 0.001 | 1.07-1.33 | 47.1 | 0.39 | <0.001 | excluding 1 | <50% | >1000 | >0.1 | N | 2 | L |
| primary Sjögren's syndrome | <1000 | 0.038 | 0.12-18.68 | 0 | NA | <0.05 | including 1 | <50% | <1000 | NA | Y | 4 | N |
| tissue level linoleic acid | 5155 | 0.019 | 0.50-1.30 | 53.5 | NA | <0.05 | including 1 | >50% | >1000 | NA | N | 3 | H |
| total flavonoids | 14571 | 0.02 | 0.98-1.26 | 0 | NA | <0.05 | including 1 | <50% | >1000 | NA | Y | 3 | P |
| Decreased risk | | | | | | | | | | | | | |
| occupational PA | 1684 | 1.70E-04 | 0.76-0.99 | 0 | 0.361 | <0.001 | excluding 1 | <50% | >1000 | >0.1 | Y | 2 | - |
| smoking | >50000 | 4.614E-15 | 0.46-1.17 | 90.5 | 0.001 | <0.000001 | including 1 | >50% | >1000 | <0.1 | Y | 3 | H&B |
| age at first intercourse | 7558 | 0.036 | 0.60-1.21 | 27.3 | 0.475 | <0.05 | including 1 | <50% | >1000 | >0.1 | N | 3 | P |
| regular use of aspirin | 81485 | 0.002 | NA | 83 | 0.103 | <0.05 | - | >50% | >1000 | >0.1 | Y | 3 | P&H |
| coffee | 57732 | 0.013 | 0.73-1.13 | 53.2 | 0.409 | <0.05 | including 1 | >50% | >1000 | >0.1 | N | 3 | H |
| soy consumption | 6866 | 0.022 | 0.79-1.01 | 0 | 0.06 | <0.05 | including 1 | <50% | >1000 | <0.1 | N | 3 | B |
| digoxin | 14223 | 0.044 | 0.99-1.06 | 47.8 | 0.075 | <0.05 | including 1 | <50% | >1000 | <0.1 | Y | 3 | P&B |
| fat mass | 7205 | 0.045 | 0.17-4.43 | 62 | 0.117 | <0.05 | including 1 | >50% | >1000 | >0.1 | Y | 3 | P&H |
| T2D | 118077 | 5.95E-11 | 0.56-1.34 | 80.7 | 0.487 | <0.000001 | including 1 | >50% | >1000 | >0.1 | Y | 3 | H |
| blood alpha-tocopherol level | 4004 | 0.001 | 0.62-1.01 | 12.5 | 0.083 | <0.001 | including 1 | <50% | >1000 | <0.1 | N | 3 | B |
| Parkinson's disease | >10000 | 0.016 | 0.46-1.32 | 95.2 | 0.157 | <0.05 | including 1 | >50% | >1000 | >0.1 | N | 3 | H |
| daidzein | 1452 | 0.01 | 0.46-1.21 | 0 | 0.438 | <0.05 | including 1 | <50% | >1000 | >0.1 | N | 3 | P |
| HIV/AIDS | 2531 | 0.004 | 0.30-1.91 | 92 | 0.228 | <0.05 | including 1 | >50% | >1000 | >0.1 | Y | 3 | P&H |
| finasteride | 17093 | 0.028 | 0.24-2.08 | 98.7 | 0.305 | <0.05 | including 1 | >50% | >1000 | >0.1 | Y | 3 | P&H |
| selenium | 3297 | 0.047 | 0.19-2.32 | 74.8 | 0.38 | <0.05 | including 1 | >50% | >1000 | >0.1 | Y | 3 | H |
| schizophrenia | 1706 | 6.58E-06 | 0.31-1.14 | 87.7 | 0.383 | <0.001 | including 1 | >50% | >1000 | >0.1 | Y | 3 | H |

Abbreviations: NA: not available; PI, prediction interval; PA, physical activity; CRP, C-reactive protein; T2D, type 2 diabetes; BPH, benign prostate hyperplasia; UC, ulcerative colitis; HIV, human immunodeficiency virus; AIDS, acquired immune deficiency syndrome.
