# Supplementary material for: Risk factors for prostate cancer: An umbrella review of prospective observational studies and mendelian randomization analyses
Source: PLoS Med. 2024 Mar 15;21(3):e1004362. doi: 10.1371/journal.pmed.1004362 (PMC10980219; doi:10.1371/journal.pmed.1004362)
Supplement: S5 Table — The statistical test to determine the P value in meta-analyses was using the random-effects inverse-variance model with DerSimonian—Laird method. Metrics with * denoting advanced, aggressive, high-grade, or lethal prostate cancer, metrics with # denoting nonadvanced, nonaggressive, or localized prostate cancer. W, White; A, Asian; RR, risk ratio; OR, odds ratio; HR, hazard ratio; SIR, standard incidence ratio; SRRE, summary relative risk estimate; NR, not reported; NA, not available; PA, physical activity; DHA, docosahexaenoic acids; EPA, eicosapentaenoic; HDL, high-density lipoprotein; LDL, low-density lipoprotein; CRP, C-reactive protein; T2D, type 2 diabetes; BPH, benign prostate hyperplasia; HIV, human immunodeficiency virus; AIDS, acquired immune deficiency syndrome; CD, Crohn’s disease; UC, ulcerative colitis; AASVs, anti-neutrophil cytoplasm antibody associated vasculitides; ACEI, angiotensin converting enzyme inhibitors; NSAID, nonsteroidal anti-inflammatory drug; CCB, calcium channel blockers. (DOCX) [file pmed.1004362.s010.docx]

| S5 Table. Basic characteristics of included meta-analyses and evidence grading results | | | | | | | | | | | | |  |
| --- | --- | --- | --- | --- | --- | --- | --- | --- | --- | --- | --- | --- | --- |
| Author, year | **Factors** | **Number of prostate cancer cases** | **Total number of participants** | **Study population** | **Number of datasets** | **Type of metric** | **Comparison** | **Summary of metric** | **P value** | **Heterogeneity (I^2^, %)** | **Egger**  **P value** | **AMSTAR-2 total score** | **Evidence**  **grading** |
| *LIFESTYLE (N=17)* | | | | | | | | | | | | |  |
| Yang, 2023  (37316851) | ever smoking | NR >50000 | >3 000 000 |  | 35 | RR | ever smoking vs never-smoker | 0.96(0.93, 1.004) | 0.074 | 67 | 0.959 | 14.5 | Null |
|  | current smoking | NR >50000 | >3 000 000 | W and A (n=4) | 27 | RR | current smoking vs non-smoker (never smokers plus former  smokers) | 0.74(0.68, 0.80) | 4.614E-15 | 90.5 | 0.001 |  | Suggestive |
|  | former smoking | NR >50000 | >3 000 000 |  | 31 | RR | Former smoking vs never smoker | 0.98(0.95, 1.02) | 0.313 | 61.5 | NR |  | Null |
| Liu, 2020  (32664160) | sleep duration | 716 | 270403 | W and A (n=1) | 6 | RR | short sleep vs reference 7/8h or 7-8h | 0.99(0.91,1.07) | 0.737 | 0 | 0.46 | 12 | Null |
|  |  | 678 | 284503 | W and A (n=1) | 6 | RR | long sleep vs reference 7/8h or 7-8h | 0.88(0.75,1.04) | 0.145 | 56.2 | 0.13 |  | Null |
| Berger, 2016  (31362941) | sedentary behavior | 32060 | 610457 | W | 11 | RR | highest vs lowest | 1.07(0.99,1.16) | 0.096 | 63.1 | 0.106 | 13 | Null |
|  |  | 5000 | 434881 | W | 6 | RR | highest vs lowest | 1.02(0.93,1.12)^*^ | 0.7 | 16.9 | 0.053 |  | Null |
| Benke, 2018  (29788165) | overall PA | 90812 | 2434025 | W and A (n=4) | 27 | RR | highest versus the lowest level of overall PA | 0.99(0.94,1.05) | 0.728 | 71.7 | 0.071 | 14 | Null |
| Guo, 2017  (28353571) | green tea | 996 | NA | A | 4 | RR | one cup increase | 0.99(0.96,1.03) | 0.662 | 0 | 0.235 | 14 | Null |
|  |  | 996 | NA | A | 4 | RR | highest vs lowest | 0.98(0.80,1.19) | 0.821 | 0 | 0.82 |  | Null |
| Lin, 2014  (24528523) | black tea | 1580 | NA | W and A | 4 | RR | highest vs lowest | 1.02(0.80,1.31) | 0.882 | 57.1 | 0.886 | 13 | Null |
| Chen, 2021  (33431520) | coffee | 57732 | 1081586 | W and A (n=2) | 15 | RR | highest vs lowest | 0.91(0.84,0.98) | 0.013 | 53.2 | 0.409 | 14 | Suggestive |
|  |  | NA | 547981 | W and A | 6 | RR | highest vs lowest | 0.92(0.83,1.02)^#^ | 0.125 | 37 | 0.454 |  | Null |
|  |  | NA | 609114 | W and A | 8 | RR | highest vs lowest | 0.88(0.71,1.09)^*^ | 0.24 | 52.7 | 0.186 |  | Null |
| Hong, 2020  (32717903) | alcohol | 18680 | 510640 | W and A | 6 | RR | per 14g/day increase | 1.00(0.97,1.03)^#^ | 0.93 | 56.4 | 0.046 | 14 | Null |
|  |  | 2829 | 569560 | W and A | 8 | RR | per 14g/day increase | 1.00(0.96,1.04)^*^ | 0.96 | 34.9 | 0.16 |  | Null |
| Jian, 2018  (30122473) | number of female sexual partners | 7414 | 16033 | W and A | 15 | OR | highest vs lowest | 1.40 (1.14,1.70) | 0.001 | 61.3 | 0.728 | 13 | Suggestive |
|  | age at first intercourse | 7558 | 16374 | W and A | 13 | OR | highest vs lowest | 0.85(0.74,0.99)^*^ | 0.036 | 27.3 | 0.475 |  | Suggestive |
|  | ejaculation frequency | NA | NA | W and A | 14 | OR | highest vs lowest | 0.97(0.87,1.08) | NA | 85.5 | NA |  | Null |
| Rivera, 2020  (32093096) | shiftwork | NA | 2508328 | W and A | 11 | RR | not reported | 1.03(0.96,1.10) | NA | 18.9 | NA | 13.5 | Null |
| Krstev, 2019  (31360689) | occupational PA | 1684 | NA | W | 10 | RR | higher vs lower | 0.87(0.80,0.94) | 0.00017 | 0 | 0.361 | 8 | Highly suggestive |
|  | whole body vibration | 4768 | NA | W | 7 | RR | dose response | 1.01(0.98,1.05) | 0.549 | 69.1 | 0.487 |  | Null |
|  | farming | 65448 |  | W | 15 | RR | exposed vs unexposed | 0.97(0.94,1.00) | 0.087 | 84.5 | 0.608 |  | Null |
| Sritharan, 2017  (29149887) | police | 1273 | 1191880 | W | 7 | RR | exposed vs unexposed | 1.10(0.96,1.26) | 0.161 | 37 | 0.604 | 14 | Null |
| Debono, 2023  (37389311) | firefighter | >1000 | NA | W and A (n=1) | 15 | SIR/HR/RR | ever-employment as a career firefighter vs general population | 1.21(1.11,1.33) | 3.196E-05 | 80.7 | 0.417 | 12 | Suggestive |
| *DIET & NUTRITION (N=44)* | | | | | | | | | | | | |  |
| Luo, 2021  (34017849) | total tomato | 9859 | NA | W and A | 9 | RR | highest vs lowest | 0.91(0.79,1.03) | 0.138 | 61.6 | 0.5 | 13.5 | Null |
| Rowles, 2018  (29317772) | raw tomato | 7544 | 278339 | W and A | 5 | RR | highest vs lowest | 1.00(0.87,1.15) | 0.966 | 56.9 | 0.477 | 14 | Null |
| Llaha, 2021  (33557387) | sweetened beverage | 1899 | NA | W | 5 | RR | highest vs lowest | 1.18(1.07,1.31) | 0.001 | 0 | 0.989 | 14.5 | Highly suggestive |
| Cheng, 2019  (31277188) | Mediterranean diet | 30985 | 398067 | W | 5 | RR | adhered vs non-adhered | 0.96(0.92,1.00) | 0.057 | 0 | 0.904 | 14 | Null |
|  |  | 3321 | 355958 | W | 3 | RR | adhered vs non-adhered | 0.98(0.89,1.09)^*^ | 0.718 | 0 | 0.717 |  | Null |
| Aune, 2015  (25527754) | total calcium intake | 33127 | 750275 | W and A (n=1) | 9 | RR | per 400mg/d | 1.02(1.01,1.04) | 0.003 | 30.3 | 0.288 | 14 | Suggestive |
|  |  | 4174-4373 | 532402 | W | 7 | RR | per 400mg/d | 1.03(0.99,1.07)^*^ | 0.127 | 43.5 | 0.759 |  | Null |
|  |  | 17533-17732 | 467081 | W | 6 | RR | per 400mg/d | 1.01(0.98,1.03)^#^ | 0.651 | 28.7 | 0.037 |  | Null |
| Zhao, 2023  (35945656) | total dairy products | 110982 | 1536556 | W and A (n=1) | 26 | RR | highest vs lowest | 1.05(1.00,1.09) | 0.039 | 39 | 0.089 | 13.5 | Suggestive |
|  | total milk | 32690 | NR |  | 17 | RR | highest vs lowest | 1.07(1.00,1.14) |  | 46 |  |  | Null |
|  | cheese | 33236 | NR |  | 15 | RR | highest vs lowest | 1.03(0.99,1.08) |  | 0 |  |  | Null |
|  | butter | 2943 | NR |  | 5 | RR | highest vs lowest | 1.08(1.03,1.12) |  | 0 | NA |  |  |
|  | yogurt | 8802 | NR |  | 7 | RR | highest vs lowest | 1.14(0.98,1.32) |  | 65 | NA |  | Null |
|  | ice cream | 5268 | NR |  | 5 | RR | highest vs lowest | 0.94(0.87,1.01) |  | 0 | NA |  | Null |
| Keum, 2015  (26293984) | egg consumption | 3655 | NA | W and A | 6 | RR | increase of 5 eggs | 1.00(0.88,1.14) | 0.989 | 0 | 0.761 | 13.5 | Null |
|  |  | 609 | NA | W and A | 4 | RR | increase of 5 eggs | 1.48(1.01,2.15)^*^ | 0.043 | 39.6 | 0.042 |  | Weak |
| Wang, 2014  (25543518) | dietary folate intake | 12898 | 192702 | W | 5 | RR | highest vs lowest | 1.02(0.95,1.09) | 0.598 | 0 | 0.513 | 13 | Null |
|  |  | 12898 | 192702 | W | 5 | RR | per 100 ug/day increment | 1.01(0.99,1.02) | 0.433 | 0 | 0.694 |  | Null |
| Applegate, 2018  (29300347) | soy consumption | 6866 | 199688 | W and A (n=2) | 7 | RR | highest vs lowest | 0.90(0.82,0.99) | 0.022 | 0 | 0.06 | 13 | Suggestive |
| Xu, 2014  (24838848) | circulating 25-hydroxyvitamin D | 11380 | 25036 | W | 17 | OR | highest vs lowest | 1.18(1.07,1.30) | 0.001 | 20.6 | 0.904 | 14 | Highly suggestive |
| Sayehmiri, 2018  (29936712) | selenium | 3297 | 66682 | W | 5 | RR | not reported | 0.67(0.45,0.99) | 0.047 | 74.8 | 0.38 | 13 | Suggestive |
| Nouri-Majd, 2022  (35198587) | red meat | NA | NA | W and A (n=1) | 14 | RR | highest vs lowest | 1.05(0.98,1.12) | NR | 40.5 | NR | 14.5 | Null |
|  | processed meat | 29644 | 587221 | W | 13 | RR | highest vs lowest | 1.06(1.02,1.10) | 0.006 | 0 | 0.085 |  | Suggestive |
| Mahmoud, 2016  (27824905) | zinc | 5828 | 92952 | W | 4 | RR | highest vs lowest | 1.06(0.93,1.21) | NA | 0 | NA | 14 | Null |
| Xu, 2015  (26186528) | total fat intake | 9367 | 322020 | W | 11 | RR | every 28.35 g increment a day | 1.00(0.98,1.01) | 0.585 | 10.2 | 0.052 | 12.5 | Null |
|  |  | NA | 498489 | W | 5 | RR | every 28.35 g increment a day | 1.02(0.96,1.08) | 0.628 | 47.9 | 0.867 |  | Null |
| Farrell, 2021  (33530576) | DHA | NA | NA | W | 10 | HR | per 1% increment | 1.02(1.00,1.04) | 0.275 | 36 | 0.048 | - | Null |
|  | EPA | NA | NA | W | 10 | HR | per 1% increment | 1.03(0.98,1.08) | 0.2 | 0 | 0.869 |  | Null |
| Alexander, 2015  (25826711) | dietary omega-3 | 14573 | 446243 | W | 13 | SRRE | highest vs lowest | 1.00(0.93,1.09) | NA | 50.4 | NA | 11 | Null |
| Rienks, 2017  (28969363) | daidzein | 1412 | 3320 | W and A (n=2) | 4 | RR | highest vs lowest | 0.75(0.60,0.93) | 0.01 | 0 | 0.438 | 15 | Suggestive |
|  | genistein | 2065 | 4633 | W and A | 4 | RR | highest vs lowest | 0.89(0.73,1.08) | 0.25 | 0 | 0.1677 |  | Null |
|  | equol | 1452 | 3451 | W and A | 4 | RR | highest vs lowest | 0.83(0.54,1.27) | 0.393 | 64.6 | 0.31 |  | Null |
| Zhu, 2020  (31554922) | dietary inflammatory index | 2825 | 46703 | W | 2 | RR | highest vs lowest | 1.37(0.73,2.57) | 0.329 | 66.5 | NA | 13.5 | Null |
|  |  | 2825 | 46703 | W | 2 | RR | 1-unit increase | 1.02(0.98,1.05) | 0.41 | 61.3 | NA |  | Null |
| Rowles, 2017  (28440323) | dietary lycopene | 15481 | 249853 | W | 6 | RR | highest vs lowest | 0.93(0.86,1.01) | 0.08 | 11.6 | 0.617 | 13.5 | Null |
| Lv, 2022  (34617559) | dietary phosphorus intake | 6130 | 54727 | W | 4 | OR | high vs low | 1.12(0.97,1.30) | 0.115 | 20 | 0.475 | - | Null |
| Yousefi, 2023  (37077161) | dietary linoleic acid | 34838 | 496864 | W | 9 | RR | highest vs lowest | 1.00(0.97,1.04) | NR | 31.9 | NA | 14 | Null |
| Balali, 2023  (37160404) | total nut intake | 31042 | 242226 | W | 4 | RR | highest vs lowest | 1.00(0.95,1.05) | NR | 0 | NA | 13.5 | Null |
| Yan, 2022  (34286657) | fruit | NA | NA | W and A (n=2) | 14 | RR | highest vs lowest | 1.00(0.94,1.05) |  |  |  | 13 | Null |
|  | vegetable | NA | NA | W and A (n=4) | 15 | RR | highest vs lowest | 0.98(0.94,1.02) |  |  |  |  | Null |
| Parra-Soto, 2022  (35655214) | vegetarian | 499 | 509027 | W | 4 | RR | vegetarian vs meat-eater | 0.83(0.63,1.08) | NA | 73 | NA | 14 | Null |
|  | pescatarian | 250 | 532243 | W | 4 | RR | pescatarian vs meat-eater | 0.97(0.76,1.23) | NA | 41 | NA |  | Null |
| Loh, 2022  (36615673) | dietary vitamin E intake | 19634 | 498431 | W | 6 | RR | NR | 0.99(0.93,1.05) | NA | 12.48 | NA | 15 | Null |
|  | supplemental vitamin E intake | 25043 | 605829 | W | 9 | RR | NR | 1.00(0.95,1.04) | NA | 0 | NA |  | Null |
| Liu, 2022  (35545772) | total flavonoids | 14571 | 218842 | W | 6 | OR | highest vs lowest | 1.11(1.02,1.22) | 0.02 | 0 | NA | 14.5 | Suggestive |
| Alzahrani, 2022  (35820576) | total protein intake | 12567 | 214276 | W and A (n=1) | 8 | RR | highest vs lowest | 0.99(0.92,1.07) |  | 12.8 | NA | 12 | Null |
|  | animal protein intake | 26207 | 324197 | W | 6 | RR | highest vs lowest | 0.99(0.95,1.04) |  | 0 | NA |  | Null |
|  | plant protein intake | 26137 | 329082 | W | 6 | RR | highest vs lowest | 1.01(0.96,1.06) |  | 0 | NA |  | Null |
|  | dairy protein intake | 9864 | 167489 | W | 4 | RR | highest vs lowest | 1.08(0.97,1.21) | 0.152 | 38.1 | NA |  | Null |
| Long, 2023  (37343525) | cruciferous vegetable intake |  |  | W and A (n=1) | 9 | RR | Highest vs lowest | 0.98(0.93,1.02) | 0.076 | 43.8 | NA | 15 | Null |
| Eshaghian, 2023  (37593679) | total fish | 27197 | 662505 | W and A (n=2) | 15 | RR | highest vs lowest | 0.97(0.86,1.10) | 0.63 | 78.3 | 0.92 | 14.5 | Null |
|  | total fish | 3098 | 190284 | W and A (n=1) | 6 | RR | highest vs lowest | 1.01(0.91,1.13)^*^ | 0.84 | 0 | NA |  | Null |
| *ANTHROPOMETRIC INDICES (N=5)* | | | | | | | | | | | | |  |
| Harrison, 2020  (32162172) | BMI | 5915 | 36815 | W | 15 | OR | per 5 increase | 1.00(0.89,1.12 ) | 0.99 | 75.2 | 0.651 | 13 | Null |
|  |  | 1747 | 6064 | W | 3 | OR | per 5 increase | 0.98(0.82,1.18)^*^ | 0.858 | 46.1 | 0.942 |  | Null |
|  |  | 11468 | 14324 | W | 3 | OR | overweight vs normal | 1.07(0.84,1.37) | 0.584 | 0 | 0.449 |  | Null |
|  |  | 5482 | 8931 | W | 5 | OR | obese vs normal | 0.87(0.71,1.06) | 0.173 | 0 | 0.64 |  | Null |
| Chen, 2016  (26356247) | adult weight gain | 19103 | 470688 | W | 5 | RR | highest vs lowest | 1.00(0.91,1.10) | 0.968 | 36.9 | 0.273 | 13.5 | Null |
|  |  | NA | 422907 | W | 4 | RR | highest vs lowest | 1.15(1.01,1.32)^*^ | 0.033 | 0 | 0.605 |  | Suggestive |
| Zuccolo, 2008  (18768501) | height | 11791 | NA | W and A (n=1) | 31 | RR | per 10 cm increase | 1.09(1.06,1.12) | 3.43E-11 | 24 | 0.012 | 12 | Suggestive |
|  |  | NA | NA | W | 13 | RR | per 10 cm increase | 1.12(1.05,1.19)^*^ | NA | 47.3 | NA |  | - |
| Purcell, 2021  (34918023) | fat mass | 7205 | 259843 | W | 3 | HR | highest vs lowest quintiles | 0.87(0.76,1.00) | 0.045 | 62 | 0.117 | 12.5 | Suggestive |
| Zhou, 2016  (26930450) | birth weight | 8059 | 164362 | W | 13 | RR | per kg increase | 1.02(1.00,1.05) | 0.045 | 13.2 | 0.202 | 14 | Suggestive |
|  |  | NA | 58690 | W | 8 | RR | per kg increase | 1.08(0.99,1.18)^*^ | 0.076 | 39.5 | 0.054 |  | Null |
| *BIOMARKERS (N=12)* | | | | | | | | | | | | |  |
| Cui, 2014  (24667740) | blood α-tocopherol level | 4004 | 369718 | W | 9 | RR | highest vs lowest | 0.79(0.68,0.91) | 0.001 | 12.5 | 0.083 | 12.5 | Suggestive |
|  | blood γ-tocopherol level | 4004 | 369718 | W | 9 | RR | highest vs lowest | 0.90(0.71,1.12) | 0.322 | 61.1 | 0.083 |  | Null |
| Wang, 2014  (25543518) | serum folate | 4443 | 9810 | W | 5 | RR | highest vs lowest | 1.21(1.05,1.39) | 0.008 | 0 | 0.428 | 13 | Suggestive |
|  |  | 4443 | 9810 | W | 5 | RR | per 5 nmol/L increment | 1.04(1.00,1.07) | 0.042 | 0 | 0.181 |  | - |
| Liu, 2015  (25953767) | HDL | 8553 | 332381 | W | 6 | RR | highest vs lowest | 0.93(0.80,1.10) | 0.4 | 61 | 0.59 | 13.5 | Null |
|  |  | NA | 29093 | W | 3 | RR | highest vs lowest | 1.21(0.63,2.30)^*^ | 0.57 | 85.6 | 0.58 |  | Null |
|  | LDL | 5825 | 273924 | W | 4 | RR | highest vs lowest | 1.17(0.88,1.55) | 0.29 | 63.83 | 0.61 |  | Null |
|  |  | 330 | 70116 | W | 2 | RR | highest vs lowest | 1.30(0.35,4.80)^*^ | 0.69 | 88.4 | NA |  | Null |
| Burton, 2021  (33431998) | leptin | 4014 | 10070 | W and A | 9 | OR | per 2.5 µg/ml increase | 0.97(0.93,1.01) | 0.156 | 57.2 | 0.872 | 15 | Null |
|  |  | 1190 | 6329 | W and A | 7 | OR | per 2.5 µg/ml increase | 1.02(0.99,1.05)^*^ | 0.185 | 0 | 0.176 |  | Null |
|  | adiponectin | 1062 | 6154 | W and A | 7 | OR | per 2.5 µg/ml increase | 0.98(0.93,1.04) | 0.576 | 55.5 | 0.088 |  | Null |
|  |  | 861 | 3233 | W and A | 7 | OR | per 2.5 µg/ml increase | 0.97(0.92,1.03)^*^ | 0.387 | 29.7 | 0.81 |  | Null |
| Guo, 2018  (30075605) | serum C-peptide concentration | 2706 | 5350 | W | 6 | OR | highest vs lowest | 0.95(0.69,1.31) | 0.76 | 68.4 | 0.696 | 9 | Null |
| Michels, 2021  (33264718) | CRP | 3132 | 96284 | W | 8 | HR | highest vs lowest quartiles | 1.09(1.03,1.15) | 0.002 | 0 | 0.69 | 14.5 | Suggestive |
|  | white blood cell count | 803 | NA |  | 4 | HR | highest vs lowest quartiles | 1.14(0.91,1.43) | 0.25 | 85 | NA |  | Null |
| Yousefi, 2023  (37077161) | tissue level linoleic acid | 5155 | 14758 | W | 9 | RR | highest vs lowest | 0.81(0.67,0.97) | 0.019 | 53.5 | NA | 14 | Suggestive |
| Liu, 2023  (37306155) | total cholesterol level | >1000 |  | W | 10 | RR | highest vs lowest | 1.26(1.09,1.46)^*^ | 0.001 | 47.1 | 0.390 | 11 | Highly suggestive |
| *CLINICAL VARIABLES, DISEASES, AND TREATMENTS (N=39)* | | | | | | | | | | | | |  |
| Sun, 2021  (32801354) | HIV/AIDS | 2531 | >377767 | W | 22 | SIR | patients vs non-patients | 0.74(0.60,0.91) | 0.004 | 92 | 0.228 | 14.5 | Suggestive |
| Ma, 2021  (34035396) | hepatitis C | 9924 | 5454338 | W | 5 | RR | patients vs non-patients | 0.74(0.44,1.22) | 0.233 | 95.1 | 0.614 | 13 | Null |
| Gang,2015  (25105463) | T2D | 118077 | >7383131 | W | 31 | HR | patients vs non-patients | 0.84(0.79,0.90) | 5.95E-08 | 80.3 | 0.487 | 15 | Suggestive |
| Wei, 2021  (33247563) | periodontitis | 409 | 34738 | W | 4 | RR | patients vs non-patients | 1.18(0.98,1.42) | 0.08 | 34.7 | 0.2 | 14 | Null |
| Zhang, 2020  (31892984) | prostatitis | 3901 | 102602 | W | 3 | RR | patients vs non-patients | 1.45(1.13,1.87) | 0.004 | 75.9 | 0.122 | 13 | Suggestive |
| Zhu, 2016  (27767045) | asthma | 15913 | 1681562 | W and A | 13 | RR | patients vs non-patients | 1.05(0.92,1.20) | 0.465 | 93.2 | 0.897 | 13 | Null |
| Behboudi, 2021  (34722244) | infertility | 4199 | 2089718 | W | 5 | OR | infertile vs fertile | 1.49(1.06,2.09) | 0.021 | 83.7 | 0.93 | 15 | Suggestive |
| Dai, 2016  (27149447) | BPH | >10000 | 969640 | W | 6 | RR | patients vs non-patients | 1.41(1.00,1.99) | 0.048 | 84.5 | 0.218 | 14 | Suggestive |
| Zhou, 2023  (37310514) | CD | <1000 |  | W and A | 12 | HR | patients vs non-patients | 1.03(0.91,1.17) | 0.65 | 10 | NR | 14 | Null |
|  | UC | >1000 | >100000 | W and A (n=3) | 16 | HR | patients vs non-patients | 1.22(1.05,1.41) | 0.009 | 71.9 | 0.382 |  | Suggestive |
| Ren, 2019  (31477094) | first degree family breast cancer | 8025 | 16925350 | W | 5 | RR | patients vs non-patients | 1.19(1.12,1.26) | 7.21E-09 | 53.7 | 0.066 | 13 | Weak |
| Acharya, 2020  (32175697) | melanoma | 10544 | 285938 | W | 16 | SIR | patients vs non-patients | 1.24(1.18,1.30) | 7.06E-17 | 75 | 0.571 | 14 | Suggestive |
| Zhang, 2018  (30403728) | acne in adolescence | 3823 | 289048 | W | 3 | OR | patients vs non-patients | 1.51(1.19,1.93) | 0.001 | 0 | 0.338 | 10 | Suggestive |
| Ge, 2022  (35303584) | schizophrenia | 1706 | 146338 | W and A | 8 | SIR | patients vs non-patients | 0.59(0.46,0.74) | 6.575E-06 | 87.7 | 0.383 | 15 | Suggestive |
| Chen, 2017  (29044216) | Parkinson's disease | >10000 | >9232692 | W | 8 | RR | patients vs non-patients | 0.78(0.64,0.96) | 0.016 | 95.2 | 0.157 | 13.5 | Suggestive |
| Simon, 2015  (26271620) | rheumatoid arthritis | 1936 | NA | W | 17 | SIR | patients vs non-patients | 1.15(0.98,1.34) | 0.078 | 92.1 | 0.533 | 9.5 | Null |
| Shang, 2015  (25973882) | AASVs | 20 | NA | W | 4 | SIR | patients vs non-patients | 1.44(0.88,2.34) | 0.145 | 0.2 | 0.998 | 13 | Null |
| Liang, 2018  (29529997) | androgenic alopecia | 4998 | 15032 | W | 11 | OR | patients vs non-patients | 1.13(0.96,1.32) | 0.15 | 60.8 | 0.38 | 13.5 | Null |
|  |  | 737 | 4521 | W | 4 | OR | patients vs non-patients | 1.42(1.02,1.99)^*^ | 0.038 | 51.9 | 0.552 |  | Weak |
| Liang, 2016  (27511796) | hypertension | 22173 | NA | W | 17 | OR | patients vs non-patients | 1.05(0.99,1.11) | 0.115 | 68.2 |  | 12.5 | Null |
| Cheng, 2021  (34166986) | obstructive sleep apnea | 21938 | 1413262 | W | 2 | OR | patients vs non-patients | 1.43(0.54,3.80) | 0.469 | 99.5 | NA | 13 | Null |
| Gómez-Izquierdo, 2020  (32517676) | subclinical hypothyroidism | 480 | 145632 | W and A | 3 | OR | patients vs non-patients | 0.63(0.39,1.03) | 0.064 | 36.8 | 0.649 | 13.5 | Null |
| Wang, 2020  (32282699) | finasteride | 17093 | 140702 | W and A (n=2) | 8 | OR | users v non-users | 0.70(0.51,0.96) | 0.028 | 98.7 | 0.305 | 14 | Suggestive |
|  | finasteride | 3552 | 18685 | W and A | 8 | OR | users v non-users | 2.10(1.85,2.38)^*^ | 1.75E-32 | 50.8 | 0.476 |  | Suggestive |
| Wang, 2021  (34277434) | regular use of aspirin | 81485 | 2093539 | W and A (n=1) | 20 | RR | users v non-users | 0.93(0.88,0.97) | 0.002 | 83 | 0.103 | 13.5 | Suggestive |
| Xu, 2022  (36561541) | statins | 979848 | 2209943 | W and A (n=3) | 32 | RR | users v non-users | 0.94(0.82,1.08) | NA | 98 | NA | 14 | Null |
| Cao, 2018  (29514670) | ACEI | NA | 23307 | W | 3 | RR | users v non-users | 0.92(0.77,1.11) | 0.407 | 0 | 0.513 | 10 | Null |
| Osman, 2017  (28591151) | cardiac glycoside | 11792 | 274318 | W | 4 | RR | users v non-users | 1.08(0.85,1.37) | 0.526 | 81.2 | 0.777 | 12.5 | Null |
| Zhao, 2021  (34414594) | digoxin | 14223 | 81979 | W and A | 6 | HR | users v non-users | 0.89(0.80,0.99) | 0.044 | 47.8 | 0.075 | 12.5 | Suggestive |
| Mahmud, 2010  (20091856) | NSAID | >1000 | NA | W | 6 | OR | users v non-users | 1.00(0.82,1.23) | 0.962 | 87.5 | NA | 12 | Null |
|  | non-aspirin NSAID | >1000 | NA | W | 5 | OR | users v non-users | 0.99(0.89,1.11) | 0.916 | 37.4 | NA |  | Null |
|  |  | >1000 | NA | W | 5 | OR | users v non-users | 1.12(0.68,1.83)^*^ | 0.662 | 91.3 | NA |  | Null |
| Cheng, 2021  (32772072) | vasectomy | 80354 | 4349591 | W | 16 | RR | treated vs non-treated | 1.09(1.04,1.13) | 7.90E-05 | 64.2 | 0.654 | 13.5 | Suggestive |
| Wilson, 2023  (37047163) | bariatric surgery | 4552 | 449789 | W | 4 | RR | treated vs non-treated | 0.78(0.22,2.70) | 0.69 | 96 | NA | 13 | Null |
| Rotshild, 2023  (35645169) | CCB | NR | NR | W | 9 | RR | users vs non-users | 0.99(0.89,1.11) | 0.9 | 56 | NA | 15 | Null |
| Hu, 2023  (36760514) | multiple sclerosis | 4282 | 828942 | W | 8 | RR | patients vs non-patients | 0.78(0.56,1.08) |  | 92.4 | NA | 12 | Null |
| Zhong, 2022  (35341972) | primary Sjögren's syndrome | <1000 | 23295 | W and A (n=2) | 3 | SIR | patients vs non-patients | 1.51(1.02,2.22) | 0.038 | 0 | NA | 15 | Weak |
| Li, 2022  (36192737) | cholelithiasis | NA | 72954 | W and A (n=2) | 3 | RR | patients vs non-patients | 1.25(0.89,1.77) |  | 0 | NA | 13 | Null |
| Cui, 2022  (35074527) | metformin | NR | NR |  | 18 | OR | users vs non-users | 0.93(0.77,1.12) | 0.42 | 97.5 | NR | 15 | Null |
|  | thiazolidinediones | NR | NR |  | 5 | OR | users vs non-users | 0.75(0.53,1.06) | 0.10 | 69.9 | NA |  | Null |
|  | sulfonylureas | >1000 | NR |  | 8 | OR | users vs non-users | 1.02(0.93,1.13) | 0.692 | 45.1 | NA |  | Null |
|  | insulin | NR | NR |  | 13 | OR | users vs non-users | 0.94(0.83,1.05) | 0.26 | 64.6 | NR |  | Null |
| *ENVIRONMENT (6)* | | | | | | | | | | | | |  |
| Dutheil, 2020  (32097115) | asbestos | 15687 | 723566 | W | 30 | SIR | exposed vs unexposed | 1.14(1.07,1.21) | 4.47E-05 | 29.7 | 0.217 | 13 | Highly suggestive |
| Song, 2016  (26871808) | cadmium | 5237 | 114127 | W and A | 4 | RR | exposed vs unexposed | 1.04(0.92,1.18) | 0.49 | 43.4 | 0.453 | 14 | Null |
| Holy, 2022  (34896478) | cobalt | >1000 | 528494 | W | 19 | HR/SIR | exposed vs unexposed | 1.08(1.04,1.14) | 0.001 | 71.2 | 0.975 | 14.5 | Suggestive |
| Krstev, 2019  (31360689) | pesticides | 3067 | NA | W | 13 | SIR/RR | exposed vs unexposed | 1.13(0.97,1.32) | <0.001 | 86 | NA | 8 | Null |
| Li, 2023  (37474858) | green space | NA | NA | W and A (n=2) | 4 | HR | 0.1-unit NDVI (normalized difference vegetation index) increment | 0.95(0.85,1.05) | NA | 87.3 | NA | 13 | Null |
| Yang, 2022  (35550984) | arsenic exposure | NA | 159957 | W and A (n=1) | 6 | RR | exposed vs unexposed | 1.13(1.00,1.28) | 0.057 | 56 | NA | 13.5 | Null |

The statistical test to determine the P value in meta-analyses was using the random-effects inverse-variance model with DerSimonian-Laird method. W, White; A, Asian; RR, risk ratio; OR, odds ratio; HR, hazard ratio; SIR, standard incidence ratio; SRRE, summary relative risk estimate; NR, not reported; NA, not available due to the incomplete reports of original study. Metrics with * denoting advanced, aggressive, high-grade or lethal prostate cancer, metrics with # denoting nonadvanced, nonaggressive or localized prostate cancer. Abbreviations: PA, physical activity; DHA, docosahexaenoic acids; EPA, eicosapentaenoic; HDL, high-density lipoprotein; LDL, low-density lipoprotein; CRP, C-reactive protein; T2D, type 2 diabetes; BPH, benign prostate hyperplasia; HIV, human immunodeficiency virus; AIDS, acquired immune deficiency syndrome; CD, Crohn's disease; UC, ulcerative colitis; AASVs, anti-neutrophil cytoplasm antibody associated vasculitides; ACEI, angiotensin converting enzyme inhibitors; NSAID, nonsteroidal anti-inflammatory drug; CCB, calcium channel blockers.
