# Supplementary material for: Risk factors for prostate cancer: An umbrella review of prospective observational studies and mendelian randomization analyses
Source: PLoS Med. 2024 Mar 15;21(3):e1004362. doi: 10.1371/journal.pmed.1004362 (PMC10980219; doi:10.1371/journal.pmed.1004362)
Supplement: S6 Table — N, number of datasets in the corresponding meta-analysis; OR, odds ratio; CI, confidence interval. Regular use of aspirin: users vs. non-users; Total calcium intake: per 400 mg/d; Coffee: highest vs. lowest; Current smoking: current smoking vs. non-smoker (never smokers plus former smokers); Daidzein: highest vs. lowest; Finasteride: users vs. non-users; Firefighter: ever employment as a career firefighter vs. general population; Height: per 10 cm increase; Soy consumption: highest vs. lowest; Total dairy products: highest vs. lowest; Ulcerative colitis: patients vs. non-patients. (DOCX) [file pmed.1004362.s011.docx]

| S6 Table. Subgroup analyses according to ethnicity (white versus non-white). | | | |
| --- | --- | --- | --- |
| Exposure | **Overall effect size  OR (95%CI)** | **effect size in whites  OR (95%CI)** | **effect size in non-whites  OR (95%CI)** |
| Regular use of aspirin [1] | 0.92 (0.87-0.97) (N=20) | 0.92 (0.86-0.98) | 0.95 (0.88-1.03) (N=1) |
| Total calcium intake [2] | 1.02 (1.01-1.04) (N=9) | 1.02 (1.01-1.04) | 1.16 (0.88-1.53) (N=1) |
| Coffee [3] | 0.91 (0.84-0.98) (N=15) | 0.91 (0.84-0.98) | 0.85 (0.48-1.51) (N=2) |
| Current smoking [4] | 0.74 (0.67-0.81) (N=27) | 0.74 (0.67-0.83) | 0.67 (0.60-0.75) (N=4) |
| Daidzein [5] | 0.75 (0.60-0.93) (N=4) | 0.72 (0.52-1.00) | 0.77 (0.51-1.16) (N=2) |
| Finasteride [6] | 0.70 (0.52-0.95) (N=8) | 0.72 (0.49-1.06) | 0.67 (0.61-0.73) (N=2) |
| Firefighter [7] | 1.21 (1.12-1.32) (N=15) | 1.21 (1.12-1.32) | 1.32 (0.65-2.69) (N=1) |
| Height [8] | 1.09 (1.06-1.12) (N=31) | 1.09 (1.06-1.12) | 1.12 (0.89-1.41) (N=1) |
| Soy consumption [9] | 0.90 (0.82-0.98) (N=7) | 0.90 (0.82-1.00) | 0.86 (0.66-1.11) (N=2) |
| Total dairy products [10] | 1.04 (1.00-1.07) (N=26) | 1.03 (1.00-1.06) | 1.63 (1.14-2.33) (N=1) |
| Ulcerative colitis [11] | 1.23 (1.03-1.46) (N=16) | 1.15 (1.00-1.31) | 1.93 (0.86-4.33) (N=3) |

N, number of datasets included in the corresponding meta-analysis; OR, odds ratio; CI, confidence interval. Regular use of aspirin: users vs non-users; Total calcium intake: per 400mg/d; Coffee: highest vs lowest; Current smoking: current smoking vs non-smoker (never smokers plus former smokers); Daidzein: highest vs lowest; Finasteride: users vs non-users; Firefighter: ever employment as a career firefighter vs general population; Height: per 10 cm increase; Soy consumption: highest vs lowest; Total dairy products: highest vs lowest; Ulcerative colitis: patients vs non-patients.

Reference for the corresponding meta-analysis

1. Wang L, Zhang R, Yu L, Xiao J, Zhou X, Li X, et al. Aspirin Use and Common Cancer Risk: A Meta-Analysis of Cohort Studies and Randomized Controlled Trials. Front Oncol. 2021;11:690219. Epub 2021/07/20. doi: 10.3389/fonc.2021.690219. PubMed PMID: 34277434.

2. Aune D, Navarro Rosenblatt DA, Chan DS, Vieira AR, Vieira R, Greenwood DC, et al. Dairy products, calcium, and prostate cancer risk: a systematic review and meta-analysis of cohort studies. Am J Clin Nutr. 2015;101(1):87-117. Epub 2014/12/21. doi: 10.3945/ajcn.113.067157. PubMed PMID: 25527754.

3. Chen X, Zhao Y, Tao Z, Wang K. Coffee consumption and risk of prostate cancer: a systematic review and meta-analysis. BMJ Open. 2021;11(2):e038902. Epub 2021/01/13. doi: 10.1136/bmjopen-2020-038902. PubMed PMID: 33431520.

4. Yang X, Chen H, Zhang S, Chen X, Sheng Y, Pang J. Association of cigarette smoking habits with the risk of prostate cancer: a systematic review and meta-analysis. BMC Public Health. 2023;23(1):1150. Epub 2023/06/15. doi: 10.1186/s12889-023-16085-w. PubMed PMID: 37316851.

5. Rienks J, Barbaresko J, Nöthlings U. Association of isoflavone biomarkers with risk of chronic disease and mortality: a systematic review and meta-analysis of observational studies. Nutr Rev. 2017;75(8):616-41. Epub 2017/10/04. doi: 10.1093/nutrit/nux021. PubMed PMID: 28969363.

6. Wang L, Lei Y, Gao Y, Cui D, Tang Q, Li R, et al. Association of finasteride with prostate cancer: A systematic review and meta-analysis. Medicine (Baltimore). 2020;99(15):e19486. Epub 2020/04/14. doi: 10.1097/md.0000000000019486. PubMed PMID: 32282699.

7. DeBono NL, Daniels RD, Beane Freeman LE, Graber JM, Hansen J, Teras LR, et al. Firefighting and Cancer: A Meta-analysis of Cohort Studies in the Context of Cancer Hazard Identification. Saf Health Work. 2023;14(2):141-52. Epub 2023/06/30. doi: 10.1016/j.shaw.2023.02.003. PubMed PMID: 37389311.

8. Zuccolo L, Harris R, Gunnell D, Oliver S, Lane JA, Davis M, et al. Height and prostate cancer risk: a large nested case-control study (ProtecT) and meta-analysis. Cancer Epidemiol Biomarkers Prev. 2008;17(9):2325-36. Epub 2008/09/05. doi: 10.1158/1055-9965.Epi-08-0342. PubMed PMID: 18768501.

9. Applegate CC, Rowles JL, Ranard KM, Jeon S, Erdman JW. Soy Consumption and the Risk of Prostate Cancer: An Updated Systematic Review and Meta-Analysis. Nutrients. 2018;10(1). Epub 2018/01/05. doi: 10.3390/nu10010040. PubMed PMID: 29300347.

10. Zhao Z, Wu D, Gao S, Zhou D, Zeng X, Yao Y, et al. The association between dairy products consumption and prostate cancer risk: a systematic review and meta-analysis. Br J Nutr. 2023;129(10):1714-31. Epub 2022/08/10. doi: 10.1017/s0007114522002380. PubMed PMID: 35945656.

11. Zhou BG, Yu Q, Jiang X, Mei YZ, Ding YB, Wang M. Association between inflammatory bowel disease and risk of incident prostate cancer: a systematic review and meta-analysis of cohort studies. Int J Colorectal Dis. 2023;38(1):168. Epub 2023/06/13. doi: 10.1007/s00384-023-04465-y. PubMed PMID: 37310514.
