# Supplementary material for: Risk factors for prostate cancer: An umbrella review of prospective observational studies and mendelian randomization analyses
Source: PLoS Med. 2024 Mar 15;21(3):e1004362. doi: 10.1371/journal.pmed.1004362 (PMC10980219; doi:10.1371/journal.pmed.1004362)
Supplement: S7 Table — The statistical test to determine the P value in MR study was the inverse variance weighted (IVW) regression analysis; § denoting the exposure population source was of Asian ancestry or mixed ancestry; * denoting the outcome of MR studies was aggressive prostate cancer; # denoting the outcome of MR studies was early-onset prostate cancer; ⸸ denoting the summary metric of this MR study was beta estimates. NA, not available; SD, standard deviation; PRACTICAL: The Prostate Cancer Association Group to Investigate Cancer Associated Alterations in the Genome consortium; PA, physical activity; BMI, body mass index; UFA, unfavorable adiposity; FA, favorable adiposity; HbA1c, hemoglobin A1c; GST, glutathione s-transferase; SOD, superoxide dismutase; CAT, catalase; GPX, glutathione peroxidase; IL, interleukin; IL-1b, IL-1 beta; IL-1ra, IL-1 receptor antagonist; IL-2ra, IL-2 receptor alpha subunit; IL-6ra, IL-6 receptor subunit alpha; ALT, alanine aminotransferase; VEGF, vascular endothelial growth factor; IGF, insulin-like growth factor; IGFBP, IGF-binding protein; TOR1AIP1, Torsin-1A-interacting protein 1; MUFAs, monounsaturated fatty acids; AA, Arachidonic acid; ALA, α -linolenic acid; DHA, Docosahexaenoic acid; DPA, Docosapentaenoic acid; EPA, Eicosapentaenoic acid; LA, linoleic acid; OA, Oleic acid; PA, Palmitic acid; POA, Palmitoleic acid; SA, Stearic acid; CRP, C-reactive protein; HDL, high-density lipoprotein; LDL, low-density lipoprotein; Lp(a), lipoprotein A; TG, triglyceride; apo A, apoprotein A; apo B, apoprotein B; VLDL, very low-density lipoprotein; S.HDL.TG, Triglycerides in small HDL; M.VLDL.TG, Triglycerides in medium VLDL; PDGF-bb, platelet-derived growth factor BB; β-NGF, beta nerve growth factor; SCGF-β, stem cell growth factor-beta; HGF, hepatocyte growth factor; CCL2, Chemokine (C-C motif) ligand 2; CCL4, Chemokine (C-C motif) ligand 4; IDO 1, Indoleamine 2,3-dioxygenase 1; MSP, microseminoprotein-beta; LTL, leukocyte telomere length; SHBG, sex-hormone bi [file pmed.1004362.s012.docx]

**S7 Table. Basic characteristics of included Mendelian randomization studies and evidence grading results.**

| Author, year | Exposure | Exposure sample size | Number of IVs | R^2^ | F statistic | Outcome cases/controls | Units of estimates | Summary of metric  by IVW | Main analysis p-value | Quality  (Sensitivity analysis) | Evidence grading |
| --- | --- | --- | --- | --- | --- | --- | --- | --- | --- | --- | --- |
| *LIFESTYLE (N=10)* | | | | | | | | | | | |
| Sun, 2021  (33878190) | morning chronotype | 697828  GWAS/Jones et al., 2019 | 268 | 9.62% | 29.8-429.6 | 79148/61106 PRACTICAL | 1 h earlier | 0.71 (0.54,0.94) | 0.02 | yes | Robust |
| Zhan, 2023  (37237487) | age of sexual initiation | 397338  GWAS/ebi | 167 | 0.70% | 16.7 | 79148/61106 PRACTICAL and GAME-ON/ELLIPSE | older age | 1.18 (1.01,1.38) | 0.035 | yes | Suggestive |
| Huang, 2023  (35975633) | cannabis | 151945  UK Biobank | 44 | NA | NA | 10192/198980  UK Biobank | lifetime cannabis use | 1.06 (0.96,1.17) | 0.22 | yes | Null |
| Larsson, 2022  (35816897) | smoking initiation | 1223091+518633  GWAS and SCANC&UKBB | 297 | <0.01 | 29-211 | 95729/326617  UK Biobank&PRACTICAL&FinnGen | 1 SD smoking initiation | 0.91 (0.86,0.97) | NA | yes | Robust |
| Yuan, 2021  (33619316) | education attainment | 1131881  GWAS/Lee et al., 2018 | 663 | NA | NA | 79194/61112  PRACTICAL | 1 SD (4.2 years) | 1.10 (1.01,1.21) | 0.04 | yes | Probable |
| Kazmi, 2020  (31802111) | accelerometer-measured  PA | 91084  GWAS/Klimentidis et al., 2018 | 2 | 0.10% | 44.68 | 79148/61106  PRACTICAL | 1 SD | 0.49 (0.33,0.72) | 3.00E-04 | no | Insufficient |
|  | sedentary behavior | 91105  GWAS/Doherty et al., 2018 | 2 | 0.08% | 34.91 | 79148/61106  PRACTICAL | 1 SD | 0.95 (0.63,1.42) | 0.79 | no | Null |
| Larsson,2020  (32701947) | alcohol | 941280  GWAS/Liu et al., 2019 | 91 | 0.2-0.3% | NA | 79194/61112  PRACTICAL | 1 SD | 0.96 (0.74,1.24) | 0.753 | yes | Null |
| Wang, 2021  (34371827) | coffee consumption | NA  GWAS/Zhong 2019 | 15 | NA | NA | 79194/61112  PRACTICAL | 1% coffee consumption change | 1.00 (0.99,1.01) | 0.65 | yes | Null |
| Titova, 2020  (32895918) | short sleep duration | 411934  UK Biobank | 27 | NA | NA | 7872/359714  UK Biobank | 1 unit in log odds | 1.08 (0.86,1.37) | 0.51 | no | Null |
| *DIET & NUTRITION (N=2)* | |  |  |  |  |  |  |  |  |  |  |
| Kazmi, 2020  (31802111) | dairy products (milk intake) | 74241  MR/Mørup Bergholdt et al., 2018 | 1 | 0.52% | 388.87 | 15167/58308  PRACTICAL | 1 SD | 0.99 (0.94,1.05)^*^ | 0.81 | no | Null |
| Jin, 2022  (35923199) | dried fruit intake | 421764  UK Biobank | 41 | <0.001 | 17.5-47.9 | 79148/61106  GWAS/Schumacher FR et al..2018 | 1 SD increase of dried fruit intake | 1.14 (0.75,1.74) | 0.5354 | yes | Null |
| *ANTHROPOMETRIC INDICES (N=9)* | | | | | | | | | | | |
| Day, 2017  (28436984) | puberty timing | 329345  ReproGen 23andME, UK Biobank | 375 | NA | NA | 20219/20440  PRACTICAL | 1 year | 0.93 (0.88,0.98) |  | no | Insufficient |
| Kazmi, 2020  (31802111) | adult height | 253288  GWAS/Wood et al., 2014 | 433 | 12.01% | 79.71 | 15167/58308  PRACTICAL | 1 SD | 1.07 (1.01,1.15)^*^ | 0.03 | no | Insufficient |
|  | birth weight | 143677  GWAS/Horikoshi et al., 2016^§^ | 46 | 1.69% | 53.54 | 79148/61106  PRACTICAL | 1 SD | 1.03 (0.89,1.18) | 0.73 | no | Null |
|  | waist circumference | 232101  GWAS/Shungin et al., 2015 | 45 | 0.95% | 49.6 | 79148/61106  PRACTICAL | 1 SD | 0.91 (0.77,1.09) | 0.32 | yes | Null |
|  | waist-hip ratio | 212244  GWAS/Shungin et al., 2015 | 31 | 0.65% | 44.95 | 79148/61106  PRACTICAL | 1 SD | 0.92 (0.78,1.08) | 0.30 | no | Null |
|  | total fat | 13505  GWAS/Kettunen et al., 2016 | 12 | 4.62% | 54.51 | 79148/61106  PRACTICAL | 1 SD | 0.99 (0.91,1.08) | 0.84 | no | Null |
| Perez-Cornago，2023  (37305903) | BMI | 694649  UK Biobank and GIANT | 506 | 5.80% | 71.4-149.4 | 85554/91972  PRACTICAL | 1 SD | 0.92 (0.85,1.00) | 0.047 | yes | Robust |
|  | UFA | 429203  UK Biobank | 27 | 1.44% | 71.4-149.4 | 85554/91972  PRACTICAL | 1 SD | 0.82 (0.67,1.01) | 0.056 | yes | Probable  (P-weighted median:0.014) |
|  | FA | 429203  UK Biobank | 34 | 0.64% | 71.4-149.4 | 85554/91972  PRACTICAL | 1 SD | 0.97 (0.77,1.20) | 0.76 | yes | Null |
| *BIOMARKERS (N=98)* | | | | | | | | | | | |
| Luo, 2020  (32748028) | HbA1c (%) reduction | 123665  MAGIC | 13 | NA | NA | 8970/398757  UK Biobank | 1% decrease | 0.81 (0.52,1.28) | 0.37 | yes | Null |
| Zhu, 2023  (37441531) | vitamin E | 64979  UK Biobank | 12 | 0.0043 | 23.1775 | 6311/74685  FinnGen Biobank | 1 SD | 1.02 (0.70,1.50) | 0.6868 | no | Null |
|  | CAT | 3301  INTERVAL study (PMID:29875488) | 16 | 0.1317 | 31.1421 | 6311/74685  FinnGen Biobank | 1 SD | 0.96 (0.89,1.04) | 0.3224 | yes | Null |
|  | GST | 3301  INTERVAL study (PMID:29875488) | 18 | 0.2229 | 52.2968 | 6311/74685  FinnGen Biobank | 1 SD | 1.01 (0.94,1.07) | 0.863 | yes | Null |
|  | SOD | 3301  INTERVAL study (PMID:29875488) | 12 | 0.0993 | 27.8713 | 6311/74685  FinnGen Biobank | 1 SD | 1.04 (0.94,1.16) | 0.3996 | yes | Null |
|  | GPX | 3301  INTERVAL study (PMID:29875488) | 25 | 0.295 | 54.8247 | 6311/74685  FinnGen Biobank | 1 SD | 1.01(0.95,1.07) | 0.8096 | yes | Null |
|  | albumin | 115060  KORA study (PMID:24816252) | 128 | 0.0504 | 44.5391 | 6311/74685  FinnGen Biobank | 1 SD | 0.79 (0.68,0.91) | 0.001 | yes | Robust |
|  | total bilirubin | 342829  UK Biobank | 503 | 0.502 | 657.2863 | 6311/74685  FinnGen Biobank | 1 SD | 1.01(0.99,1.01) | 0.1507 | yes | Null |
|  | retinol | 62991  UK Biobank | 8 | 0.0029 | 23.2573 | 6311/74685  FinnGen Biobank | 1 SD | 1.41 (0.83,2.37) | 0.6928 | yes | Null |
|  | vitamin B6 | 62991  UK Biobank | 16 | 0.0061 | 23.3452 | 6311/74685  FinnGen Biobank | 1 SD | 0.83 (0.56,1.24) | 0.9425 | yes | Null |
|  | vitamin C | 62991  UK Biobank | 11 | 0.0039 | 23.1618 | 6311/74685  FinnGen Biobank | 1 SD | 1.34 (0.89,2.01) | 0.9585 | yes | Null |
| Lu, 2023  (36923697) | serum copper concentration | 2603  GWAS/David et al., 2013 | 2 | NA | NA | 79148/61106  PRACTICAL | 1 SD | 1.01 (0.95,1.06) | 0.867 | no | Null |
|  | zinc | 2603  GWAS/David et al., 2013 | 2 | NA | NA | 79148/61106  PRACTICAL | 1 SD | 1.06 (1.00,1.12) | 0.0403 | no | Insufficient |
|  | blood selenium | 2603  GWAS/David et al., 2013 | 12 | NA | NA | 79148/61106  PRACTICAL | 1 SD | 0.99 (0.95,1.03) | 0.458 | yes | Null |
| Wei, 2023  (36880394) | Class. Alphaproteobacteria | 18340  MiBioGen | 7 | <0.1 | >10 | 79148/61106  PRACTICAL | 1 SD | 0.84 (0.75,0.93) | 1.11E-03 | yes | Probable |
|  | Order. Rhodospirillales | 18340  MiBioGen | 14 | <0.1 | >10 | 79148/61106  PRACTICAL | 1 SD | 0.91 (0.85,0.97) | 6.24 E-03 | Yes | Probable |
|  | Genus. Adlercreutzia | 18340  MiBioGen | 8 | <0.1 | >10 | 79148/61106  PRACTICAL | 1 SD | 0.89 (0.82,0.97) | 5.18E-03 | Yes | Probable |
|  | Genus. Coprobacter | 18340  MiBioGen | 11 | <0.1 | >10 | 79148/61106  PRACTICAL | 1 SD | 0.92 (0.87,0.98) | 8.28E-03 | Yes | Suggestive |
| Li, 2023  (36733309) | IL-1b | 13577  GWAS/Sliz et al. | 1 | <0.001 | 50 | 97253/355362  PRACTICAL&UKBB&FinnGen | 1 SD | 1.14 (0.83,1.51) | 0.48 | no | Null |
|  | IL-1ra | >30000  GWAS/Folkersen et al. | 4 | <0.001 | 221 | 97253/355362  PRACTICAL&UKBB&FinnGen | 1 SD | 0.92 (0.89,0.96) | 1.58E-05 | yes | Probable |
|  | IL-2ra | 8293  GWAS/Ahola-Olli et al. | 1 | <0.001 | 164 | 97253/355362  PRACTICAL&UKBB&FinnGen | 1 SD | 1.00 (0.96,1.03) | 0.781 | no | Null |
|  | IL-6 | >30000  GWAS/Folkersen et al. | 2 | <0.001 | 118 | 97253/355362  PRACTICAL&UKBB&FinnGen | 1 SD | 1.12 (1.07,1.17) | 6.61E-07 | no | Insufficient |
|  | IL-6ra | >30000  GWAS/Folkersen et al. | 4 | <0.001 | 473 | 97253/355362  PRACTICAL&UKBB&FinnGen | 1 SD | 1.02 (1.00,1.04) | 0.05 | yes | Null |
|  | IL-8 | >30000  GWAS/Folkersen et al. | 2 | <0.001 | 47 | 97253/355362  PRACTICAL&UKBB&FinnGen | 1 SD | 0.94 (0.85,1.05) | 0.519 | no | Null |
|  | IL-16 | >30000  GWAS/Folkersen et al. | 8 | <0.001 | 529 | 97253/355362  PRACTICAL&UKBB&FinnGen | 1 SD | 0.99 (0.96,1.02) | 0.465 | yes | Null |
|  | IL-17 | 8293  GWAS/Ahola-Olli et al. | 1 | <0.001 | 40 | 97253/355362  PRACTICAL&UKBB&FinnGen | 1 SD | 1.02 (0.90,1.17) | 0.721 | no | Null |
|  | IL-18 | >30000  GWAS/Folkersen et al. | 8 | <0.001 | 138 | 97253/355362  PRACTICAL&UKBB&FinnGen | 1 SD | 0.99 (0.95,1.04) | 0.833 | yes | Null |
|  | IL-27 | >30000  GWAS/Folkersen et al. | 11 | <0.001 | 485 | 97253/355362  PRACTICAL&UKBB&FinnGen | 1 SD | 0.99 (0.96,1.01) | 0.434 | yes | Null |
| Deng, 2023  (36542132) | serum uric acid | 121745  J-MICC&KING&BBJ | 34 | 1.66% | 60 | 5408/103939  Biobank Japan | 1SD(1.4mg/dL) | 1.12 (1.00,1.26) | 0.043 | yes | Robust |
| Yang, 2022  (36330075) | alanine | 86507  EPIC-Norfolk and INTERV | 16 | 0.117 | 41.65 | 79148/61106  PRACTICAL | 1 SD | 1.16 (1.01,1.33) | 0.037 | yes | Robust |
|  | ALT | 315572  UK Biobank | 237 | 0.058 | 87.45 | 79148/61106  PRACTICAL | log10 | 0.43 (0.27,0.68) | 3.28E-04 | yes | Robust |
| Wu, 2022  (36159967) | VEGF | 7118  GWAS/Ahola-Olli et al., 2017 | 11 | 0.15 | 104.5 | 6311/88902  FinnGen | 1 SD | 1.02 (0.95,1.09) | 0.66 | no | Null |
| Watts, 2022  (35726641) | IGF-I | 158444  GWAS/Neale lab UKB, 2019 | 154 | 8.70% | NA | 15167/58308  PRACTICAL | 1 SD | 1.10 (1.01,1.20)^*^ | 0.04 | yes | Probable |
|  | IGFBP-3 | 9331  From 20 interval studies | NA | NA | NA | 15167/58308  PRACTICAL | 1 SD | 1.08 (1.04,1.11) | <0.0001 | no | Insufficient |
| Zheng, 2020  (32895551) | resistin | 10162  GWAS/Yao et al., 2018, Sun et al., 2018 | 4 | NA | NA | 2273/334926  Neale's Lab | 1 SD | 0.91 (0.73,1.13) | 0.38 | no | Null |
|  | TOR1AIP1 | 3301  GWAS/Sun et al., 2018 | 3 | NA | NA | 2273/334926  Neale's Lab | 1 SD | 1.14 (1.02,1.27) | 0.02 | no | Insufficient |
| Kazmi, 2020  (31802111) | β-carotene | 3932  GWAS/Ferrucci et al., 2009 | 1 | 2.63% | >99 | 79148/61106  PRACTICAL | 1 SD | 0.97 (0.90,1.04) | 0.37 | no | Null |
|  | α-carotene | 433  GWAS/D'adamo et al., 2016 | 3 | 22.08% | >99 | 79148/61106  PRACTICAL | 1 SD | 1.00 (0.98,1.03) | 0.72 | no | Null |
|  | serum iron | 72958  GWAS/Benyamin et al., 2015 | 5 | 2.20% | 328.85 | 79148/61106  PRACTICAL | 1 SD | 0.92 (0.86,0.98) | 0.01 | no | Insufficient |
|  | lycopene | 441  GWAS/D'adamo et al., 2016^§^ | 1 | 8.34% | >99 | 79148/61106  PRACTICAL | 1 SD | 0.97 (0.88,1.07) | 0.55 | no | Null |
|  | serum calcium | 61079  GWAS/O’Seaghdha et al., 2013^§^ | 5 | 0.65% | 79.85 | 79148/61106  PRACTICAL | 1 SD | 0.66 (0.38,1.14) | 0.14 | no | Null |
|  | MUFAs | 13535  GWAS/Kettunen et al., 2016 | 5 | 2.01% | 55.62 | 79148/61106  PRACTICAL | 1 SD | 1.11 (1.02,1.20) | 0.02 | yes | Suggestive |
| Ye, 2020  (32918767) | 25-hydroxyvitamin D | 417580  GWAS/Revez et al., 2020 | 145 | 0.075 | NA | GWAS/Schumacher et al., 2018 | 1 SD | 0.99 (0.98,1.01) | 0.42 | yes | Null |
| Kim, 2023  (37605270) | vitamin B12 | 37283  GWAS/Grarup, et al., 2013 | 6 | NA | 68.31-432.75 | 18008/282144  PRACTICAL | 1 SD | 1.09 (1.02,1.16) | 0.0103 | yes | Probable |
| Ying, 2022  (36561528) | transferrin saturation | 48972  GISC | 3 | 0.0731 | 46.9-2126.74 | 79148/61106  PRACTICAL | 1 SD | 0.94 (0.88,0.99) | 0.029 | yes | Probable |
| Lv, 2022  (34617559) | circulating phosphorous | GWAS/UKB, Sinnott-Armstrong et al., 2021 | 125 | NA | NA | 79148/61106  PRACTICAL | 1 SD | 1.19 (1.09,1.31) | 1.82E-04 | yes | Robust |
| Yang, 2021  (34276774) | ALA | 8866  GWAS/Lemaitre et al., 2011 | 1 | NA | NA | 79148/61106  PRACTICAL | per allele effect | 0.87 (0.38,1.99) | 0.73 | no | Null |
|  | EPA | 8866  GWAS/Lemaitre et al., 2011 | 2 | NA | NA | 79148/61106  PRACTICAL | per allele effect | 1.12 (0.93,1.36) | 0.22 | no | Null |
|  | DPA | 8866  GWAS/Lemaitre et al., 2011 | 2 | NA | NA | 79148/61106  PRACTICAL | per allele effect | 1.10 (0.91,1.32) | 0.36 | no | Null |
|  | DHA | 8866  GWAS/Lemaitre et al., 2011 | 1 | NA | NA | 79148/61106  PRACTICAL | per allele effect | 0.89 (0.77,1.03) | 0.12 | no | Null |
|  | LA | 8631  GWAS/Guan et al., 2014 | 3 | NA | NA | 79148/61106  PRACTICAL | per allele effect | 1.00 (0.98,1.02) | 0.89 | yes | Null |
|  | AA | 8631  GWAS/Guan et al., 2014 | 2 | NA | NA | 79148/61106  PRACTICAL | per allele effect | 1.00 (0.98,1.02) | 0.78 | no | Null |
|  | POA | 8961  GWAS/Wu et al., 2013 | 4 | NA | NA | 79148/61106  PRACTICAL | per allele effect | 0.88 (0.57,1.35) | 0.56 | yes | Null |
|  | OA | 8961  GWAS/Wu et al., 2013 | 1 | NA | NA | 79148/61106  PRACTICAL | per allele effect | 1.00 (0.93,1.07) | 0.93 | no | Null |
|  | PA | 8961  GWAS/Wu et al., 2013 | 1 | NA | NA | 79148/61106  PRACTICAL | per allele effect | 1.01 (0.93,1.11) | 0.76 | no | Null |
|  | SA | 8961  GWAS/Wu et al., 2013 | 3 | NA | NA | 79148/61106  PRACTICAL | per allele effect | 1.01 (0.95,1.07) | 0.80 | yes | Null |
| Lin, 2022  (35296245) | glutamate | 1960  GWAS/Long et al., 2017 | 5 | NA | NA | 79148/61106  PRACTICAL | 1 SD | 1.03 (0.95,1.12) | 0.49 | yes | Null |
|  | aspartate | 1960  GWAS/Long et al., 2017 | 13 | NA | NA | 79148/61106  PRACTICAL | 1 SD | 1.04 (1.00,1.08) | 0.03 | yes | Suggestive |
| He, 2021  (34504857) | homocystein | 44147  GWAS/van Meurs et al, 2013 | 15 | NA | NA | 79148/61106  PRACTICAL | 1 SD | 1.01 (0.93,1.11) | 0.77 | yes | Null |
| Sun, 2021  (33805346) | serum urea | 13312  GWAS/Thio et al., 2019 | 6 | NA | NA | 79148/61106  PRACTICAL | 1 SD | 1.02 (0.94,1.11) | 0.70 | yes | Null |
| Li, 2022  (35494045) | tryptophan | 7824  GWAS/Shin et al., 2014 | 18 | 0.038 | NA | 79148/61106  PRACTICAL | 1 SD | 0.40 (0.13,1.22) ^⸸^ | 0.11 | yes | Null |
|  | kynurenine | 7824  GWAS/Shin et al., 2014 | 4 | 0.0119 | NA | 79148/61106  PRACTICAL | 1 SD | 0.79 (0.46,1.34) ^⸸^ | 0.38 | yes | Null |
| Larsson, 2021  (33199044) | plasma phospholipid AA concentrations | 8866  GWAS/Guan et al., 2014 | 2 | NA | NA | 95710/381889  PRACTICAL, UK Biobank, FinnGen, BBJ | 1 SD increase | 1.02 (1.00,1.04) | 0.11 | no | Null |
| He, 2020  (33178578) | CRP | 204402  GWAS/Ligthart et al., 2018 | 58 | 0.07 | NA | 79148/61106  PRACTICAL | 1 unit increase in natural log | 1.06 (0.96,1.16) | 0.24 | yes | Null |
| Ioannidou, 2022  (35085228) | HDL | 167020  GWAS/Neale lab UKB, 2020 | 124 | 0.36 | NA | 79148/61106  PRACTICAL | 1 SD increase | 0.99 (0.94,1.05) | 0.41 | yes | Null |
|  | LDL | 167020  GWAS/Neale lab UKB, 2020 | 72 | 0.29 | NA | 6988/44256  PRACTICAL | 1 SD increase | 1.23 (1.04,1.45)^#^ | 0.02 | yes | Probable |
|  | TG | 167020  GWAS/Neale lab UKB, 2020 | 96 | 0.29 | NA | 6988/44256  PRACTICAL | 1 SD increase | 1.22 (1.04,1.44)^#^ | 0.02 | yes | Probable |
|  | Lp(a) | 167020  GWAS/Neale lab UKB, 2020 | 10 | 0.24 | NA | 79148/61106  PRACTICAL | 1 SD increase | 1.07 (0.91,1.25) | 0.43 | yes | Null |
|  | apo A | 167020  GWAS/Neale lab UKB, 2020 | 115 | 0.31 | NA | 79148/61106  PRACTICAL | 1 SD increase | 1.03 (0.97,1.08) | 0.37 | yes | Null |
|  | apo B | 167020  GWAS/Neale lab UKB, 2020 | 80 | 0.32 | NA | 79148/61106  PRACTICAL | 1 SD increase | 1.03 (0.96,1.09) | 0.41 | yes | Null |
| Adams, 2019  (30352818) | S.HDL.TG | 24925  GWAS/Kettunen et al., 2016 | NA | NA | NA | 44825/27904  PRACTICAL | 1 SD | 0.91 (0.83,1.01) | 0.07 | no | Null |
|  | M.VLDL.TG | 24925  GWAS/Kettunen et al., 2016 | NA | NA | NA | 44825/27904  PRACTICAL | 1 SD | 0.89 (0.80,0.99) | 0.04 | no | Insufficient |
|  | lactate | 24925  GWAS/Kettunen et al., 2016 | NA | NA | NA | 44825/27904  PRACTICAL | 1 SD | 1.38 (1.02,1.86) | 0.03 | no | Insufficient |
|  | A1-acid glycoprotein | 24925  GWAS/Kettunen et al., 2016 | NA | NA | NA | 44825/27904  PRACTICAL | 1 SD | 1.17 (1.04,1.31) | 6.42E-03 | no | Insufficient |
|  | creatinine | 24925  GWAS/Kettunen et al., 2016 | NA | NA | NA | 44825/27904  PRACTICAL | 1 SD | 1.41 (1.20,1.66) | 3.05E-05 | no | Insufficient |
| Sun, 2020  (33460126) | PDGF-bb | 8239  GWAS/Ahola-Olli et al., 2017 | 21 | 14.00% | 41.27 | 79148/61106  PRACTICAL | 1 SD | 0.97 (0.95,1.00) | 0.08 | yes | Probable |
|  | β-NGF | 8239  GWAS/Ahola-Olli et al., 2017 | 2 | 2.10% | 35.27 | 79148/61106  PRACTICAL | 1 SD | 0.92 (0.85,0.99) | 0.03 | no | Insufficient |
|  | SCGF-β | 8239  GWAS/Ahola-Olli et al., 2017 | 21 | 24.60% | 46.43 | 79148/61106  PRACTICAL | 1 SD | 0.95 (0.94,0.97) | 6.27E-08 | yes | Probable |
|  | HGF | 8239  GWAS/Ahola-Olli et al., 2017 | 6 | 2.30% | 36.37 | 79148/61106  PRACTICAL | 1 SD | 1.07 (1.01,1.13) | 0.03 | yes | Probable |
|  | C-X-C motif chemokine ligand 9 | 8239  GWAS/Ahola-Olli et al., 2017 | 4 | 3.60% | 39.37 | 79148/61106  PRACTICAL | 1 SD | 1.08 (1.02,1.14) | 0.01 | yes | Probable |
|  | CCL4 | 8239  GWAS/Ahola-Olli et al., 2017 | 286 | 100.00% | 40.31 | 79148/61106  PRACTICAL | 1 SD | 1.04 (1.02,1.06) | 8.01E-05 | yes | Probable |
|  | CCL2 | 8239  GWAS/Ahola-Olli et al., 2017 | 35 | 18.20% | 43.64 | 79148/61106  PRACTICAL | 1 SD | 1.06 (1.04,1.09) | 6.95E-06 | yes | Probable |
| Li, 2019  (31186442) | IDO 1 | 3301  GWAS/Sun et al., 2018 | 3 | NA | 155.3 | 79148/61106  PRACTICAL | 1 SD | 0.96 (0.93,0.99) | 0.01 | yes | Probable |
| Byrne, 2019  (31089709) | plasma MSP | 2254  EPIC | 1 | 42% | NA | NA  PRACTICAL | 1 ng/mL | 0.96 (0.95,0.97) | NA | no | Insufficient |
| Beynon,2019  (30325021) | Pyruvate | NA  GWAS/Kettunen et al., 2016 | 2 | 0.22-0.30% | 59.4 | 44825/27904  PRACTICAL | 1 SD | 1.29 (1.03,1.62) | 0.03 | no | Insufficient |
| Wan, 2023  (37352282) | LTL | 472174  GWAS/IEU | 124 | <0.001 | 27.34-189.45 | 79148/61106  EMBL-EBI | Long LTL | 1.37 (1.25,1.50) | 2.84E-11 | yes | Robust |
| He, 2022  (34844025) | adiponectin | 67739  GWAS/ Spracklen et al., 2019 | 15 | NA | NA | 79148/61106  PRACTICAL | 1 SD | 1.00 (0.99,1.01) ^⸸^ | 0.74 | yes | Null |
| Ruth, 2020  (32042192) | SHBG | 180726  UK Biobank | 347 | NA | NA | 67158/48350  PRACTICAL | 1 SD | 0.91 (0.80,1.04) | 0.16 | yes | Null |
|  | estradiol men | 206927  UK Biobank | 20 | NA | NA | 67158/48350  PRACTICAL | 1 SD | 1.12 (0.54,2.33) | 0.77 | yes | Null |
| Chang, 2022  (36482455) | bioavailable testosterone | 184205  GWAS/Hayes BL et al..2018 | 52 | <0.01 | NA | 79148/61106  PRACTICAL | 1 SD | 1.17 (1.09,1.26) | 2.51E-05 | yes | Probable |
| Bryony, 2022  (35061662) | total testosterone | 200159  UK Biobank | 119 | 0.048 | 36.8-143.4 | 79148/61106  ELLIPSE&PRACTICAL | 1 SD | 0.97 (0.86,1.07) | 0.482 | yes | Null |
| Watts, 2022  (35579976) | free testosterone | 194453  GWAS/Ruth et al., 2020 | 67 | NA | NA | 79148/61106  PRACTICAL | 1 SD | 1.20 (1.08,1.34) | 6.00E-04 | yes | Probable |
| Bryony, 2022  (35061662) | oestradiol | 17134  UK Biobank | 2 | 0.004 | 36.8-143.4 | 79148/61106  ELLIPSE&PRACTICAL | 1 SD | 1.05 (0.83,1.33) | 0.0673 | no | Null |
| Yuan, 2020  (32349989) | fasting insulin | 108557  MAGIC | 35 | 4.80% | NA | 7872/292606  UK Biobank | 1 SD | 0.80 (0.57,1.11) | 0.19 | no | Null |
|  | fasting glucose | 133010  MAGIC | 35 | 4.80% | NA | 7872/292606  UK Biobank | 1 SD | 0.97 (0.81,1.17) | 0.77 | no | Null |
| Yuan, 2020  (32215913) | TSH | 54288  ThyroidOmics Consortium | 58 | NA | NA | 7872/292606  UK Biobank | 1 SD | 0.91 (0.84,0.99) | 0.03 | no | Insufficient |
|  | free thyroxine | 49269  ThyroidOmics Consortium | 31 | NA | NA | 7872/292606  UK Biobank | 1 SD | 1.06 (0.93,1.20) | 0.40 | no | Null |
| *CLINICAL VARIABLES, DISEASES, AND TREATMENTS (N=26)* | | | | | | | | | | | |
| Fang, 2023  (36595504) | PCSK9 inhibition | 1320016  GLGC | 28 | NA | 511.1 | 79148/61106  PRACTICAL | 1 SD | 0.85 (0.76,0.96) | 0.009 | yes | Probable |
| Nabila, 2023  (37178364) | CCB | 142995  UK Biobank | 16 | <0.001 | 64-534 | 79148/61106  PRACTICAL | 10 mm Hg- SBP lower | 1.22 (1.06,1.42) | 0.01 | yes | Probable |
| James, 2023  (37171501) | perturbation of PPARG | 407766  UK Biobank | 6 | 0.00070-0.00119 | 285.82-487.14 | 79148/61106  PRACTICAL | 1 unit decrease in HbA1c  (0.09%) | 1.75 (1.07,2.85) | 0.02 | No | Insufficient |
|  | perturbation of ABCC8 | 407766  UK Biobank | 4 | 0.00014-0.00027 | 59.11-111.98 | 79148/61106  PRACTICAL | mmol/mol (0.09%) | 0.94 (0.37,2.43) | 0.91 | No | Null |
|  | perturbation of GLP1R | 407766  UK Biobank | 7 | 0.00014-0.00022 | 56.32-88.82 | 79148/61106  PRACTICAL | mmol/mol (0.09%) | 0.87 (0.35,2.14) | 0.76 | No | Null |
| Yarmolinsky, 2022  (35113855) | ACE inhibition | ≤ 857,386  GWAS/UKB and ICBP,GERA | 14 | 0.34-0.39% | 2,156.5-2,594.9 | 79148/61106  PRACTICAL | a 1-mm Hg decrease in SBP | 1.06 (0.99,1.13) | 0.08 | yes | Null |
|  | ADRB1 inhibition | ≤ 857,386  GWAS/UKB and ICBP,GERA | 8 | 0.00031-0.00067% | 269.1-572.2 | 79148/61106  PRACTICAL | a 1-mm Hg decrease in SBP | 1.00 (0.96,1.03) | 0.73 | yes | Null |
|  | NCC inhibition | ≤ 857,386  GWAS/UKB and ICBP,GERA | 1 | 0.00% | 1659.9 | 79148/61106  PRACTICAL | a 1-mm Hg decrease in SBP | 1.08 (0.96,1.19) | 0.18 | yes | Null |
| Sun, 2022  (35151363) | inhibition of HMG-CoA reductase | 188577  GWAS/Willer et al., 2013 | 5 | NA | 128.14 | 79148/61106  PRACTICAL | 1 SD | 0.85 (0.73,1.00) | 0.05 | yes | Null |
|  | inhibition of NPC1L1 | 188577  GWAS/Willer et al., 2013 | 3 | NA | 71.63 | 79148/61106  PRACTICAL | 1 SD | 1.23 (0.92,1.63) | 0.16 | yes | Null |
| Chan, 2021  (34915881) | SBP | 757601  GWAS/Evangelou et al., 2018 | 268 | NA | NA | 10792/190694  UK Biobank | 1 SD | 1.05 (0.90,1.23) | 0.54 | yes | Null |
|  | DBP | 757601  GWAS/Evangelou et al., 2018 | 267 | NA | NA | 10792/190694  UK Biobank | 1 SD | 1.04 (0.89,1.21) | 0.62 | yes | Null |
| Chen, 2020  (33027558) | MDD | 480359  GWAS/Wray et al., 2018 | 43 | 0.23% | 156 | 79148/61106  PRACTICAL | increased susceptibility | 1.14 (1.00,1.30) | 0.05 | yes | Null |
| Xu, 2023  (37274339) | hyperthyroidism | 462933  MRC- ieu | 13 | NA | 29.87-606.49 | 6321/354873  GWAS/Neale lab | increased susceptibility | 0.86 (0.79,0.94) | 0.0004 | yes | Robust |
| Ou, 2023  (37213031) | SLE | 14267  GWAS | 45 | <0.01 | 12592.46 | 79148/61106  PRACTICAL | increased susceptibility | 0.99 (0.97,0.99) | 0.003 | yes | Probable |
| Cheng, 2023  (37409117) | IBD | 59957  GWAS/M. de Lange et al., 2017 | 103 | 0.001 | 70.93 | 79148/61106  GWAS/Schumacher FR et al., 2018 | increased susceptibility | 0.97 (0.94,1.02) | 0.326 | Yes | Null |
|  | CD | 40266  GWAS/M. de Lange et al., 2017 | 82 | 0.001 | 79.54 | 79148/61106  GWAS/Schumacher FR et al.2018 | increased susceptibility | 0.97 (0.93,1.01) | 0.235 | Yes | Null |
|  | UC | 45975  GWAS/M. de Lange et al.,2017 | 53 | 0.001 | 69.85 | 79148/61106  GWAS/Schumacher FR et al.2018 | increased susceptibility | 0.98 (0.96,1.02) | 0.416 | yes | Null |
| Li, 2022  (36684576) | atrial fibrillation | 1030836  GWAS/ebi | 108 | <0.001 | NA | 79148/61106  PRACTICAL | increased susceptibility | 0.96 (0.92,0.99) | 0.01 | yes | Probable |
|  | heart failure | 977323  GWAS/ebi | 9 | <0.001 | NA | 79148/61106  PRACTICAL | increased susceptibility | 1.11 (0.92,1.35) | 0.25 | yes | Null |
| Jiang, 2020  (32006205) | allergic disease | 477396  GWAS/Ferreira et al, 2017, Zhu et al, 2018 | 132 | 1.18% | NA | 79148/61106  PRACTICAL | increased susceptibility | 1.00 (0.94,1.05) | 0.93 | yes | Null |
|  | asthma | 110361  GWAS/Zhu et al., 2018 | 32 | NA | NA | 79148/61106  PRACTICAL | increased susceptibility | 1.03 (0.96,1.09) | 0.43 | yes | Null |
| Wen, 2020  (32462299) | vitiligo | NA  GWAS/Jin et al., 2010, Jin et al., 2016 | 37 | 17.4% | 29546.03 | 79148/61106  PRACTICAL | increased susceptibility | 1.01 (0.99,1.03) | 0.31 | yes | Null |
| Yuan, 2020  (32215913) | hypothyroidism | 53423  ThyroidOmics Consortium | 7 | NA | NA | 7872/292606  UK Biobank | increased susceptibility | 0.96 (0.89,1.04) | 0.31 | no | Null |
| Au, 2019  (31908803) | T2D | 898130  DIAGRAM | 112 | NA | 72.4 | 79148/61106  PRACTICAL | increased susceptibility | 1.02 (0.97,1.07) | NA | yes | Null |
| Ge, 2022  (35303584) | schizophrenia | NA  Schizophrenia Psychiatric GWAS Consortium, 2011 | 75 | NA | 221.77 | 79148/61106  PRACTICAL | increased susceptibility | 1.03 (0.99,1.07) | 0.07 | yes | Null |

The statistical test to determine the P value in MR study was the Inverse Variance Weighted (IVW) regression analysis.

^§^ denoting the exposure population source was of Asian ancestry or mixed ancestry.

^*^ denoting the outcome of MR studies was aggressive prostate cancer;

^#^ denoting the outcome of MR studies was early-onset prostate cancer;

⸸ denoting the summary metric of this MR study was beta estimates.

NA, not available; SD, standard deviation; PRACTICAL: The Prostate Cancer Association Group to Investigate Cancer Associated Alterations in the Genome consortium.

Abbreviations: PA, physical activity; BMI, body mass index; UFA, unfavourable adiposity; FA, favourable adiposity; HbA1c, hemoglobin A1c; GST, glutathione s-transferase; SOD, superoxide dismutase; CAT, catalase; GPX, glutathione peroxidase; IL, interleukin; IL-1b, IL-1 beta; IL-1ra, IL-1 receptor antagonist; IL-2ra, IL-2 receptor alpha subunit; IL-6ra, IL-6 receptor subunit alpha; ALT, alanine aminotransferase; VEGF, vascular endothelial growth factor; IGF, insulin-like growth factor; IGFBP, IGF-binding protein; TOR1AIP1, Torsin-1A-interacting protein 1; MUFAs, monounsaturated fatty acids; AA, Arachidonic acid; ALA, α -linolenic acid; DHA, Docosahexaenoic acid; DPA, Docosapentaenoic acid; EPA, Eicosapentaenoic acid; LA, linoleic acid; OA, Oleic acid; PA, Palmitic acid; POA, Palmitoleic acid; SA, Stearic acid; CRP, C-reactive protein; HDL, high-density lipoprotein; LDL, low-density lipoprotein; Lp(a), lipoprotein A; TG, triglyceride; apo A, apoprotein A; apo B, apoprotein B; VLDL, very low-density lipoprotein; S.HDL.TG, Triglycerides in small HDL; M.VLDL.TG, Triglycerides in medium VLDL; PDGF-bb, platelet-derived growth factor BB; β-NGF, beta nerve growth factor; SCGF-β, stem cell growth factor-beta; HGF, hepatocyte growth factor; CCL2, Chemokine (C-C motif) ligand 2; CCL4, Chemokine (C-C motif) ligand 4; IDO 1, Indoleamine 2,3-dioxygenase 1; MSP, microseminoprotein-beta; LTL, leukocyte telomere length; SHBG, sex-hormone binding globulin; TSH, thyroid-stimulating hormone; CCB, calcium channel blockers; PCSK9, proprotein convertase subtilisin/kexin type 9; PPARG, peroxisome proliferator activated receptor γ; ABCC8, ATP binding cassette subfamily C member 8; GLP1R, glucagon-like peptide 1 receptor; ACE, angiotensin-converting enzyme; ADRB1, β-1 adrenergic receptor; NCC, sodium-chloride symporter; SBP, systolic blood pressure; DBP, diastolic blood pressure; MDD, major depressive disorder; SLE, systemic lupus erythematosus; IBD, inflammatory bowel disease; CD, Crohn's disease; UC, ulcerative colitis; T2D, type 2 diabetes; HMG-CoA, 3-hydroxy-3-methylglutaryl coenzyme A; NPC1L1, Niemann-Pick C1-Like 1.
