# Supplementary material for: Risk factors for prostate cancer: An umbrella review of prospective observational studies and mendelian randomization analyses
Source: PLoS Med. 2024 Mar 15;21(3):e1004362. doi: 10.1371/journal.pmed.1004362 (PMC10980219; doi:10.1371/journal.pmed.1004362)
Supplement: S8 Table — Metrics with * denoting the outcome was advanced, aggressive, high-grade, or lethal prostate cancer. Other null associations of biomarkers in MR studies were recorded in a previous review by Markozannes and colleagues (reference [19]). PA, physical activity; DHA, docosahexaenoic acids; EPA, eicosapentaenoic; HDL, high-density lipoprotein; LDL, low-density lipoprotein; CRP, C-reactive protein; T2D, type 2 diabetes; BPH, benign prostate hyperplasia; HIV, human immunodeficiency virus; AIDS, acquired immune deficiency syndrome; CD, Crohn’s disease; UC, ulcerative colitis; AASVs, anti-neutrophil cytoplasm antibody associated vasculitides; ACEI, angiotensin converting enzyme inhibitors; NSAID, nonsteroidal anti-inflammatory drug; CCB, calcium channel blockers; TG, triglyceride; MUFAs, monounsaturated fatty acids; MDD, major depressive disorder; LTL, leukocyte telomere length; IGF, insulin-like growth factor; IGFBP, IGF-binding protein; TOR1AIP1, Torsin-1A-interacting protein 1; IL-6ra, IL-6 receptor subunit alpha; IDO 1, Indoleamine 2,3-dioxygenase 1; SCGF-β, stem cell growth factor-beta; β-NGF, beta nerve growth factor; MSP, microseminoprotein-beta; ALT, alanine aminotransferase; SLE, systemic lupus erythematosus; TSH, thyroid-stimulating hormone; PCSK9, proprotein convertase subtilisin/kexin type 9; PPARG, peroxisome proliferator activated receptor γ; SHBG, sex-hormone binding globulin; S.HDL.TG, Triglycerides in small HDL; M.VLDL.TG, Triglycerides in medium VLDL; PDGF-bb, platelet-derived growth factor BB. (DOCX) [file pmed.1004362.s013.docx]

| S8 Table. Overall comparison between meta-analyses and MR studies. | | | | | | | |
| --- | --- | --- | --- | --- | --- | --- | --- |
|  | **MR-Risk** | **MR-Protective** | **MR-null** | **MR-not available** | | | |
| Meta-Risk | height* | ------ | total dairy product  calcium*  birth weight  25-hydroxyvitamin D  CRP  UC | adult weight gain  prostatitis  infertility  BPH  melanoma  primary Sjögren's syndrome | acne in adolescence  androgenic alopecia* finasteride*  total cholesterol | sweetened beverage  egg consumption  serum folate  breast cancer in first degree family  processed meat | cobalt  asbestos  vasectomy  number of female partners  firefighter |
| Meta-Protective | ------ | smoking  PA | coffee  selenium*  vitamin E  schizophrenia  T2D | daidzein  HIV/AIDS  Parkinson's disease | aspirin  digoxin  finasteride  fat mass | soy consumption  occupational PA  age at first intercourse | tissue level linoleic acid |
| Meta-null | LDL  zinc | BMI | alcohol  sleep duration  sedentary behaviors  lycopene  adiponectin  hypothyroidism  asthma  DHA  CD | shift  metformin  genistein  equol  C peptide  hepatitis C  periodontitis  vasculitis  multiple sclerosis  cholelithiasis  bariatric surgery  subclinical hypothyroidism  white blood cell count | antagonist  statins  NSAID  cardiac glycoside  hypertension  obstructive sleep apnea  thiazolidinediones  sulfonylureas  insulin  pesticides  green space  arsenic  total protein intake  animal protein intake  plant protein intake | green tea  black tea  tomato  Mediterranean diet  dietary folate intake  total red meat  dietary linoleic acid  total nut intake  fruit  vegetable  vegetarian  pescatarian  dietary vitamin E intake  supplemental vitamin E intake | cadmium  ejaculation frequency  whole body vibration  farming  police  dairy protein intake  cruciferous vegetable intake  total fish  total milk  cheese  butter  yogurt  ice cream  dietary lycopene |
| Meta-not available | vitamin B12  TG  MUFAs  MDD  education attainment  LTL  aspartate  testosterone  IGF-1  IGF-II  IGFBP-3  circulating phosphorous  alanine  C-X-C motif chemokine ligand 9  CCL4  CCL2  TOR1AIP1  lactate  pyruvate  Glycoprotein acetyls, mainly a1-acid glycoprotein  creatinine  CCB  serum uric acid  age of sexual initiation  IL-6  PPARG  IL-6ra | serum iron  morning chronotype  puberty timing  HMG-CoA reductase  PCSK9  TSH  IGFBP-1  S.HDL.TG  PDGF-bb M.VLDL.TG  β-NGF  IDO 1  SCGF-β  MSP  albumin  hyperthyroidism  ALT  Class. Alphaproteobacteria  Order. Rhodospirillales  Genus. Adlercreutzia  Genus. Coprobacter  IL-1ra  SLE  atrial fibrillation | PUFA  homocystein  allergic disease  vitiligo  waist circumference  waist-hip ratio  SBP/DBP  plasma phospholipid arachidonic acid  tryptophan  kynurenine  serum urea  glutamate  fatty acids  pigmentation phototype  free thyroxine  fasting glucose  HbA1c (%) reduction  fasting insulin  SHBG  resistin  β-carotene  α-carotene  other null associations |  |  |  |  |

*represents advanced, aggressive, high-grade or lethal prostate cancer. Other null associations of biomarkers in MR studies were recorded in a previous review by Markozannes et al. (reference 19). Abbreviations: PA, physical activity; DHA, docosahexaenoic acids; EPA, eicosapentaenoic; HDL, high-density lipoprotein; LDL, low-density lipoprotein; CRP, C-reactive protein; T2D, type 2 diabetes; BPH, benign prostate hyperplasia; HIV, human immunodeficiency virus; AIDS, acquired immune deficiency syndrome; CD, Crohn's disease; UC, ulcerative colitis; AASVs, anti-neutrophil cytoplasm antibody associated vasculitides; ACEI, angiotensin converting enzyme inhibitors; NSAID, nonsteroidal anti-inflammatory drug; CCB, calcium channel blockers; TG, triglyceride; MUFAs, monounsaturated fatty acids; MDD, major depressive disorder; LTL, leukocyte telomere length; IGF, insulin-like growth factor; IGFBP, IGF-binding protein; TOR1AIP1, Torsin-1A-interacting protein 1; IL-6ra, IL-6 receptor subunit alpha; IDO 1, Indoleamine 2,3-dioxygenase 1; SCGF-β, stem cell growth factor-beta; β-NGF, beta nerve growth factor; MSP, microseminoprotein-beta; ALT, alanine aminotransferase; SLE, systemic lupus erythematosus; TSH, thyroid-stimulating hormone; PCSK9, proprotein convertase subtilisin/kexin type 9; PPARG, peroxisome proliferator activated receptor γ; SHBG, sex-hormone binding globulin; S.HDL.TG, Triglycerides in small HDL; M.VLDL.TG, Triglycerides in medium VLDL; PDGF-bb, platelet-derived growth factor BB.
