# Supplementary material for: Risk factors for prostate cancer: An umbrella review of prospective observational studies and mendelian randomization analyses
Source: PLoS Med. 2024 Mar 15;21(3):e1004362. doi: 10.1371/journal.pmed.1004362 (PMC10980219; doi:10.1371/journal.pmed.1004362)
Supplement: Attachment — Submitted filename: PMEDICINE-D-23-01730R1-Point_by_Point_Responses to Reviewers.doc [file pmed.1004362.s014.doc]

**Authors’ Reponses to the Editors’ and Reviewers’ Comments**

(PMEDICINE-D-23-01730R1)

We wish to thank the editors and reviewers for their valuable comments and suggestions regarding our manuscript (PMEDICINE-D-23-01730R1). We have revised the manuscript point by point as requested. Literature search has been updated to September 9, 2023, obtaining 83 additional articles (51 meta-analyses and 32 Mendelian randomization studies) published after our initial search, and the results have been expanded and updated after managing the additional studies. We have also made changes to improve the format of the manuscript to comply with the journal’s style. Our point-by-point responses (R) to each specific comment (Q) are provided below. All revisions are presented here as excerpts of revised text with quotation marks to facilitate the review process.

**Requests from the editors:**

• General comments:

Please include line numbers in your revised manuscript, ideally not starting from 1 with each new page.

R: Thank you for the kind suggestion. We have included continuous line numbers throughout the revised manuscript.

Please consider having your manuscript reviewed by someone with full professional proficiency in English.

R: Thank you for the kind suggestion. We have invited an experienced writer with full professional proficiency in English to review and improve the writing.

Please replace "Caucasian" with "white" throughout the paper.

R: Thank you for the suggestion. We have replaced “Caucasian” with “white” throughout the paper.

Please revise ‘sweeten beverage’ to ‘sweetened beverage’

R: Thank you for the suggestion. We have revised “sweeten beverage” to “sweetened beverage”.

Throughout the paper, please adapt reference call-outs to the following style: "... every year [1,2]." (noting the absence of spaces within the square brackets).

R: Thank you for the kind suggestion. We have adapted the reference call-outs to the style as “… [1,2].”

• Title: Please revise your title according to PLOS Medicine's style. We suggest “Risk factors for prostate cancer: An umbrella review of evidence from observational studies and Mendelian Randomization studies” or similar

R: Thank you for the kind suggestion. As suggested, we have changed the title into “Risk factors for prostate cancer: An umbrella review of evidence from prospective observational studies and Mendelian randomization studies”.

• Abstract:

Please report your abstract according to PRISMA for abstracts, following the PLOS Medicine abstract structure (Background, Methods and Findings, Conclusions) <http://www.plosmedicine.org/article/info:doi/10.1371/journal.pmed.1001419>

R: We thank the editors for the suggestion. We have reported the abstract according to PRISMA for abstracts, following the PLOS Medicine abstract structure (Background, Methods and Findings, Conclusions) in the revised manuscript.

• Abstract Background: Please expand on the context of why the study is important.

R: Thank you for the suggestion. We have expanded on the context of why the study is important as suggested.

It now reads “The incidence of prostate cancer is increasing in older males globally. Age, ethnicity, and family history are identified as the well-known risk factors for prostate cancer, but few modifiable factors have been firmly established” in the revised manuscript.

Please revise ‘rising’ for clarity; such as ‘The incidence of prostate cancer is increasing in older males’

R: Thank you for the suggestion. We have revised “rising” for clarity as suggested.

It now reads “The incidence of prostate cancer is increasing in older males” in the revised manuscript.

Please remove ‘perplexing’

Please remove ‘aging elder’

Please remove “apart from race, age, and heredity”, which are not modifiable risk factors.

R: Thank you for the suggestions. We have removed “perplexing”, “aging elder”, and “apart from race, age, and heredity”.

The final sentence should clearly state the study question.

R: Thank you for the suggestion. We have revised the final sentence to clearly state the study question.

It now reads “The objective is to identify and evaluate various factors modifying the risk of prostate cancer reported in meta-analyses of prospective observational studies and Mendelian randomization (MR) analyses” in the revised manuscript.

Please revise to ‘prospective observational studies’

Please revise to ‘Mendelian’

R: Thank you for the suggestion. We have revised to “prospective observational studies” and “Mendelian” as suggested.

• Abstract Methods and Findings:

Please provide the dates of search (start and end), number of studies included, eligibility criteria, and synthesis/appraisal methods.

R: Thank you for the suggestion. We have provided the dates of search (start and end), number of studies included, eligibility criteria, and synthesis/appraisal methods as suggested.

It now reads “We searched PubMed, Embase, and Web of Science from the inception to January 10, 2022, updated on September 9, 2023, to identify meta-analyses and MR studies on prostate cancer. Eligibility criteria were 1) meta-analyses including prospective observational studies or studies that declared outcome-free at baseline; 2) evaluating the factors of any category associated with prostate cancer incidence; 3) providing effect estimates for further data synthesis. Similar criteria were applied to MR studies. Quality assessment was then conducted for included meta-analyses using AMSTAR tool and for MRs using STROBE-MR and assumption evaluation. Subsequent evidence grading criteria for significant associations in meta-analyses contained sample size, P-values and 95% confidence intervals, 95% prediction intervals, heterogeneity, and publication bias, assigning four evidence grades (convincing, highly suggestive, suggestive, or weak). Significant associations in MR studies were graded as robust, probable, suggestive, or insufficient considering P-values and concordance of effect directions” in the revised manuscript.

Please quantify the main results presented in the abstract

R: Thank you for the suggestion. We have quantified the main results presented in the abstract.

It reads “Finally, 92 selected from 411 meta-analyses and 64 selected from 118 MR studies were included after excluding overlapping and outdated studies. In total, 123 observational associations (46 significant and 77 null) and 145 causal associations (55 significant and 90 null) were categorized into lifestyle, diet and nutrition, anthropometric indices, biomarkers, medical conditions, and environmental factors. Concerning evidence grading on significant associations, there were five highly suggestive, 37 suggestive, and four weak associations in meta-analyses, and 10 robust, 24 probable, four suggestive, and 17 insufficient causal associations in MR studies. Twenty-six overlapping factors between meta-analyses and MR studies were identified, with consistent significant effects found for physical activity (PA) (occupational PA in meta: OR=0.87, 95% CI: 0.80-0.94; accelerator-measured PA in MR: OR=0.49, 95% CI: 0.33-0.72), height (meta: OR=1.09, 95% CI: 1.06-1.12; MR: OR=1.07, 95% CI: 1.01-1.15, for aggressive prostate cancer), and smoking (current smoking in meta: OR=0.74, 95% CI: 0.68-0.80; smoking initiation in MR: OR=0.91, 95% CI: 0.86-0.97)” in the revised manuscript.

Please revise ‘In total, 94 associations…’

R: Thank you for the suggestion. We have revised into “In total, 123 associations…” in the revised manuscript because of the updated results.

In the last sentence of the Abstract Methods and Findings section, please describe 2-3 of the main limitations of the study's methodology.

R: Thank you for the suggestion. We have described the main limitations of the study’s methodology in the last sentence of the Abstract Methods and Findings section.

It now reads “Methodological limitation is that the evidence grading criteria could be expanded by considering more indices” in the revised manuscript.

• Abstract Conclusions:

Please begin your Abstract Conclusions with "In this study, we observed ..." or similar, to summarize the main findings from your study, without overstating your conclusions. Please emphasize what is new and address the implications of your study, being careful to avoid assertions of primacy. Please avoid vague statements such as "significant relevance for public health".

R: Thank you for the suggestion. In the revised manuscript, we have revised the sentences in Conclusions according to your suggestions.

It now reads “In this comprehensive study, we summarized associations between various factors with the risk of prostate cancer and found that height, physical activity, and smoking had consistently significant effect on the risk of prostate cancer between meta-analyses and MR studies, adding to our understanding on the etiology of prostate cancer” in the revised manuscript.

• Author Summary:

At this stage, we ask that you include a short, non-technical Author Summary of your research to make findings accessible to a wide audience that includes both scientists and non-scientists. The Author Summary should immediately follow the Abstract in your revised manuscript. This text is subject to editorial change and should be distinct from the scientific abstract. Please see our author guidelines for more information: [https://journals.plos.org/plosmedicine/s/revising-your-manuscript#loc-author-summary](https://journals.plos.org/plosmedicine/s/revising-your-manuscript#loc-author-summary)

In the final bullet point of ‘What Do These Findings Mean?’, please describe the main limitations of the study in non-technical language.

R: We thank the editors for the detailed suggestions. We have added the Author Summary as requested.

**Author Summary**

**Why was this study done?**

•	The incidence of prostate cancer is increasing with the growing trend of aging globally.

•	Effective preventions and interventions for prostate cancer require better understandings of its etiology.

•	The well-known risk factors for prostate cancer are age, ethnicity, and family history, but few modifiable factors have been firmly established.

**What did the researchers do and find?**

•	Our study extensively collected, evaluated, and compared the current observational and genetic evidence for various factors modifying the risk of prostate cancer based on meta-analyses and Mendelian randomization (MR) studies.

•	Totally 123 observational associations (46 significant and 77 null) from 92 meta-analyses and 145 causal associations (55 significant and 90 null) from 64 MRs were identified and categorized into lifestyle, diet and nutrition, anthropometric indices, biomarkers, medical conditions, and environmental factors.

•	Concerning evidence grading on significant associations, there were five highly suggestive, 37 suggestive, and four weak associations in meta-analyses, and 10 robust, 24 probable, four suggestive, and 17 insufficient causal associations in MR studies.

•	Consistent significant associations between meta-analysis and MR studies were found for physical activity, height, and smoking.

**What do these findings mean?**

•	Most included cohort studies were conducted in developed western countries, and hence the findings in this study are limited for mainly European descendants.

•	Evidence grading criteria both for meta-analyses and MRs could be expanded, for example, considering magnitude of effect size and different levels of sample size.

•	Weak and insufficient evidence identified in this umbrella review warrant further investigations.

• Introduction:

Please temper assertions of primacy by adding ‘to the best of our knowledge’ or similar, in the following sentences:

“...remain unevaluated by umbrella reviews.”

“...have rarely been compared…”

“...accumulating new and unevaluated…”

“...sheds novel lights on PrCa oncogenesis…”

R: Thank you for the suggestions. We have tempered assertions of primacy in the above-mentioned sentences.

Please remove “bears significant public health and clinical relevance.”

R: Thank you for the suggestion. We have removed “bears significant public health and clinical relevance.”

Please conclude the Introduction with a clear description of the study question or hypothesis.

R: Thank you for the suggestion. We have concluded the Introduction with a clear description of the study question or hypothesis.

It now reads “Therefore, the objective of this umbrella review is to identify and evaluate various factors modifying the risk of prostate cancer reported in meta-analyses of prospective observational studies and MR studies, to better understand the etiology of prostate cancer” in the revised manuscript.

• Methods:

Did your study have a prospective protocol or analysis plan? Please state this (either way) early in the Methods section. If a prospective analysis plan was used in designing the study, please include the relevant prospectively written document with your revised manuscript as a Supporting Information file to be published alongside your study, and cite it in the Methods section. If no such document exists, please make sure that the Methods section transparently describes when analyses were planned, and if/when reported analyses differed from those that were planned. Changes in the analysis-- including those made in response to peer review comments-- should be identified as such in the Methods section of the paper, with rationale. If a reported analysis was performed based on an interesting but unanticipated pattern in the data, please be clear that the analysis was data-driven.

R: Thank you for your suggestions. We are sorry that we did not have a prospective protocol, so no such relevant document can be provided in the Supporting Information file. This large-scale umbrella review was initially planned to focus on evidence from observational studies, so the initial search was conducted on January 10, 2022 only for meta-analyses. As Mendelian randomization studies provided important genetic evidence, another search for MRs was conducted on July 6, 2022.

According to your kind suggestions, we have stated this early in the Methods section and transparently described when the analyses were planned and if/when reported analyses differed from what were planned. Changes in the analysis in response to peer review comments have also been reported in the Methods section, with rationale. Rationales of analyses with a clear time line were transparently reported in the Methods section.

It now reads “No pre-registered study protocol is available. This umbrella review was initially planned to focus on evidence from observational studies, so the initial search was conducted on January 10, 2022 only for meta-analyses. An additional search for MR studies was later conducted on July 6, 2022, to include the important genetic evidence from MR studies. Upon request, the literature search for meta-analyses and MR studies was updated on September 9, 2023” in the revised manuscript.

Please add the following statement, or similar, to the Methods: "This study is reported as per the Preferred Reporting Items for Systematic Reviews and Meta-Analyses (PRISMA) guideline (S1 Checklist)."

R: Thank you for the suggestion. In the revised manuscript, we have added this sentence to the Methods. It is now the first sentence in Methods section and it reads “This study is reported as per the Preferred Reporting Items for Systematic Reviews and Meta-Analyses (PRISMA) guideline (S1 Checklist)”.

We require that SRs are updated to within roughly 6 months of the expected publication date. Please update your search to the present time.

R: Thank you for the suggestion. We have updated the search till September 9, 2023, obtaining 83 additional articles (51 meta-analyses and 32 MR studies) published after our initial search, and the results have been expanded and updated after managing the additional studies in the revised manuscript.

Please consider including non-English language sources of studies.

R: Thank you for the kind suggestion. As suggested, we included the non-English language sources of study in our updated literature search and screening, only to find that there were 208 non-English papers and none remained eligible after screening. Still, we have removed the language restriction in previous inclusion criteria in the revised manuscript.

Please refer to specific files in your supplementary information e.g. S1 Table, S1 Figure, etc. including for the search strategy

R: Thank you for the suggestion. We have referred to the specific files in the supplementary information as requested.

• Results:

Please define all abbreviations at first use e.g. OR, RR, DHA, EPA, BMI, T2D, etc

R: Thank you for the suggestion. We have defined all abbreviations at first use as requested including OR, RR, DHA, EPA, BMI, T2D, etc.

Where ORs are presented, please specify the comparison group.

R: Thank you for the suggestion. We have specified the comparison group where ORs are presented.

Regarding birth weight (OR=1.02, 95% CI: 1.00-1.05) and fat mass (OR=0.88, 95% CI: 0.76-1.00): Please clarify why these are considered significant since they contain the null value

R: Thank you for the question. The reason why these two factors were considered significant is that their P values were both 0.045 (less than the significance threshold of 0.05 in this umbrella review), which was also claimed to be significant in the original meta-analysis. Therefore, birth weight and fat mass were considered significant in our umbrella review.

“well documented in the previous review” - please provide a citation

R: Thank you for the suggestion. We have added a citation for this statement “well documented in the previous review”.

In the ‘lifestyle’ paragraph you state that “Null associations were found between PrCa and 11 lifestyle factors: … physical activity…” but later (on page 26) you state that “physical activity (evidence level: convincing in meta-analysis /insufficient in MR)” - please clarify.

R: Thank you for pointing out the inconsistency, and we are sorry for the confusion. There are several different indices for physical activity (PA) based on existing literature: overall PA (null association in meta-analysis), occupational PA (significant association in meta-analysis, grade: highly suggestive), and accelerometer-measured PA (significant in MR, grade: insufficient). To avoid confusion, we have clearly distinguished these three different indices in the revised manuscript.

• Discussion:

Please present and organize the Discussion as follows: a short, clear summary of the article's findings; what the study adds to existing research and where and why the results may differ from previous research; strengths and limitations of the study; implications and next steps for research, clinical practice, and/or public policy; one-paragraph conclusion.

R: Thank you for the kind instructions. We have revised the Discussion as requested. Please see the revised Discussion section.

Please remove all subheadings within your Discussion e.g. Meta-analyses

R: Thank you for the suggestion. We have removed all subheadings within the Discussion.

Please temper assertions of primacy by adding ‘to the best of our knowledge’ or similar e.g. “the hitherto most comprehensive appraisal “ etc

R: Thank you for the suggestion. We have tempered assertions of primacy by adding ‘to the best of our knowledge’ or similar expressions as suggested.

As before, please clarify your classification in the Discussion of physical activity as convincing/highly suggestive when this was not the result presented in the Results.

R: Thank you for pointing out the inconsistency, and we are sorry for the confusion. There are several different indices for physical activity (PA) based on existing literature: overall PA (null association in meta-analysis), occupational PA (significant association in meta-analysis, grade: highly suggestive), and accelerometer-measured PA (significant in MR, grade: insufficient). To avoid confusion, we have clearly distinguished these three different indices in the revised manuscript.

“four robust associations in MR” - please note that only 3 associations are listed

R: Thank you for pointing out the inconsistency. Because of the updated literature search as required, the results have also been different from previous ones. Now there are 10 robust associations in MR.

Please revise “...confirming the roles of…”, “protective effects of”, “highlighted the role of”, “Higher PrCa incidence was found in”, “provides novel prospects”, “the most comprehensive one”, “providing novel insights“ etc. to avoid use of causal language

R: Thank you for the kind suggestions. We have revised these phrases to avoid use of causal language in the revised manuscript.

• Figures:

Please define abbreviations used in the figure legend of each individual figure.

R: Thank you for the suggestion. We have defined abbreviations used in the figure legend of each individual figure as requested. Please see in the revised files.

Fig 4: When a p value is given, please specify the statistical test used to determine it in the figure legend.

R: Thank you for the suggestion. We have specified the statistical test used to determine the p value in the figure legend of figures with P value reported.

• Tables:

Please define all abbreviations used in the table legend of each individual table.

R: Thank you for the suggestion. We have defined all abbreviations used in the table legend of each individual table as requested.

Supplementary Table 1 - please indicate which exposure is being investigated for each of the sections e.g. Burton 2021 is the most recent of three papers, but what was the exposure of interest in these three? Please also indicate why some names are emboldened, as this doesn’t always appear to correspond to the paper used in your review, and the meaning of the square root symbol / “latest from MR”.

R: We are sorry for the confusions in the table. 1) The exposure factors are listed in the very first column as “Exposure”. 2) The reason why it has so many confusions including that the emboldened names were usually not selected ones is that we have mistakenly submitted the unfinished version of table 1 in the initial submission, which contained a lot of undecided results. We are sorry that we have not dealt with this table properly before the submission. 3) the square root symbol / “latest from MR” was an uncorrected error, and we have corrected it.

Tables 2 & 3: When a p value is given, please specify the statistical test used to determine it in the table legend.

R: Thank you for the suggestion. We have specified the statistical test used to determine the P value in the table legend.

• References:

Please ensure that journal name abbreviations match those found in the National Center for Biotechnology Information (NCBI) databases (http://www.ncbi.nlm.nih.gov/nlmcatalog/journals), and are appropriately formatted and capitalised.

Please also see https://journals.plos.org/plosmedicine/s/submission-guidelines#loc-references for further details on reference formatting.

Where website addresses are cited, please specify the date of access.

R: We thank the editors for the detailed instructions. We have followed the instructions on [https://journals.plos.org/plosmedicine/s/submission-guidelines#loc-references](https://journals.plos.org/plosmedicine/s/submission-guidelines#loc-references) and revised the reference formatting accordingly.

• Supplementary files:

Please provide titles and legends for each individual table and figure in the Supporting Information.

Please see https://journals.plos.org/plosmedicine/s/supporting-information for our supporting information guidelines.

R: Thank you for the instruction. We have provided titles and legends for each individual table and figure in the Supporting Information.

Reviewer #1: This is a well-conducted umbrella review on the factors modifying prostate cancer risk reported in meta-analyses of prospective studies as well as mendelian randomization studies. The study design, datasets, statistical methods and analyses, and presentation (tables and figures) and interpretation of the results are mostly adequate and of a good standard. However, still a couple of issues needing attention.

1) Table 2 summarises the 72 meta-analyses included in the review. We can see quite a few studies including both Caucasian and Asian cohorts. As it's known the race plays a big role in prostate cancer, it would be very useful if authors could do some subgroup analyses to show whether the claimed risk factors differ, at least between Caucasian and Asian cohorts. I am aware the authors have aknowledged this in limitations citing there are not enough data on race but it seems that data on both Caucasian and Asian are avaiable so it might be possible to conduct a subgroup analysis on this.

R: We thank the reviewer for the kind suggestion, and we agree with the reviewer that subgroup analyses are useful and valuable to distinguish at least between the Caucasians and the Asians. There are indeed Asian cohorts included for some studies, however, the number of included Asian cohorts are usually less than 2, and the maximum number is 4 for smoking. Even so, as suggested we revisited the data and performed subgroup analyses for exposure factors with available data (table shown below). To sum up, the subgroup analyses results showed the effects remained largely consistent for Caucasian cohorts. We have added a brief description of subgroup analyses in the revised manuscript.

It now reads “Additionally, subgroup analyses between whites and non-whites population were performed upon request. The subgroup analyses results showed the effects remained largely consistent for white cohorts (S8 Table) and that number of researches on non-white population was very limited” in the revised manuscript.

**Table 1. Subgroup analysis between whites and non-whites.**

| **Exposure** | **Overall effect size OR(95%CI)** | **effect size in whites OR(95%CI)** | **effect size in non-whites OR(95%CI)** |
| --- | --- | --- | --- |
| aspirin | 0.92 (0.87-0.97) | 0.92 (0.86-0.98) | 0.95 (0.88-1.03) (N=1) |
| calcium | 1.02 (1.01-1.04) | 1.02 (1.01-1.04) | 1.16 (0.88-1.53) (N=1) |
| coffee | 0.91 (0.84-0.98) | 0.91 (0.84-0.98) | 0.85 (0.48-1.51) (N=2) |
| current smoking | 0.74 (0.67-0.81) | 0.74 (0.67-0.83) | 0.67 (0.60-0.75) (N=4) |
| daidzein | 0.75 (0.60-0.93) | 0.72 (0.52-1.00) (N=2) | 0.77 (0.51-1.16) (N=2) |
| finasteride | 0.70 (0.52-0.95) | 0.72 (0.49-1.06) | 0.67 (0.61-0.73) (N=2) |
| firefighter | 1.21 (1.12-1.32) | 1.21 (1.12-1.32) | 1.32 (0.65-2.69) (N=1) |
| height | 1.09 (1.06-1.12) | 1.09 (1.06-1.12) | 1.12 (0.89-1.41) (N=1) |
| soy | 0.90 (0.82-0.98) | 0.90 (0.82-1.00) | 0.86 (0.66-1.11) (N=2) |
| total dairy products | 1.04 (1.00-1.07) | 1.03 (1.00-1.06) | 1.63 (1.14-2.33) (N=1) |
| ulcerative colitis | 1.23 (1.03-1.46) | 1.15 (1.00-1.31) | 1.93 (0.86-4.33) (N=1) |

(A) Aspirin

(B) Calcium

(C) coffee

(D) current smoking

(E) Daidzein

(F) Finasteride

(G) Firefighter

(H) Height

(I) Soy

(J) Total dairy products

(K) Ulcerative colitis

2) Figure 3 is very informative. The evidence grading is very useful. However, it would be good to add the I-squared value for heterogeneity too in the figure as it's a part of routine meta-analysis and familar to readers and easy to follow. The Odds ratio, I-squared and evidence grading would make a comprehensive presentation of the results.

R: Thank you for the kind suggestion, and we agree with the reviewer that odds ratio, I-squared and evidence grading would make a comprehensive presentation of the results. As suggested, we have added the I-squared values for heterogeneity plus P values of the effect estimates in Figure 3 for readers to better comprehend the results.

Reviewer #2: Cui et al. have put together a comprehensive umbrella review of risk factors associated with prostate cancer via meta-analyses of prospective observational studies and Mendelian randomization studies.

Major comments

1. There is some lack of novelty given two umbrella reviews published by Markozannes, which overlap with the work here (2016 European Journal of Cancer, 2022 BMC Medicine). Still, the current work appears comprehensive and might still be additive to the literature.

R: We thank the reviewer for the kind comments. The two prior umbrella reviews by Markozannes et al. (PMID: 27816833, 35105367) have done solid work in investigating prostate cancer. Our work, as you have mentioned, obviously has the strength of comprehensiveness based on updated evidence and expanded categories. The existing umbrella review by Markozannes et al. in 2016 based on literature published up to April 30, 2013, while in our umbrella review all the included meta-analyses for data synthesis were published after 2014, of which around 60% were published after 2020 (N=53), presenting updated evidence for each factor. Secondly, the previous umbrella review studied associations of 23 foods, 31 nutrients, eight indices of body size and three indices of physical activity, while our umbrella review greatly expanded the categories of risk factors by containing six categories covering lifestyle, diet and nutrition, anthropometric indices, medical conditions, biomarkers, and environmental factors, bringing the total number of studied factors to over 120. Furthermore, this umbrella review reported medical conditions including pre-existing diseases, medication, and surgery, which was often neglected in previous reviews. Last but not the least, the unique strength of this umbrella review is the comparison between meta-analyses of prospective observational studies and MR studies. Integrating epidemiological evidence and MR causal inference, with the former providing the foundation for MR causal exploration while MR helping verify the causality in turn, provides useful insights in examining intrinsic relationships. Collectively, we believe our work is comprehensive and additive to the literature.

2. Authors should add information on the exclusion criteria for meta-analyses and Mendelian randomization studies in the Methods section.

R: We thank the reviewer for the kind suggestions. As suggested, we have added information on the exclusion criteria for meta-analyses and Mendelian randomization studies in the Methods section.

It now reads: “The exclusion criteria were as follows: 1) meta-analyses including only retrospective studies; 2) narrative reviews or reviews without data synthesis results or failing to provide sufficient data for calculation; 3) the outcome of interest was the diagnosis, treatment, or prognosis.”

3. In meta-analyses, among the dietary and nutritional factors, authors report that circulating high levels of 25-hydroxyvitamin D associated with PrCa risk. Was the cut-off level defining high or low was same in the meta-analysis review?

R: We thank the reviewer for pointing out this important question. We have reviewed the selected meta-analysis on 25-hydroxyvitamin D and paid special attention to the cut-off level. The cut-off values varied among each individual study, and it is thus not likely to use the same cut-off level defining high or low across the studies. Please see the details in the following table. The effect estimates the investigator chose for data synthesis were basically those represented the highest level (mostly > 35ng/ml).

**Table 2. Cut-off values defining 25-hydroxyvitamin D levels in the original meta-analysis.**

| **References** | **Serum/plasma 25(OH)D** | **Estimate effects** |
| --- | --- | --- |
| Braun et al. ([1995](https://link.springer.com/article/10.1007/s00432-014-1706-3#ref-CR13)) | ng/ml | OR (95 % CI) |
|  | <24.1 | 1 |
|  | 24.1–29.5 | 2.30 (0.70–7.80) |
|  | 29.6–35.4 | 2.30 (0.70–7.70) |
|  | 35.5-41.3 | 0.60 (0.10–2.50) |
|  | **>41.3** | **2.40 (0.80–8.20)** |
| Ma et al. ([1998](https://link.springer.com/article/10.1007/s00432-014-1706-3#ref-CR34)) | Quantile | OR (95 % CI) |
|  | Low | 1 |
|  | **High** | **1.12 (0.81–1.54)** |
| Nomura et al. ([1998](https://link.springer.com/article/10.1007/s00432-014-1706-3#ref-CR41)) | ng/ml | OR (95 % CI) |
|  | <34 | 1 |
|  | 34–40 | 0.80 (0.40–1.80) |
|  | **41–47** | **0.80 (0.40–1.70)** |
|  | ≥48 | 0.80 (0.40–1.80) |
| Ahonen et al. ([2000](https://link.springer.com/article/10.1007/s00432-014-1706-3#ref-CR3)) | ng/ml | OR (95 % CI) |
|  | >22 | 1 |
|  | 16.4–22 | 0.80 (0.50–1.50) |
|  | 12.4–16.3 | 1.40 (0.80–2.40) |
|  | **<12.4** | **1.80 (1.00–3.20) inverse** |
| Jacobs et al. ([2004](https://link.springer.com/article/10.1007/s00432-014-1706-3#ref-CR29)) | ng/ml | OR (95 % CI) |
|  | 8.10–25.3 | 1 |
|  | 25.4–32.7 | 1.71 (0.68–4.34) |
|  | **32.8–59.7** | **0.75 (0.29–1.91)** |
| Platz et al. ([2004](https://link.springer.com/article/10.1007/s00432-014-1706-3#ref-CR46)) | Quartile | OR (95 % CI) |
|  | Q1 (low) | 1 |
|  | Q2 | 1.00 (0.67–1.49) |
|  | Q3 | 0.77 (0.51–1.15) |
|  | **Q4 (high)** | **1.19 (0.79–1.79)** |
| Tuohimaa et al. ([2004](https://link.springer.com/article/10.1007/s00432-014-1706-3#ref-CR56)) | ng/ml | OR (95 % CI) |
|  | ≤7.6 | 1.50 (0.80–2.70) |
|  | 7.7–15.6 | 1.30 (0.98–1.60) |
|  | 15.7–23.6 | 1 |
|  | 23.7–31.6 | 1.20 (0.90–1.50) |
|  | **≥31.7** | **1.70 (1.10–2.40)** |
| Li et al. ([2007](https://link.springer.com/article/10.1007/s00432-014-1706-3#ref-CR32)) | Quartile | OR (95 % CI) |
|  | **Q1 (low)** | **1.01 (0.71–1.44) inverse** |
|  | Q2 | 1.26 (0.89–1.80) |
|  | Q3 | 1.00 (0.71–1.41) |
|  | Q4 (high) | 1 |
| Mikhak et al. ([2007](https://link.springer.com/article/10.1007/s00432-014-1706-3#ref-CR37)) | Quantile | OR (95 % CI) |
|  | Sufficient | 1 |
|  | **Deficient** | **0.62 (0.43–0.91) inverse** |
| Ahn et al. ([2008](https://link.springer.com/article/10.1007/s00432-014-1706-3#ref-CR1)) | ng/ml | OR (95 % CI) |
|  | 5.12–17 | 1 |
|  | 17.1–20.5 | 1.10 (0.78–1.56) |
|  | 20.6–24.2 | 1.53 (1.10–2.13) |
|  | 24.3–28.7 | 1.33 (0.95–1.86) |
|  | **28.8–51.8** | **1.18 (0.83–1.68)** |
| Travis et al. ([2009](https://link.springer.com/article/10.1007/s00432-014-1706-3#ref-CR55)) | ng/ml | OR (95 % CI) |
|  | 1–16.2 | 1 |
|  | 16.3–20.2 | 1.27 (0.89–1.81) |
|  | 20.3–23.6 | 1.23 (0.85–1.76) |
|  | 23.7–28.3 | 1.06 (0.73–1.55) |
|  | **28.4–65.5** | **1.28 (0.88–1.88)** |
| Park et al. ([2010](https://link.springer.com/article/10.1007/s00432-014-1706-3#ref-CR43)) | ng/ml | OR (95 % CI) |
|  | <22.9 | 1 |
|  | 22.9–<31.0 | 1.05 (0.70–1.58) |
|  | 31.0–<39.9 | 0.81 (0.52–1.28) |
|  | **≥39.9** | **1.17 (0.72–1.89)** |
| Albanes et al. ([2011](https://link.springer.com/article/10.1007/s00432-014-1706-3#ref-CR5)) | Residual units | OR (95 % CI) |
|  | ≤3.00 | 1 |
|  | 3.00–3.34 | 1.17 (0.87–1.57) |
|  | 3.34–3.63 | 1.12 (0.84–1.51) |
|  | 3.63–3.92 | 1.34 (0.99–1.82) |
|  | **>3.92** | **1.36 (1.01–1.82)** |
| Brandstedt et al. ([2012](https://link.springer.com/article/10.1007/s00432-014-1706-3#ref-CR12)) | ng/ml | OR (95 % CI) |
|  | ≤27.2 | 1 |
|  | 27.3–33.6 | 1.25 (0.95–1.65) |
|  | 33.7–40.8 | 1.37 (1.03–1.82) |
|  | ≥40.9 | 1.34 (0.99–1.82) |
| Gilbert et al. ([2012](https://link.springer.com/article/10.1007/s00432-014-1706-3#ref-CR25)) | ng/ml | OR (95 % CI) |
|  | **≥30** | **1.05 (0.84–1.32)** |
|  | 20–29.9 | 1 |
|  | 12–19.9 | 1.14 (0.93–1.38) |
|  | <12 | 1.08 (0.79–1.50) |
| Shui et al. ([2012](https://link.springer.com/article/10.1007/s00432-014-1706-3#ref-CR49)) | ng/ml (median) | OR (95 % CI) |
|  | 16.2 | 1 |
|  | 22.7 | 0.93 (0.74–1.17) |
|  | 27.4 | 0.99 (0.79–1.24) |
|  | **35.7** | **1.07 (0.86–1.34)** |
| Meyer et al. ([2013](https://link.springer.com/article/10.1007/s00432-014-1706-3#ref-CR36)) | ng/ml | RR (95 % CI) |
|  | <12 | 0.82 (0.58–1.15) |
|  | 12–20 | 1.02 (0.87–1.21) |
|  | 20–27.9 | 1 |
|  | 28–35.9 | 1.24 (1.05–1.47) |
|  | **≥36** | **1.17 (0.93–1.48)** |
|  | Per 30 increase | 1.13 (1.02–1.25) |

4. Also in meta-analyses and in MR studies, smoking was coming out to be a significant protective factor among lifestyle factors which is strange. Authors should add a paragraph in discussion section about this.

R: We thank the reviewer for the kind suggestion. We are also very interested in the finding on smoking, and we agree with the reviewer that more discussions should be added.

It now reads: “Smoking, albeit with consistent effect, should be taken prudently for the observed effect was moderate and mixed and that positive association in earlier years (before 1995) and with mortality collectively suggested a link to aggressive prostate cancer rather than indolent one. Current smoking was believed to be associated with a lower likelihood of prostate-specific antigen (PSA) testing, and individuals with a smoking history were less likely to undergo prostate biopsy. Consequently, the detection rate of prostate cancer could be relatively lower among participants in the PSA screening era. Another possible explanation is that smoking is the leading risk factor for death among males. Smokers may die from smoking attributable diseases including cancers, cardiovascular diseases, and respiratory diseases before their diagnosis of prostate cancer. The poor adherence of smokers to cancer screening and the occurrence of smoking-related aggressive cancers as well as cardiovascular, pulmonary, and several other deadly diseases may explain the negative association. Measures should be taken to help smokers to be more compliant with early cancer screening and to quit smoking” in the revised manuscript.

5. Among the environment factors, authors did not find a significant association of pesticides with PrCa in meta-analysis review which is not in line with the literature (PMID: 27244877).

R: We thank the reviewer for pointing out the inconsistency. We have read the article published in 2016 by Silva et al. (PMID: 27244877) carefully and compared its findings with the selected meta-analysis on pesticides published in 2019 by Krstev et al. (PMID: 31360689) in our umbrella review. Firstly, we select the meta-analysis by Krstev et al., considering that it is more updated and the data are more appropriately handled after comparison. Silva et al. included 49 articles (15 case-control studies and 34 cohort studies). Among the 34 analyzed cohort studies, of which 29 used data from the agricultural health study (AHS), a large cohort of more than 50,000 farmers and pesticide applicators from the United States which analyzed possible associations between specific pesticide exposure and many types of cancer. Twenty-seven of these studies analyzed exposure to a single substance, but as the discussion points out that “farmers and pesticide applicators are infrequently exposed to a single pesticide, and as there are numerous pesticides, most studies cannot determine the contribution of specific compounds”. While in the article by Krstev et al., it stated that the study with the longest follow-up period was selected if more than one article was published for the same cohort. Secondly, in the selected study by Krstev et al., the overall effect of pesticides on prostate cancer based on both cohort and case-control studies appeared significant (OR=1.15, 95%CI: 1.01-1.32), however, the cohort study reported a null association (OR=1.13, 95%CI: 0.97-1.32). Similarly, the positive association found by Silva et al. was also based on both case-control studies and cohort studies. Therefore, the inconsistent results regarding the effect of pesticides on prostate cancer warrant further investigations.

6. When comparing association between overlapping factors between meta-analyses and MR studies, only three factors showed consistently significant associations: height, smoking, and physical activity. How do these associations compare with the other umbrella reviews on prostate cancer, i.e., Markozannes et al. European Journal of Cancer, 2016?

R: Thank you for your question. Regarding height, Markozannes et al. (PMID: 27816833) found that the association of height with total prostate cancer incidence and mortality presented highly suggestive evidence with a 4% higher risk per 5 cm greater height (95%CI: 1.03-1.05), which is consistent with our study (height: per 10 cm increase, OR=1.09, 95%CI: 1.06-1.12, suggestive). In addition, significant associations with advanced prostate cancer were found for height in both studies. Concerning physical activity, Markozannes et al. found that occupational activity showed inverse association with prostate cancer incidence and mortality (highest vs lowest, OR=0.87, 95%CI: 0.80-0.95, weak), and our study found that occupational physical activity (OR=0.87, 95%CI: 0.80-0.94, highly suggestive). Both studies found that there was no significant association between total physical activity with prostate cancer risk. Generally, the results from the two reviews are consistent concerning height and physical activity. Smoking was not investigated in the previous review, so there is no comparison available for smoking.

Reviewer #3: In their manuscript, entitled "Understanding the risk of prostate cancer: an umbrella review of observational and genetic evidence," authors Cui et al. attempt to make sense of the large collection of environmental exposure associations with prostate cancer. To do so, they conducted a search of all meta-analyses of prospective studies from 1990-2022 published in the English language, assigning evidence grades (convincing, highly suggestive, suggestive, or weak) to correlations detected. Grades reflected on sample size, P-values and 95% confidence intervals, Funnel plot asymmetry, etc. Mendelian randomization studies were graded as robust, probable, suggestive, and insufficient considering P-values and concordance of effect directions.

Findings were: (i) selection of 72 meta-analyses reporting 94 associations (from 360 or so identified studies), predominantly sampling Caucasians from Europe and North America, (ii) 41 significant associations subjected to grade assignment, with only 12 associations showing a 95% confidence interval that excluded a null association, (iii) significant correlations between diet and nutrition and prostate cancer for 11 factors, with highly suggestive evidence for sweetened beverages and circulation vitamin D, (iv) convincing evidence for reduced physical activity and prostate cancer, with suggestive evidence for associations of birth weight, height, and fat mass and prostate cancer, (v) only suggestive evidence of disease impact from medical conditions and/or medications, (vi) suggestive evidence for C-reactive protein levels and prostate cancer risk, (vii) highly suggestive evidence for asbestos exposure and disease risk, (viii) 18 associations discriminated from Mendelian

randomization studies, (ix) robust evidence only for circulating phosphate, leukocyte telomere length, and PCSK9 levels, and (x) poor concordance between meta-analysis findings and Menedelian randomization results.

With these data and analyses, the authors could confidently proffer only a limited amount of associations that were convincing (meta-analyses) or robust (Mendelian randomization). As such, a reasonable treatment of confounding was provided.

R: Thank you for your comments. We have updated the literature so there have been some changes in the main results.

The major challenge for all of the studies is the case definition: even though autopsies of men not known to have the disease reveal prostate cancer in more than a third of men as the age (see Jahn JL et al. Int J Cancer 137:2795-2802, 2015 and Bell KJL et al. Int J Cancer 157: 1749-1757, 2015), most of those cancers are not diagnosed. Most prostate cancers in the US and Europe are now screen-detected, so disease diagnosis inevitably reflects the likelihood that someone has been screened (and diagnosed) rather than the propensity to have suffered the onset of prostate cancer. Some analyses have attempted to circumvent this challenge by focusing of Gleason grade/score or on prostate cancer mortality. Were there any attempts by the current folks to do so?

R: We thank the reviewer for raising such an important question. We agree with the reviewer and the two researchers (Jahn JL et al. Int J Cancer 137:2795-2802, 2015 and Bell KJL et al. Int J Cancer 157: 1749-1757, 2015) that disease diagnosis inevitably reflects the likelihood that someone has been screened (and diagnosed) rather than the propensity to have suffered the onset of prostate cancer. We have taken notice of the prostate cancer classification (high-grade/advanced/lethal) in the existing literature and extracted the data when available. As shown in the S5 Table presenting the basic characteristics of included meta-analyses and evidence grading results), the asterisk (^*^) indicated that the metrics were for high-grade/advanced/lethal prostate cancer, which took up a small proportion of all the associations, many of which presenting null associations (please see the below picture of excerpt from S5 Table). Though limited, significant effects on high-grade/advanced/lethal prostate cancer were identified for several exposures including age at first intercourse, egg consumption, adult weight gain, androgenic alopecia, finasteride, and total cholesterol level.

W, White; A, Asian; RR, risk ratio; OR, odds ratio; HR, hazard ratio; SIR, standard incidence ratio; SRRE, summary relative risk estimate; NA, not available due to the incomplete reports of original study. Metrics with * denoting advanced, aggressive, high-grade or lethal prostate cancer, metrics with # denoting nonadvanced, nonaggressive or localized prostate cancer.

Another approach has been to limit analyses to cohorts where men underwent prostate biopsy as part of the study (versus 'for-cause'), such as in the Prostate Cancer Prevention Trial or the Selenium and Vitamin E Cancer Prevention Trial. Were data from these trials (one was mentioned in the manuscript text) examined separately?

R: Thank you for your question. we agree with the editor that data from trials should be examined separately, and there are scattered trials (usually less than 2) for several factors (statins (N=6), CCB, supplemental vitamin E intake, and aspirin, etc.) mainly in category diet and nutrition as well as drug target in category of medical conditions because the original meta-analysis included these trials as part of data synthesis. But in this present umbrella review, data from trials were not separately examined. The major reason is that data from RCT is limited, and it is not a main source of data in our study, neither influence the main results derived from cohort studies.

To sum up, relatively limited data on prostate cancer classification and data from trials restricted the analyses in detail in this present umbrella review. Further researches focusing on the two important aspects are warranted.
