# Supplementary material for: Risk factors for prostate cancer: An umbrella review of prospective observational studies and mendelian randomization analyses
Source: PLoS Med. 2024 Mar 15;21(3):e1004362. doi: 10.1371/journal.pmed.1004362 (PMC10980219; doi:10.1371/journal.pmed.1004362)
Supplement: Attachment — Submitted filename: PMEDICINE-D-23-01730R2-Point_by_Point_Responses.docx [file pmed.1004362.s015.docx]

**Authors’ Reponses to the Editors’ and Reviewers’ Comments**

(PMEDICINE-D-23-01730R2)

We would like to thank the reviewers and editors for their helpful comments and suggestions on our manuscript (PMEDICINE-D-23-01730R2). We have revised the manuscript point by point as requested. Our point-by-point responses (R) to the specific comments (Q) are provided below. All changes in our manuscript (marked up manuscript) are highlighted to facilitate review process.

**Requests from the editors:**

We have identified a BMJ Medicine guideline on umbrella reviews (http://dx.doi.org/10.1136/bmjmed-2021-000071) which states that “Researchers should use the study specific data extracted from each SRMA to repeat each meta-analysis separately rather than report the meta-analytical result as presented in the original SRMA.” It is not clear in your Methods section whether meta-analyses were repeated in your umbrella review. Please ensure that it is clearly states in your Methods whether meta-analyses were repeated. From lines 163-182, it appears that this has only been partially done, when a mix of observational studies were included in the original MA, and where studies do not provide “required information”.

Further to our recent correspondence by email, it is essential that the Methods section is revised to detail how meta-analyses were repeated in your review, and that assessment of quality is reconducted using a more appropriate tool. We cannot make any decision about publication until we have seen the revised manuscript and your response, and we plan to seek re-review by one or more of the reviewers.

R: Thank you for your suggestion. We agree with the editors that it is essential that the Methods section is revised to detail how meta-analyses were repeated in our review. Therefore, we revised the Methods section and provided a statistical analysis protocol as a supplementary text (S3 Statistical analysis protocol) to detail the statistical analyses. In addition, the forest plots of repeated meta-analyses for 46 significant associations have also been uploaded as supplementary figure (S1 Figure). S3 Statistical analysis protocol and S1 Figure are newly added supplementary information.

Below is the excerpt from the revised manuscript (Lines 168-186):

“A statistical analysis protocol in detail for this umbrella review was provided (S3 Statistical analysis protocol). In brief, in each included meta-analysis, qualified individual studies (cohort, case-cohort, or nested case-control study where exposure precedes the outcome) were selected, and relevant information were collected based on a predefined template: first author, publication year, study design, number of studies included, number of cases/population, ethnicity, exposure factors, outcomes of prostate cancer, comparisons, and effect estimates of any type, i.e., maximally adjusted hazard ratio (HR)/incidence rate ratio (IRR)/odds ratio (OR)/RR with 95% confidence intervals, i.e., lower confidence interval (LCI) and upper confidence interval (UCI). Then we repeated each meta-analysis based on extracted effect estimates, LCI, and UCI using the random-effects inverse-variance model with DerSimonian-Laird method. Heterogeneity between studies included in meta-analyses was represented using I square (I^2^) value and Cochrane’s Q P value [23]. I^2^<50% was considered as no or small heterogeneity, and I^2^ >50% large heterogeneity. Publication bias, or small study effect was evaluated by using the Egger regression asymmetry test (significance threshold, P<0.10) [24]. If the Egger’s P value was less than 0.1, we assumed the existence of publication bias. The 95% prediction interval (PI) estimated the middle 95% area of the predictive distribution and showed the range of true effects in future studies [25], reflecting the variation in the true effects across study settings. All statistical analyses were conducted with the use of Stata, version 14.0 (StataCorp), and R, version 3.3.0 (R Foundation for Statistical Computing).”

We note that you have referenced the original 11-item AMSTAR tool for assessing MAs of randomised studies. Since your umbrella review only includes non-randomised studies, please use another tool that is valid for non-randomised studies specifically. AMSTAR-2 would be acceptable if a tool designed specifically for assessing quality of non-randomised studies cannot be identified.

R: Thank you for your kind suggestion, and we agree with the editor that AMSTAR-2 serves as a more rational quality assessment tool for our review. As suggested, we have re-evaluated the quality of included meta-analyses using AMSTAR-2. The revised Table S3 presents the detailed assessment results using AMSTAR-2. Additionally, a new figure (Fig 2 Quality assessment of meta-analyses using AMSTAR-2) was added to the revised manuscript to demonstrate the percentage of each outcome.

Below is the excerpt from the revised manuscript (Lines 191-197; Lines 265-273):

“The online 16-item AMSTAR-2 (A MeaSurement Tool to Assess systematic Reviews) checklist was used to assess methodological quality [26]. AMSTAR-2 considers the quality of the search, study inclusion and exclusion, description of individual studies, assessment of publication bias, heterogeneity, use of appropriate statistical methods, assessment of risk of bias in individual studies, and reporting of sources of funding and conflicts of interest. The items were scored as No (0 points), Partial yes (0.5 points), or Yes (1 point). Both the total scores and critical item scores were calculated in our umbrella review [27].”

“The median of AMSTAR-2 total score was 13.5 points, and that for the critical item score was 6 points. For the 7 AMSTAR-2 critical domains, 26% of the included meta-analyses established a priori a protocol for the review, 90% performed a comprehensive literature search, 64% provided a list of excluded studies with justification, 84% used a satisfactory technique for assessing the risk of bias in individual studies, 90% used the appropriate model for meta-analysis, 67% discussed the impact of risk of bias in individual studies in the interpretation of the results of the review, and 78% performed graphical or statistical tests for publication bias and discussed the likelihood and magnitude of impact of publication bias (Fig 2). Each AMSTAR-2 domain judgment for each outcome is available in S3 Table.”

Comments from the reviewers

Reviewer #1: Thanks authors for their effort to improve the manuscript. I am mostly satisfied with the response and revision. However, the extra subgroup analysis results on white vs non-white HAVE NOT been included in S8 Table as the authors claimed

“Additionally, subgroup analyses between whites and non-whites population were performed upon request. The subgroup analyses results showed the effects remained largely consistent for white cohorts (S8 Table) and that number of researches on non-white population was very limited”

This is inadequate. Can authors make sure this subgroup analysis results are included somewhere in the paper?

R: Thank you for your kind comments. The subgroup analysis results are presented in S6 Table. We apologize for having put the wrong sequence number of table in the response letter, which should be S6 Table instead of S8 Table. We have made some changes to the sequence number of tables in the manuscript, however, we forgot to update S6 Table in the response letter. Additionally, we have provided S2 Fig as supplementary information to show the forest plots of subgroup analyses.

Reviewer #2: The authors have done a nice job addressing my reviewer comments. My remaining comments are as follows:

1. Only 3 factors showed consistent significant associations with prostate cancer in both meta-analyses and MR studies. Of these, smoking does not make sense, and the association between physical activity was not robust in MR accelerator-measured physical activity. Thus, height seems to be the only real positive factor that came out of this large umbrella review, which adds little novelty to the existing literature.

R: Thank you for your comments. First of all, we would like to comment that the primary finding that only 3 consistent significant associations were identified is based on extensive existing literature and supported by current data evidence. There could be several reasons underlying the very few consistent significant associations between meta-analysis and MR studies. First is the scarcity and unavailability of instrumental variables for most dietary factors and environmental exposures. Second is the limited strength of instrumental variables (IVs) for many factors with less than 10 associated single nucleotide polymorphisms as IVs. Thirdly, observational studies and MR studies have respective focuses and investigated factors in different categories. For example, MR studies investigated plentiful biomarkers while observational studies focused mainly on dietary factors, leaving a bulk of factors not able to be compared. As the researches on prostate cancer develop, we are expecting more and stronger IVs to be utilized in future investigations, and hopefully generating more consistent significant associations.

Secondly, regarding the seemingly unreasonable effect of smoking on prostate cancer, we referred to the related literature and carried out discussion in the manuscript. Smoking, albeit with consistent effect, should be taken prudently for its moderate and mixed observed effect. The positive association of smoking with prostate cancer in earlier years (before 1995) and with mortality collectively suggested that smoking might be linked with an increased risk of aggressive prostate cancer rather than indolent one (total prostate cancer). Current smoking was believed to be associated with a lower likelihood of prostate-specific antigen (PSA) testing, and individuals with a smoking history were less likely to undergo prostate biopsy. Consequently, the detection rate of prostate cancer could be relatively lower among participants in the PSA screening era. Another possible explanation is that smoking is the leading risk factor for death among males. Smokers may die from smoking attributable diseases before their diagnosis of prostate cancer. In addition, multiple inconsistent exposure categories for smoking such as current smoking, former smoking, and ever smoked, etc. might contribute to the varied results. Therefore, more elaborate investigations on smoking are warranted.

Thirdly, if we look at the findings of this umbrella review in the large, rather than focusing on the few consistent associations, we would like to remind that there are in total 123 associations identified from meta-analyses of prospective studies of which 46 significant ones were graded, providing valuable reference for understanding prostate cancer risk.

To conclude, we believe our efforts have made non-negligible contributions to the existing literature, deepening the understanding on prostate cancer and serving as useful reference and solid foundation for future researches.

2. The Table 6 subgroup analysis between white and non-white populations is barely mentioned in the Results, rather an off-hand comment is made that this was done to address reviewer comments. It is also not mentioned at all in the Discussion. Can these results be expanded upon in the results and also discussed with a paragraph in the discussion? For example, the association between dairy products and prostate cancer risk seems much greater in non-white populations (although this appears to be the result of a single study).

R: Thank you for your suggestions. We agree with the reviewer that the subgroup analysis results have not been adequately mentioned and discussed in Results and Discussion. Therefore, as suggested, we have expanded and discussed these results.

Below is the revised excerpt (Lines 283-289; Lines 515-527):

“Additionally, subgroup analyses of whites and non-whites population were performed for 11 significant associations (S6 Table and S2 Fig). Studies on non-white population were very limited (mostly less than 2) compared with those on white population. The subgroup analyses results showed the significant effects remained largely consistent for white population, while in non-white population only the inverse associations of smoking and finasteride remained significant. In addition, total dairy products showed stronger effects in non-white population, though supported by only one single study.”

“Second, most cohort studies were conducted in developed western countries, and hence findings of this current study are limited mainly for European descendants. Despite subgroup analysis performed by ethnicity, it was greatly limited by sparsity of data on non-white populations. Effects of different risk factors on prostate cancer may vary between ethnicities, which may be attributed to diverse genetic backgrounds and lifestyles. Data on prostate cancer incidence in most Asian countries might be statistically biased by the immature implementation of early screening practice and national cancer registry [42]. As prostate cancer is expected to rise in developing countries due to increased aging and popularity of PSA screening, data of non-white population are accumulating and await evaluation. Third, heterogenous effects based on prostate cancer classifications suggest both pathological variation of prostate cancer and diverse effects of exposure factors. For instance, smoking was found to be inversely associated with total prostate cancer, but its effect on aggressive prostate cancer appeared to be the opposite in some literature [43].”

3. Lines 262 through 265 in the Results should depict the actual numbers and percentages rather than just approximations. For example, “The study design contained mostly prospective cohort, with a small portion (less than 10%) of nested control study and case-cohort study”. What were the exact percentages of nested control studies and case-cohort studies, and how many such studies were included? How many studies were prospective? (Number and percentage).

R: Thank you for your valuable suggestion on data accuracy. We agree with the reviewer that actual number and percentages rather than approximations should be reported. As suggested, we have reported the exact numbers and percentages in the revised manuscript.

Below is the revised excerpt from the manuscript (Lines 261-264):

“The median of studies included in meta-analyses was 7, ranging from 2 to 35. The sample sizes of cases in meta-analysis ranged from 20 to 118,077. The study design contained mostly cohort studies (N=1,342, 95.7%), with a small portion of nested case-control studies (N=50, 3.6%), case-cohort studies (N=4, 0.2%), and randomized controlled trials (N=7, 0.5%).”
