# Supplementary material for: Risk factors for prostate cancer: An umbrella review of prospective observational studies and mendelian randomization analyses
Source: PLoS Med. 2024 Mar 15;21(3):e1004362. doi: 10.1371/journal.pmed.1004362 (PMC10980219; doi:10.1371/journal.pmed.1004362)
Supplement: Attachment — Submitted filename: PMEDICINE-D-23-01730R3-Point_by_Point_Responses.docx [file pmed.1004362.s016.docx]

**Authors’ Reponses to the Editors’ Comments**

(PMEDICINE-D-23-01730R3)

We would like to thank the editors for your helpful comments and kind suggestions on our manuscript (PMEDICINE-D-23-01730R3). We have revised the manuscript point by point as requested. Our point-by-point responses (R) to the specific comments (Q) are provided below. All changes in our manuscript (marked up manuscript) are highlighted to facilitate review process.

**Requests from the editors:**

GENERAL

1) Please replace hyphens with commas when reporting 95% CI values. The use of commas to separate upper and lower bounds, as opposed to hyphens, could be confused with reporting negative values.

R: Thank you for your kind suggestion. As suggested, we have replaced hyphens with commas when reporting 95% CI values in the manuscript, figures, tables, and supplementary materials wherever relevant.

2) We noted that the section on data extraction and synthesis for meta-analyses is very detailed, whereas this section for MR studies is rather short. Please revise to ensure that sufficient detail is provided for both approaches.

R: Thank you for your kind suggestion. For MR studies, we extracted the relevant result data from the original MR article, and no further data synthesis was done, which is why the section of data extraction and synthesis for MR studies is rather short. As suggested, we revised the data extraction and synthesis for MR studies to ensure that sufficient detail is provided for MR studies for clarity.

Lines 162-164: “The inclusion criteria for MR studies were similar but relatively concise: evaluating the factors of any category associated with prostate cancer using Mendelian randomized analysis methods.”

Lines 197-200: “From MR studies, we extracted key information of exposure, outcome, sample size, number of IVs, the variance (r^2^) explained by IV, F statistics, and maximally adjusted effect estimates with 95% CI using the main analysis method, and no further calculation was needed for MR studies in this umbrella review.”

3) Please provide the initials of the authors who searched the literature, assessed quality etc.

R: Thank you for your kind suggestion. As suggested, we have provided the initials of the authors who searched the literature, extracted data, and assessed quality in the revised manuscript.

Lines 152-153: “Following PRISMA [22], two researchers (HC and YQ) independently searched and screened related literature.”

Lines 185-186: “Data extraction was conducted by two researchers (HC and YQ) separately and cross-check was performed to ensure correctness.”

Lines 219-221: “Two researchers (HC and ZT) rated the methodological quality of meta-analyses and reporting quality of MR studies and evaluated the assumptions of MR studies. In the case of disagreements, a decision was reached by consulting a third investigator (WZ).”

4) We noticed that throughout the Results section, none of the included meta-analyses are referenced when discussing the relevant data. Please revise.

R: Thank you for your kind suggestion. As suggested, we have added references for the included meta-analyses as well as the included MR studies in the revised manuscript.

ABSTRACT

1) l.28: “The objective is to identify…” – please change to “The objective of this study was to identify…”

R: Thank you for your kind suggestion. We have made the revision as requested.

2) l.46: Please clarify what you mean by “outdated studies”.

R: Thank you for your kind suggestion. For the same exposure factor evaluated by more than one meta-analysis published in different years, we preferentially selected the most recent or updated one including the largest number of studies (cohorts or datasets) with the maximum of participants to represent the best available evidence. The overlapping and outdated meta-analyses which were published earlier and contained fewer cohorts or datasets compared with selected one were thus excluded. We have added further explanation for overlapping and outdated meta-analyses in the revised Abstract for clarity.

Lines 47-49: “Finally, 92 selected from 411 meta-analyses and 64 selected from 118 MR studies were included after excluding the overlapping and outdated studies which were published earlier and contained fewer participants for the same exposure factor.”

INTRODUCTION

l.107: “The earliest umbrella review on prostate cancer..” – please add “to our knowledge” or similar.

R: Thank you for your kind suggestion. We have made the revision as requested.

METHODS AND RESULTS

Please ensure that the manuscript is revised to improve clarity and conciseness. In particular, we feel that the presentation of values in the Results section is not detailed enough, and we have noted several instances where numbers do not add up.

R: Thank you for your kind suggestion. We have added more details in the presentation of values in the Results section as suggested and checked the sum of numbers.

1) l.222: Please change to “loosened effect P value threshold of <0.001”.

R: Thank you for your kind suggestion. We have made the revision as requested.

2) ll.228-229, please change to: Also, associations showing the presence of both large heterogeneity and high publication bias (I2≥50% and Egger’s P ≤0.1) would be graded as weak.

R: Thank you for your kind suggestion. We have made the revisions as requested.

3) ll.258-260: The numbers provided for the individual six major categories do not add up to 123 observational associations, but to 122. Please check carefully and revise.

R: Thank you for your question. The 123 observational associations included both the inverse association of finasteride with total prostate cancer and the positive association of finasteride with advanced prostate cancer, which we deemed as two different associations for distinction. Therefore, we counted the total number of associations as 123 though there were 122 factors.

4) ll.261-262: Please not only report upper and lower values, but a median with the interquartile range.

5) ll.261-264: When presenting the percentages of the various study designs included in the meta-analysis, it would be helpful to include the total number, i.e. the denominator, so that the calculation is understandable (e.g., “The study design contained mostly cohort studies (1,342 [95.7%] of XXXX)...”.)

6) ll.265-266. Please not only report the median, but also the 25th and 75th percentiles of the data, i.e. the interquartile range.

7) ll.266-271: Please not only report percentages, but also numerator and denominator in parentheses.

R (4,5,6, and 7): Thank you for your kind suggestions. We have reported the median with the interquartile range, the numerator, and denominator as suggested.

Lines 274-300: “The median (interquartile range, IQR) of studies (datasets) included in meta-analyses was 7 (4.25, 13), ranging from 2 to 35. The median (IQR) of case numbers in meta-analysis was 5,653 (2,735, 15,254), ranged from 20 to 118,077. The study design contained mostly cohort studies (N=1,342, 95.7% of 1,403), with a small portion of nested case-control studies (N=50, 3.6% of 1,403), case-cohort studies (N=4, 0.2% of 1,403), and randomized controlled trials (N=7, 0.5% of 1,403).

In total 90 eligible meta-analyses were assessed using AMSTAR-2 tool. The median (IQR) of AMSTAR-2 total score was 13.5 (13, 14) points, and that for AMSTAR-2 critical item score was 6 (5.5, 6) points. For the 7 AMSTAR-2 critical domains, 29% (26/90) of the included meta-analyses established a priori a protocol for the review, 100% (90/90) performed a comprehensive literature search, 71% (64/90) provided a list of excluded studies with justification, 93% (84/90) used a satisfactory technique for assessing the risk of bias in individual studies, 100% (90/90) used the appropriate model for meta-analysis, 74% (67/90) discussed the impact of risk of bias in individual studies in the interpretation of the results of the review, and 87% (78/90) performed graphical or statistical tests for publication bias and discussed the likelihood and magnitude of impact of publication bias (Fig 2). Each AMSTAR-2 domain judgment for each outcome is available in S3 Table.

In total 45 (of 123) significant associations (S1 Fig) were derived from 45 meta-analyses and subsequently graded, and the evidence grading details were elaborated in S4 Table. Among them, P values for summary effects were mostly between 0.001 and 0.05 (N=27, 60% of 45) and between 0.001 and 0.000001 (N=12, 27% of 45), while only six associations (N=6, 13% of 45) had P values less than 0.000001. Only three associations (N=3, 6.7% of 45) had case number of less than 1,000. Eleven associations (N=11, 24% of 45) had 95% PI excluding the null. Twenty-three (N=23, 51% of 45) associations reported presence of heterogeneity (I2>50%), and nine (N=9, 20% of 45) showed significant publication bias. In summary, there were five highly suggestive, 36 suggestive, and four weak associations in meta-analyses (S5 Table and Fig 4). The remaining 78 associations were null and not graded.”

8) l.274: For easy comprehension, we suggest repeating the total number of associations here. For example: “In total 46 (of 123) significant associations…”

R: Thank you for your kind suggestion. We have repeated the total number of associations as suggested.

9) l.274: We think it would be useful to mention the number of meta-analyses from which the 46 significant associations were derived.

R: Thank you for your kind suggestion. We have added the number of meta-analyses as suggested.

10) ll.283-285: Again, we think it would be useful to mention the number of meta-analyses from which the 11 significant associations were derived.

R: Thank you for your kind suggestion. We have added the number of meta-analyses as suggested.

11) l.285: “(mostly less than 2)” seems to be a vague statement. Could you quantify this?

R: Thank you for your kind suggestion. we have removed the vague statement and revised it.

Lines 303-305: “As shown in S6 Table, compared with those on white population, studies on non-white population were very limited: there was only one non-white study for 5 factors, and two non-white studies for 4 factors, and 3 for ulcerative colitis, and 4 for current smoking.”.

12) l.289: Please reference the one study and remove the word “single”.

R: Thank you for your kind suggestion. We have removed the word “single” and added the reference as suggested.

Lines 308-309: “In addition, total dairy products showed stronger effects in non-white population, though supported by only one study [119].”

13) l.290 and ongoing: When presenting data, be sure to reference the appropriate graph and/or table.

R: Thank you for your kind suggestion. We have referenced the graph and table when presenting data.

14) l.291 and ongoing: For clarity, please be sure to present the denominator when applicable. For example: “In total 17 lifestyle factors (of 123 total observations) were identified…”. Please revise throughout the entire main manuscript.

R: Thank you for your kind suggestion. we agree that the adding the denominator when applicable help with clarity. We have revised throughout the entire main manuscript.

15) l.338: Please change to “…with almost half..”.

R: Thank you for your kind suggestion. We have made the revision as requested.

16) l.338: We feel the term “medical condition factors” might not be appropriate for all factors included in this group, such as “sulfonylureas” or “regular use of aspirin”. We suggest changing this category to “clinical variables, treatments/procedures, and risk factors” or similar.

R: Thank you for your kind suggestion. Based on your suggestion, we have changed it into “clinical variables, diseases, and treatments” in all the documents wherever applicable.

17) ll.387 and ongoing: Please specify what “most” and “some” means, i.e. providing actual numbers.

R: Thank you for your kind suggestion. We have provided actual numbers.

Lines 413-420: “All studies used two-sample MR design, with European ancestry outcome data mostly from PRACTICAL (The Prostate Cancer Association Group to Investigate Cancer Associated Alterations in the Genome consortium) (N=113, 78% of 145 total associations). The median (IQR) of number of IVs was 13.5 (4, 54.25), ranging from 1 to 663. All studies were in line with the STROBE-MR, demonstrating good reporting quality. Concerning sensitivity analysis, there were 94 associations (65% of 145 total associations) reporting sensitivity analysis results. In total 55 significant causal associations (of 145 total associations) from MR studies were graded.”

18) ll.391-392: Please revise “There were 94 reported associations (65%) had sensitivity analysis.”.

R: Thank you for your kind suggestion. We have changed to “Concerning sensitivity analysis, there were 94 associations (65% of 145 total associations) reporting sensitivity analysis results.”.

19) l.395 and ongoing: Similar to the presentation of "Results of meta-analyses in categories", we suggest that under "Results of MR studies in categories" you should start each paragraph with the number of factors in each category. For example: l.390 " Concerning lifestyle factors (10/145)..."

R: Thank you for your kind suggestion. We have revised the presentation of “Results of MR studies in categories” by starting each paragraph with the total number of factors as well as the number of significant factors in each category.

20) l.396: Under “lifestyle factors” you only discuss 9 out 10 factors. What about the 10th factor?

R: Thank you for pointing out this error. We have added the 10th factor (cannabis).

21) l.407: Under “anthropometric indices” you only discuss 8 out 9 factors. What about the 9th factor?

R: Thank you for pointing out this error. We have added the 9th factor (favorable adiposity).

22) ll.418-419: What about the other 57 biomarkers? Please make sure that all factors are discussed, not all in detail, but at least as "remaining factors". Please revise throughout the main manuscript.

R: Thank you for your kind suggestion. We have revised as suggested.

23) l.420: Please see the comments about “medical condition factors” and revise accordingly (also in the Discussion section).

R: Thank you for your kind suggestion. We have made the revisions as suggested.

DISCUSSION

1) l.457: “more than 200” – please present the exact value.

R: Thank you for your kind suggestion. We have provided the exact value as “268”.

2) l.477: “These diseases..” – please specify.

R: Thank you for your kind suggestion. We have specified these diseases in the revised manuscript.

Lines 521-524: “Diseases such as melanoma, acne in adolescence, UC, infertility, prostatitis, and BPH associated with higher prostate cancer risk indicated shared biological mechanisms such as hormone dependency, inflammation [179], and genetic susceptibility [89, 93, 98].”

3) l.448: The evidence grading for height in MR studies was ‘insufficient’. We feel that the weak evidence in the MR studies is not very supportive of this association, please discuss it. Also, please discuss in more detail the existing literature on how height is implicated in cancer risk/mortality.

R: Thank you for your kind suggestion. We agree with the editors that the weak evidence in the MR studies for height is not very supportive of this association. We have discussed it in the revised manuscript. Also, the existing literature on how height is implicated in cancer risk/mortality is discussed in more detail as suggested.

Lines 536-546: “First, concerning height, MR analyses on height provided insufficient evidence of its causal association with prostate cancer, in addition to inconsistent results from other identified MR studies [180-182], which is not very supportive of this association. Height is implicated in many biological pathways such as skeletal growth, fibroblast growth factor (FGF) signaling, WNT (Wingless / Integrated) signaling, regulation of β-catenin, mammalian target of rapamycin (mTOR) signaling [183] and associates with overall cancer risk and mortality [180]. A plausible mechanism involves dietary programming of the IGF-1, which plays an important role in the regulation of postnatal growth and is also associated with prepubertal growth in height [184]. Thus, the variations in the IGF-1 system might underlie associations of height with prostate cancers that are more likely to progress [185]. Still the causal mechanism of height in progressive prostate cancer needs further investigation.”

4) ll.490-491: Please define ‘FGF’, ‘WNT’ and ‘mTOR’.

R: Thank you for your kind suggestion. We have defined “FGF” (fibroblast growth factor), “WNT” (Wingless / Integrated), and “mTOR” (mammalian target of rapamycin) in the revised manuscript.

5) l.505: Please discuss in more detail the existing literature on how physical activity is implicated in cancer risk/mortality.

R: Thank you for your kind suggestion. we have expanded the discussion on how physical activity is implicated in cancer risk/mortality.

Lines 559-574: “Concerning physical activity, physical activity may be associated with cancer through several pathways related to oxidative stress, DNA methylation, telomere length, immune function, and gut microbiome [190]. Shorter duration aerobic physical activity stimulates short-term increases in immunoglobulins, neutrophils, natural killer cells, cytotoxic T cells, and immature B cells, which over time, enhance immunosurveillance [191]. Physical activity reduces adipose tissue and correcting metabolic abnormalities, which has been shown to reduce plasma insulin and increase insulin sensitivity and glucose metabolism, thereby lowering the risk of certain cancers [192]. In terms of cancer progression, physical activity may predispose to biologically less aggressive tumors and may improve functional capacity to tolerate and complete cancer treatment, thereby slowing down cancer progression [193]. The results regarding physical activity in this umbrella review are not robust because accelerator-measured physical activity showed a protective effect on prostate cancer in MR but with very weak instrumental variable explaining only 0.1% of the variance. In addition, in meta-analyses occupational physical activity was graded as highly suggestive evidence but overall physical activity showed null associations with prostate cancer, possibly attributed to differed measurement.”

6) ll.544-547: We feel that the conclusion section is rather general and does not discuss that the comparison between observational associations by meta-analysis and genetically estimated causality by MR does not provide robust evidence due to the lack of overlapping observations as well as the lack of high-quality evidence, especially in MR studies. Please revise.

R: Thank you for your kind suggestion. We agree with the editors that the conclusion part should point out that the comparison does not provide robust high-quality evidence due to the lack of overlapping observations as well as the lack of high-quality evidence, especially in MR studies. We have revised the conclusion section as suggested.

Lines 609-613: “In summary, this umbrella review provides a comprehensive evaluation on risk factors associated with prostate cancer as well as large-scale comparison between observational associations by meta-analysis and genetically estimated causality by MR analyses. Though no robust association is identified due to the lack of overlapping robust evidence based on existing literature, future researches are warranted to further our understanding on prostate cancer risk.”

FIGURES AND TABLES

1) Table 1: Please change 'Egger P value' to 'Egger's P value'. We also suggest that the result of 'Egger's p-value' be consistently described as 'publication bias' instead of switching between 'publication bias' and 'small study effects'.

R: Thank you for your kind suggestion. We have made the revisions as suggested.

2) Figure 1: Please define what “outdated” means.

R: Thank you for your kind suggestion. For the same exposure factor evaluated by more than one meta-analysis published in different years, we preferentially selected the most recent or updated one including the largest number of studies (cohorts or datasets) with the maximum of participants to represent the best available evidence. The overlapping and outdated meta-analyses which were published earlier and contained fewer cohorts or datasets compared with selected one were thus excluded. We have added further explanation in the figure description for clarity.

3) Figure 2: Please mention in the figure description the total number of meta-analyses included. Please consider avoiding the use of red and green in order to make your figure more accessible to those with colour blindness.

R: Thank you for your kind suggestion. As suggested, we have added the total number of meta-analyses included in the figure description, and the colors have been changed to make it more accessible.

4) Figure 3: Please in the figure description, define the numerical value presented in the two graphs. For example: Numbers presented in the graphs are Odds Ratio with 95% confidence intervals. Also: “¶ represents significant associations” – does significance equal p<0.05? Please revise.

R: Thank you for your kind suggestion. We have added the suggested explanations in figure description.

REFERENCES

Please thoroughly revise all references and ensure that journal name abbreviations match those found in the National Center for Biotechnology Information (NCBI) databases (http://www.ncbi.nlm.nih.gov/nlmcatalog/journals), and are appropriately formatted and capitalised (e.g., for reference [3] Annals of oncology: official journal of the European Society for Medical Oncology should be Ann Oncol)

R: Thank you for your kind suggestion. we have revised all references to ensure that journal name abbreviations match those found in the National Center for Biotechnology Information (NCBI) databases (http://www.ncbi.nlm.nih.gov/nlmcatalog/journals), and are appropriately formatted and capitalized.

SUPPLEMENTARY MATERIAL

1) S1 Fig: Please add in the figure description, that values are presented as Risk Ratios (RR) with 95% confidence intervals (95% CI). Also, please choose a proper heading for each graph (e.g. “egg” does not seem appropriate, better: Egg consumption) and we suggest adding parenthesis the category of each factor (lifestyle, biomarker etc.).

R: Thank you for your kind suggestion, which helps make S1 Fig more comprehensible. We have added the suggested figure description and the heading for each graph indicating the category, factor, and the comparison.

2) S2 Fig: Please change the title of the figure to: Forest plots of subgroup analyses according to ethnicity (white versus non-white). Please define all abbreviations used in the graphs (TE, seTE, CI etc.). Also, please define the meaning of the dashed lines.

R: Thank you for your kind suggestion, which helps make S2 Fig more comprehensible. We have changed the title as suggested and define all abbreviations used in the graphs and meaning of dashed lines. Also, we have added the explanations for the abbreviations used in the graphs.

“TE: Estimate of treatment effect, e.g., log hazard ratio or risk difference; seTE: standard error of TE; CI: confidence interval. The two dashed line indicated the odds ratios derived from the common effect model (the left dashed line) and from random effects model (the right dashed line), respectively.”

3) S6 Table: Please in the footnotes, be sure to add details about the exposure listed. For example: Firefighter: ever employment as a career firefighter vs general population.

R: Thank you for your kind suggestion. We have added details about the exposure listed as suggested in the footnotes.

4) S7 Table: Please check whether footnote a is correctly formatted as footnote in the table.

R: Thank you for your kind suggestion. We have made revision to footnote a as “ ^§^ denoting the exposure population source was of Asian ancestry or mixed ancestry.”.

SOCIAL MEDIA

To help us extend the reach of your research, please provide any X (formerly known as Twitter) handle(s) that would be appropriate to tag, including your own, your coauthors’, your institution, funder, or lab. Please respond to this email with any handles you wish to be included when we tweet this paper.

R: Thank you for your kind suggestion. We are sorry we do not have such X handle to provide.
