# Supplementary material for: Risk factors for prostate cancer: An umbrella review of prospective observational studies and mendelian randomization analyses
Source: PLoS Med. 2024 Mar 15;21(3):e1004362. doi: 10.1371/journal.pmed.1004362 (PMC10980219; doi:10.1371/journal.pmed.1004362)
Supplement: Attachment — Submitted filename: PMEDICINE-D-23-01730R4-Point_by_Point_Responses.docx [file pmed.1004362.s017.docx]

**Authors’ Reponses to the Editors’ Comments**

(PMEDICINE-D-23-01730R4)

We would like to thank the editors for the kind suggestions on our manuscript (PMEDICINE-D-23-01730R4). We have revised the manuscript point by point as requested. Our point-by-point responses (R) to the specific comments (Q) are provided below. All changes in our manuscript (marked up manuscript) are highlighted to facilitate review process.

Requests from Editors:

1) Abstract conclusion: You state that height, physical activity, and smoking had consistently significant effect on the risk of prostate cancer between meta-analyses and MR studies which we think might be misleading as an overall conclusion. Rather, we feel that the comparison between observational associations by meta-analysis and genetically estimated causality by MR does not provide robust evidence due to the lack of overlapping observations as well as the lack of high-quality evidence. Please revise this statement. Editorial suggestion: In this umbrella review, we summarized the associations of various factors with prostate cancer risk and provided comparisons between observational associations by meta-analysis and genetically estimated causality by MR analyses. In the absence of overlapping robust evidence based on the existing literature, no robust associations were identified, but some effects were observed for height, physical activity, and smoking.

R: Thank you for your kind suggestion. We agree with the editorial suggestion and have revised the conclusions as suggested: “In this large-scale study, we summarized the associations of various factors with prostate cancer risk and provided comparisons between observational associations by meta-analysis and genetically estimated causality by Mendelian randomization analyses. In the absence of overlapping strong evidence based on the existing literature, no robust associations were identified, but some effects were observed for height, physical activity, and smoking”.

2) ll.231-233: Please change to: “Highly suggestive evidence, with the largest component study requirement removed, required a loosened effect P value threshold of <0.001…” (the previous comment was regarding the less-than sign).

R: Thank you for your kind suggestion. We have revised as suggested.

3) l.228: The cut-off values for small/large heterogeneity and publication bias need to be revised so that the values of 50% (for heterogeneity) and p=0.1 (for Eggers) are not excluded. For example, no or small heterogeneity should be defined as I²≤50%, or large heterogeneity should be defined as I²≥50%. The same applies to publication bias, i.e. no publication bias should be defined as Egger’s P≥0.1 or publication bias as P≤0.1. Please revise in the text and Table 1.

R: Thank you for your kind suggestion. We have revised in the text and Table 1 as suggested.

4) “The 123 observational associations included both the inverse association of finasteride with total prostate cancer and the positive association of finasteride with advanced prostate cancer, which we deemed as two different associations for distinction. Therefore, we counted the total number of associations as 123 though there were 122 factors.” – please briefly include this information in the main text. We suggest you insert it following line 273.

R: Thank you for your kind suggestion. We have added the information following line 273 as suggested.

5) ll.304-305, please change to: “there was only one study of five factors, two studies of 4 factors, 3 for ulcerative colitis, and 4 for current smoking on non-white populations.” Please insert references to these studies.

R: Thank you for your kind suggestion. We have revised the sentence and provided references to these studies as suggested.

6) Please revise references [18], [26], [29], [124], [126], [152], [162], [166], [169], [189],

– the text following the PMICD number should be removed. Please carefully check all references for their correct formatting.

R: Thank you for your kind reminder. We have removed the text following the PMICD number and checked all the references for the correct formatting.

7) Figure 2: Please add to the figure description the definitions of ‘Yes’, ‘Partial Yes’ and ‘No’ as done in S3 Table.

R: Thank you for your kind suggestion. We have added the descriptions as suggested.

8) Figure 3: In the figure description, please add the definition of the asterisk in graph (A) and the hash in graph (B).

R: Thank you for your kind suggestion. We have added the definition of the asterisk in graph (A) and the hash in graph (B) as suggested.

9) Figure 6: Please define ‘NA’ under abbreviations.

R: Thank you for your kind suggestion. We have defined “NA” under abbreviations as suggested.

10) S1 Fig/S2 Fig: It seems for some of the graphs bits of text and numbers are overlapping. For example, for the first graph in S1 Fig (Regular use of aspirin), it is following the test for subgroup differences (fixed effects). Please carefully check so that the graphs are properly displayed. Also, please define the meaning of the differently dashed lines (densely and loosely dashed). Please note that “left dashed line” and “right dashed line” are not appropriate definitions. Please add the definitions of abbreviations to both figure descriptions.

R: Thank you for your kind suggestions. We have replaced the graphs, defined the dashed lines, and added definitions of abbreviations to both figure descriptions as suggested.

11) Please note that the file for S3 Statistical Analysis Protocol was not provided.

R: Thank you for your kind reminder. We have checked to ensure that all the supplementary files are provided.
